# Design, synthesis, biological evaluation and molecular docking study of 2,4-diarylimidazoles and 2,4-bis(benzyloxy)-5-arylpyrimidines as novel HSP90 N-terminal inhibitors

**DOI:** 10.1080/14756366.2022.2124407

**Published:** 2022-09-19

**Authors:** Man Yang, Chenyao Li, Yajing Li, Chen Cheng, Meiyun Shi, Lei Yin, Hongyu Xue, Yajun Liu

**Affiliations:** School of Life and Pharmaceutical Sciences, Dalian University of Technology, Panjin, China

**Keywords:** HSP90, 2 4-diarylimidazoles, 2 4-bis(benzyloxy)-5-arylpyrimidines, molecular docking, anticancer

## Abstract

The molecular chaperone HSP90 plays an essential role in cancer occurrence and development. Therefore, it is an important target for the development of anticancer drugs. 1,3-Dibenzyl-2-aryl imidazolidine (**8)** is a previously reported inhibitor of HSP90; however, its anticancer activity is poor. In this work, chemical modification of **8** led to the discovery of 2,4-diarylimidazoles and 2,4-bis(benzyloxy)-5-arylpyrimidines as two types of novel HSP90 N-terminal inhibitors. **16l** and **22k** exhibited antiproliferative activity against multiple breast cancer cell lines with IC_50_ values at the low micromolar level. **16l** and **22k** induced significant degradation of the client proteins AKT and ERK and a lower level of the heat shock response in comparison with tanespimycin (17-AAG). **22k** exhibited a strong affinity for the HSP90α N-terminus with an IC_50_ value of 0.21 μM. A molecular docking study revealed that **16l** and **22k** successfully bind to the geldanamycin binding site at the N-terminus of HSP90α.

## Introduction

Because proteins play roles in nearly every cellular process, it is essential to maintain protein homeostasis to preserve normal cell functions. Molecular chaperones are a large family of proteins that guard cellular protein homeostasis by regulating the conformation and quality of client proteins^1^^,^^2^. Heat shock protein 90 (HSP90) is one of the most crucial molecular chaperones in eukaryotes and stabilises and activates more than 400 client proteins^3^^,^^4^. Because cancer cells require higher levels of proteins for survival than normal cells, HSP90 is overexpressed in cancer cells, accounting for 4–6% of the whole proteome^5^^,^^6^. In addition, conformations of normal HSP90 and HSP90 of the cancer phenotype are different, and the latter is more susceptible to inhibitors^7^. Inhibition of HSP90 in cancer cells results in the degradation of client oncoproteins via the ubiquitin-proteasome pathway and the subsequent disruption of multiple signal transduction pathways, further leading to the apoptosis of cancer cells^8^^,^^9^. Therefore, HSP90 is a promising therapeutic target for discovering anticancer drugs^10^. Beyond cancer, HSP90 has also emerged as a potential drug target in other protein-related diseases, such as neurodegenerative diseases, infectious diseases, and ageing^11–14^.

HSP90 consists of three domains: the N-terminus, C-terminus, and the middle domain^15^^,^^16^. Classical HSP90 inhibitors competitively bind to the ATP binding pocket at the N-terminus. Over twenty HSP90 N-terminal inhibitors have entered clinical trials for the treatment of a variety of cancers^17^^,^^18^. Allosteric binding sites are also found at the C-terminus and the middle domain. HSP90 C-terminal inhibitors have been extensively studied in recent years because they do not cause a rescue cascade known as the heat shock response, which is often observed in the modulation of HSP90 with N-terminal inhibitors^19^^,^^20^. Many natural products and synthetic small molecules have been identified as HSP90 C-terminal inhibitors; however, they have not yet entered clinical trials for cancer therapy^21^.

**Figure 1.**
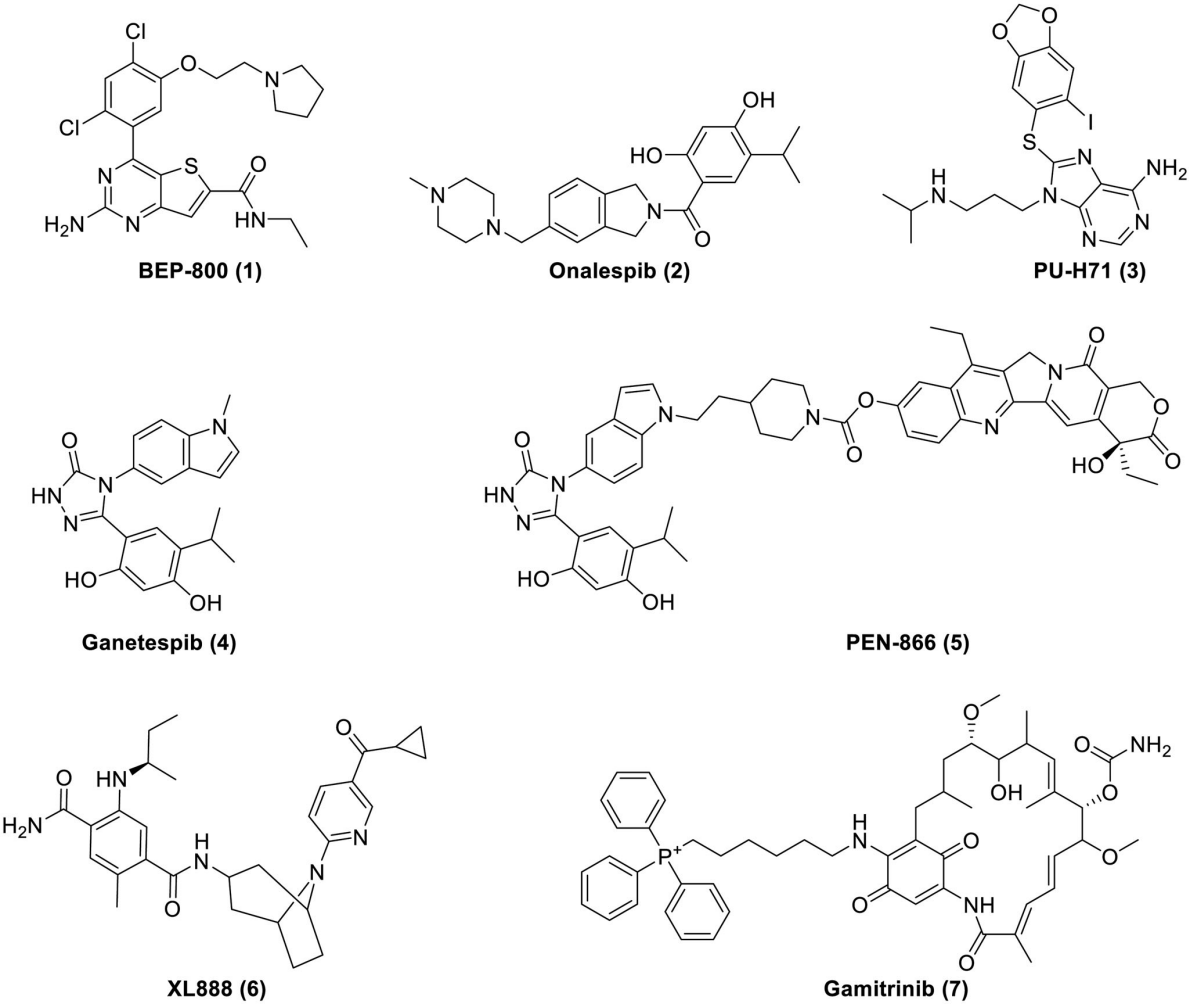
Representative HSP90 N-terminal inhibitors in clinical trials.

Although some clinical investigations of HSP90 N-terminal inhibitors have stopped or terminated because of drug resistance and/or organ toxicity, there are still a considerable number of active inhibitors in clinical trials^22^^,^^23^. According to the results obtained from ClinicalTrial.gov (https://www.clinicaltrials.gov/, 2022/06/17), 17 studies are ongoing or in preparation (including recruiting, enrolling by invitation, and active/not recruiting). Some representative examples (**1**–**7**) currently being evaluated in clinical trials are shown in Figure 1. It should be noted that combination therapy of HSP90 N-terminal inhibitors with other anticancer drugs represents an effective strategy to combat cancer in clinical trials at present^24^. Combination with HSP90 inhibitors would help to prevent the chemotherapeutic resistance of classical anticancer drugs and/or potentiate the cytotoxic effects^25^^,^^26^. In this context, it is still desirable to develop more HSP90 N-terminal inhibitors as novel anticancer agents.

**Figure 2.**
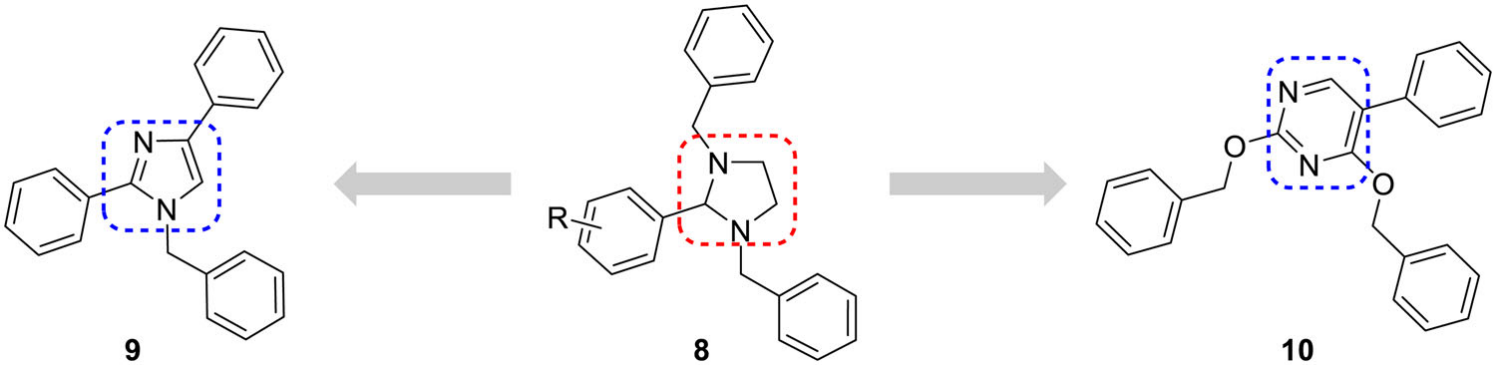
Design strategy illustration.

We previously reported that a series of 1,3-dibenzyl-2-aryl imidazolidines **8** served as HSP90 N-terminal inhibitors^27^ (Figure 2). These inhibitors showed a strong affinity for the HSP90 N-terminus according to the fluorescence polarisation (FP) assay; however, they exhibited weak antiproliferative activity against cancer cells such as MCF-7 and A549. Weak anticancer efficacy may be attributed to the physicochemical instability of the imidazolidine ring because **8** is converted into the corresponding benzaldehyde and *N*, *N’*-dibenzyl ethylenediamine in an aqueous medium^28–30^. Therefore, we hypothesised that replacing imidazolidine with stable aromatic scaffolds would lead to the discovery of novel HSP90 N-terminal inhibitors with stronger anticancer activity. Therefore, the nonaromatic imidazolidine ring was replaced by the aromatic imidazole ring and pyrimidine ring, which are frequently used in clinical drugs^31^^,^^32^. As shown in Figure 2, trisubstituted imidazole **9** and pyrimidine **10** were designed to develop novel HSP90 inhibitors. Compound **9** bears a benzyl group at the N1 position and two phenyl groups at the C2 and C4 positions of the imidazole ring. In the case of **10**, it has two benzyloxy groups at the C2 and C4 positions and a phenyl group at the C5 position of the pyrimidine ring.

## Materials and methods

### Chemistry

#### General method for chemistry

All chemical reagents were commercially purchased and used without further purification. Reactions were monitored by thin layer chromatography on GF254 TLC plates. Column chromatography purification was performed on silica gel (200–300 mesh). Nuclear magnetic resonance (NMR) data were collected on an AVANCE III HD 500 MHz nuclear magnetic resonance spectrometer (Bruker, Billerica, MA, USA). HRMS detection of **16** and **22** were carried out on a Q Exactive mass spectrometer (Thermo Fisher, Waltham, MA USA) with electrospray ionisation (ESI) as the ionisation source. Mass spectra of **19** and **20** were recorded by a UPLC–ESI–MS/MS system. The UPLC–ESI–MS/MS system consists of an Acquity UPLC system (Waters Corp., MA, USA) coupled with a QTRAP 6500 Plus mass spectrometer (Sciex, Toronto, Canada) equipped with a TurboIonSpray source. Analyst Software 1.6.3 was used for data acquisition and data processing.

#### Preparation of 1-benzyl-4-bromo-1*H*-imidazoles (13)

**13** was synthesised using a modified reported method^33^. To a solution of 4-bromo-1*H*-imidazole (**11**, 8.00 g, 54.40 mmol) in acetone (70 ml), benzyl bromide (**12**, 7.20 ml, 60.40 mmol) and potassium carbonate (8.28 g, 60.00 mmol) were added. The reaction mixture was stirred at room temperature for 22 h. Potassium carbonate was filtered, and acetone was removed under a vacuum. Water and ethyl acetate were added to extract the product. The organic layer was collected, washed with water and brine, dried over anhydrous magnesium sulphate, and condensed under a vacuum. The resulting crude product was dissolved in a small amount of dichloromethane and then precipitated by the addition of petroleum ether. The solid was filtered to afford **13** as a white solid (5.71 g, 44.07%).

#### General synthetic procedure for 1-benzyl-4-aryl-1*H*-imidazoles (14)

**14** was synthesised using a modified procedure^34^. To a solution of 1-benzyl-4-bromo-1*H*-imidazoles (**13**, 2.38 g, 10 mmol) in 1,4-dioxane (18 ml), aryl boronic acid (2.5 equiv.), Pd(dppf)Cl_2_ (5 mol%) and aqueous Na_2_CO_3_ solution (2.12 g in 6 ml water, 20 mmol) were added. The reaction mixture was heated to reflux and stirred under a nitrogen atmosphere for 4 h. After cooling to room temperature, the reaction mixture was extracted with water and ethyl acetate. The organic layer was collected, washed with water and brine, dried over anhydrous magnesium sulphate, and condensed under a vacuum. The resulting crude product was purified by column chromatography using petroleum ether/ethyl acetate as the eluent.

#### General synthetic procedure for 1-benzyl-2-bromo-4-aryl-1*H*-imidazoles (15)

**15** was prepared using a modified reported method^35^. To a solution of 1-benzyl-4-aryl-1*H*-imidazoles (**14**) in CCl_4_ (2.0 M), *N*-bromosuccinimide (NBS, 2 equiv.) was added. The reaction mixture was stirred at 35 °C for 14 h. The reaction mixture was extracted with dichloromethane and water. The organic layer was collected, washed with water and brine, dried over anhydrous magnesium sulphate, and condensed under a vacuum. The resulting crude product was purified by column chromatography using petroleum ether/ethyl acetate as the eluent.

#### General synthetic procedure for 1-benzyl-2,4-diaryl-1*H*-imidazoles (16)

**16** was prepared using a procedure similar to that for **14**. To a solution of 1-benzyl-2-bromo-4-aryl-1*H*-imidazoles (**15**) in 1,4-dioxane, aryl boronic acid (2.5 equiv.), Pd(dppf)Cl_2_ (5 mol%) and aqueous Na_2_CO_3_ solution (3.3 M, 2.5 equiv.) was added. The reaction mixture was heated to reflux and stirred under a nitrogen atmosphere for 4 h. After cooling to room temperature, the reaction mixture was extracted with water and ethyl acetate. The organic layer was collected, washed with water and brine, dried over anhydrous magnesium sulphate, and condensed under a vacuum. The resulting crude product was purified by column chromatography using petroleum ether/ethyl acetate as the eluent.

##### 1-Benzyl-2,4-diphenyl-1*H*-imidazole (16a)

White solid, yield 29.13%. ^1^H NMR (500 MHz, Chloroform-*d*) δ 7.65 (s, 1H), 7.51–7.49 (m, 2H), 7.43–7.35 (m, 3H), 7.28–7.18 (m, 7H), 7.14–7.11 (m, 1H), 6.97 (d, *J* = 7.0 Hz, 2H), 4.97 (s, 2H). ^13^C NMR (126 MHz, Chloroform-*d*) δ 138.32, 137.12, 136.59, 134.54, 130.96, 130.56, 128.92, 128.82, 128.79, 128.71, 128.12, 127.91, 126.93, 126.52, 126.33, 48.77. HRMS (ESI, m/z) calcd for C_22_H_19_N_2_ [M + H]^+^ 311.1548, found 311.1560.

##### 1-Benzyl-4-phenyl-2-(*m*-tolyl)-1*H*-imidazole (16b)

White solid, yield 35.28%. ^1^H NMR (500 MHz, Chloroform-*d*) δ 7.64 (d, *J* = 3.0 Hz, 1H), 7.54–7.49 (m, 2H), 7.28–7.25 (m, 4H), 7.23–7.17 (m, 3H), 7.23–7.10 (m, 4H), 7.03 (d, *J* = 8.0 Hz, 1H), 6.98 (s, 3H), 4.94 (s, 2H), 2.29 (s, 3H). ^13^C NMR (126 MHz, Chloroform-*d*) δ 138.57, 137.97, 136.95, 136.62, 134.48, 131.58, 130.35, 129.51, 129.03, 128.76, 128.12, 127.90, 127.05, 126.45, 126.31, 48.85, 21.33. HRMS (ESI, m/z) calcd for C_23_H_21_N_2_ [M + H]^+^ 325.1705, found 325.1715.

##### 1-Benzyl-2–(3-chlorophenyl)-4-phenyl-1*H*-imidazole (16c)

White solid, yield 32.46%. ^1^H NMR (500 MHz, Chloroform-*d*) δ 7.68 (s, 1H), 7.49–7.45 (m, 2H), 7.38–7.36 (m, 1H), 7.36–7.25 (m, 4H), 7.25–7.19 (m, 2H), 7.17–7.16 (m, 2H), 7.09–7.06 (m, 1H), 6.98–6.95 (m, 2H), 4.97 (s, 2H). ^13^C NMR (126 MHz, Chloroform-*d*) δ 138.90, 137.46, 136.14, 134.68, 133.97, 132.34, 130.88, 130.15, 129.28, 128.95, 128.89, 128.24, 128.12, 127.28, 126.96, 126.70, 49.09. HRMS (ESI, m/z) calcd for C_22_H_18_ClN_2_ [M + H]^+^ 345.1159; found 345.1172.

##### 1-Benzyl-4-phenyl-2-(*p*-tolyl)-1*H*-imidazole (16d)

White solid, yield 41.00%. ^1^H NMR (500 MHz, Chloroform-*d*) δ 7.62 (s, 1H), 7.51 (d, *J* = 7.5 Hz, 2H), 7.29–7.25 (m, 3H), 7.22–7.09 (m, 7H), 7.02–6.97 (m, 2H), 4.95 (s, 2H), 2.40 (s, 3H). ^13^C NMR (126 MHz, Chloroform-*d*) δ 138.61, 138.10, 136.98, 136.74, 134.67, 130.79, 129.67, 128.91, 128.78, 128.10, 127.86, 127.42, 126.91, 126.46, 126.24, 48.63, 21.38. HRMS (ESI, m/z) calcd for C_23_H_21_N_2_ [M + H]^+^ 325.1705, found 325.1714.

##### 1–(4-(1-Benzyl-4-phenyl-1*H*-imidazol-2-yl)phenyl)ethan-1-one (16e)

White solid, yield 64.45%. ^1^H NMR (500 MHz, Chloroform-*d*) δ 7.97–7.91 (m, 2H), 7.69 (s, 1H), 7.48–7.43 (m, 2H), 7.33–7.26 (m, 5H), 7.24–7.15 (m, 3H), 7.00–6.93 (m, 2H), 5.01 (s, 2H), 2.62 (s, 3H). ^13^C NMR (126 MHz, Chloroform-*d*) δ 197.56, 139.35, 137.92, 136.88, 136.26, 135.51, 134.10, 131.06, 128.92, 128.80, 128.27, 128.10, 127.57, 126.84, 126.76, 48.99, 26.68. HRMS (ESI, m/z) calcd for C_24_H_21_N_2_O [M + H]^+^ 353.1654, found 353.1661.

##### 1-Benzyl-2–(4-chlorophenyl)-4-phenyl-1*H*-imidazole (16f)

White solid, yield 34.40%. ^1^H NMR (500 MHz, Chloroform-*d*) δ 7.67 (s, 1H), 7.50–7.45 (m, 2H), 7.35–7.32 (m, 2H), 7.29–7.26 (m, 3H), 7.22–7.20 (m, 2H), 7.17–7.11 (m, 3H), 6.97–6.95 (m, 2H), 4.97 (s, 2H). ^13^C NMR (126 MHz, Chloroform-*d*) δ 138.86, 137.50, 136.38, 134.85, 134.22, 132.26, 129.23, 129.02, 128.88, 128.23, 128.03, 127.42, 126.77, 126.59, 48.84. HRMS (ESI, m/z) calcd for C_22_H_18_ClN_2_ [M + H]^+^ 345.1159, found 345.1171.

##### 4–(1-Benzyl-4-phenyl-1*H*-imidazol-2-yl)phenol (16g)

White solid, yield 37.00%. ^1^H NMR (500 MHz, DMSO-*d_6_*) δ 9.73 (s, 1H), 7.90 (s, 1H), 7.45–7.40 (m, 2H), 7.30–7.22 (m, 3H), 7.18 (t, *J* = 7.5 Hz, 2H), 7.09 (t, *J* = 7.3 Hz, 1H), 7.00 (d, *J* = 8.5 Hz, 2H), 6.93 (t, *J* = 7.5 Hz, 2H), 6.80 (t, *J* = 8.5 Hz, 2H), 5.02 (s, 2H). ^13^C NMR (126 MHz, DMSO-*d_6_*) δ 158.17, 138.01, 137.88, 137.13, 135.53, 132.45, 128.96, 128.47, 127.90, 127.27, 126.29, 126.14, 120.93, 116.30, 48.10. HRMS (ESI, m/z) calcd for C_22_H_19_N_2_O [M + H]^+^ 327.1497, found 327.1493.

##### 1-Benzyl-2-(furan-2-yl)-4-phenyl-1*H*-imidazole (16h)

White solid, yield 19.07%. ^1^H NMR (500 MHz, Chloroform-*d*) δ 7.64 (s, 1H), 7.58 (d, *J* = 7.5 Hz, 2H), 7.53 (d, *J* = 1.8 Hz, 1H), 7.34–7.19 (m, 6H), 7.06 (d, *J* = 7.0 Hz, 2H), 6.46–6.44 (m, 1H), 6.32 (d, *J* = 3.0 Hz, 1H), 5.08 (s, 2H). ^13^C NMR (126 MHz, Chloroform-*d*) δ 143.45, 143.24, 141.88, 138.10, 136.28, 134.07, 128.82, 128.24, 127.06, 127.04, 126.75, 118.85, 112.47, 111.29, 49.36. HRMS (ESI, m/z) calcd for C_20_H_17_N_2_O [M + H]^+^ 301.1341, found 301.1349.

##### 1-Benzyl-4-phenyl-2-(thiophen-2-yl)-1*H*-imidazole (16i)

White solid, yield 16.80%. ^1^H NMR (500 MHz, Chloroform-*d*) δ 7.67 (s, 1H), 7.60–7.54 (m, 2H), 7.45 (dd, *J* = 5.0, 1.5 Hz, 1H), 7.31–7.22 (m, 2H), 7.21–7.15 (m, 1H), 7.07 (dd, *J* = 5.0, 3.5 Hz, 1H), 7.05–7.00 (m, 2H), 6.93 (dd, *J* = 3.5, 1.1 Hz, 1H), 5.02 (s, 2H) . ^13^C NMR (126 MHz, DMSO-*d_6_*) δ 136.10, 133.17, 131.78, 129.38, 125.75, 125.59, 124.10, 123.88, 123.45, 123.24, 122.87, 122.19, 122.02, 121.85, 116.09, 44.07. HRMS (ESI, m/z) calcd for C_20_H_17_N_2_S [M + H]^+^ 317.1112, found 317.1123.

##### 1-Benzyl-2–(4-chlorophenyl)-4-(*p*-tolyl)-1*H*-imidazole (16j)

White solid, yield 47.54%. ^1^H NMR (500 MHz, Chloroform-*d*) δ 7.67 (s, 1H), 7.36–7.32 (m, 4H), 7.29–7.25 (m, 3H), 7.12 (d, *J* = 8.5 Hz, 2H), 7.03 (d, *J* = 8.0 Hz, 2H), 7.00–6.94 (m, 2H), 4.96 (s, 2H), 2.29 (s, 3H). ^13^C NMR (126 MHz, Chloroform-*d*) δ 138.77, 137.34, 136.34, 136.32, 134.81, 132.29, 131.15, 129.22, 129.02, 128.98, 128.89, 128.05, 127.00, 126.82, 126.55, 48.90, 21.14. HRMS (ESI, m/z) calcd for C_23_H_20_ClN_2_ [M + H]^+^ 359.1315, found 359.1327.

##### 1-Benzyl-2–(4-chlorophenyl)-4-(*m*-tolyl)-1*H*-imidazole (16k)

White solid, yield 23.00%. ^1^H NMR (500 MHz, Chloroform-*d*) δ 7.66 (s, 1H), 7.48 (s, 1H), 7.36–7.26 (m, 5H), 7.15–7.03 (m, 4H), 7.00–6.93 (m, 3H), 4.96 (s, 2H), 2.28 (s, 3H). ^13^C NMR (126 MHz, Chloroform-*d*) δ 138.95, 137.93, 137.44, 136.41, 134.82, 134.07, 132.29, 129.19, 129.08, 128.89, 128.04, 128.02, 127.47, 127.40, 126.78, 123.63, 48.86, 21.46. HRMS (ESI, m/z) calcd for C_23_H_20_ClN_2_ [M + H]^+^ 359.1315, found 359.1324.

##### 1-Benzyl-2,4-bis(4-chlorophenyl)-1*H*-imidazole (16l)

White solid, yield 13.37%. ^1^H NMR (500 MHz, Chloroform-*d*) δ 7.65 (s, 1H), 7.45–7.37 (m, 2H), 7.36–7.31 (m, 2H), 7.30–7.26 (m, 3H), 7.21–7.14 (m, 2H), 7.13–7.07 (m, 2H), 6.99–6.91 (m, 2H), 4.95 (s, 2H). ^13^C NMR (126 MHz, Chloroform-*d*) δ 137.87, 137.62, 136.23, 135.13, 132.81, 132.30, 132.18, 129.40, 128.94, 128.70, 128.43, 128.13, 127.84, 127.69, 126.82, 48.92. HRMS (ESI, m/z) calcd for C_22_H_16_Cl_2_N_2_ [M + H]^+^ 379.0769, found 379.0783.

##### 1-Benzyl-4–(3-chlorophenyl)-2–(4-chlorophenyl)-1*H*-imidazole (16m)

White solid, yield 56.18%. ^1^H NMR (500 MHz, Chloroform-*d*) δ 7.67 (s, 1H), 7.58–7.57 (m, 1H), 7.37–7.34 (m, 2H), 7.30–7.26 (m, 3H), 7.22–7.20 (m, 1H), 7.13–7.10 (m, 4H), 6.96–6.93 (m, 2H), 4.96 (s, 2H). ^13^C NMR (126 MHz, Chloroform-*d*) δ 137.62, 137.51, 136.09, 136.00, 135.20, 134.27, 132.13, 129.40, 129.37, 128.93, 128.43, 128.14, 128.09, 126.79, 126.69, 126.60, 124.50, 48.93. HRMS (ESI, m/z) calcd for C_22_H_17_Cl_2_N_2_ [M + H]^+^ 379.0769, found 379.0786.

##### 1-Benzyl-2-phenyl-4-(*p*-tolyl)-1*H*-imidazole (16n)

White solid, yield 28.10%. ^1^H NMR (500 MHz, Chloroform-*d*) δ 7.64 (s, 1H), 7.38–7.35 (m, 5H), 7.30–7.26 (m, 3H), 7.25–7.18 (m, 2H), 7.01 (d, *J* = 8.0 Hz, 2H), 6.98–6.94 (m, 2H), 4.96 (s, 2H), 2.27 (s, 3H). ^13^C NMR (126 MHz, Chloroform-*d*) δ 138.20, 136.93, 136.50, 136.05, 131.41, 130.99, 130.52, 128.89, 128.79, 128.68, 128.40, 127.93, 126.98, 126.50, 48.84, 21.13. HRMS (ESI, m/z) calcd for C_23_H_21_N_2_ [M + H]^+^ 325.1705, found 325.1711.

##### 1-Benzyl-4-phenyl-2-(thiophen-2-yl)-1*H*-imidazole (16o)

White solid, yield 35.22%. ^1^H NMR (500 MHz, Chloroform-*d*) δ 7.60 (s, 1H), 7.45–7.40 (m, 3H), 7.27–7.25 (m, 5H), 7.08 (d, *J* = 5.0 Hz, 1H), 6.96–6.94 (m, 2H), 6.86–6.83 (m, 2H), 4.94 (s, 2H). ^13^C NMR (126 MHz, Chloroform-*d*) δ 138.20, 137.00, 136.35, 134.15, 131.14, 129.63, 129.15, 128.95, 128.82, 128.01, 127.78, 127.17, 126.95, 123.32, 122.25, 48.92. HRMS (ESI, m/z) calcd for C_20_H_17_N_2_S [M + H]^+^, 317.1112, found 317.1125.

##### 1-Benzyl-4–(4-chlorophenyl)-2-phenyl-1*H*-imidazole (16p)

White solid, yield 27.83%. ^1^H NMR (500 MHz, Chloroform-*d*) δ 7.67(s, 1H), 7.43–7.33 (m, 5H), 7.29–7.27 (m, 3H), 7.22–7.12 (m, 4H), 6.99–6.93 (m, 2H), 4.96 (s, 2H). ^13^C NMR (126 MHz, Chloroform-*d*) δ 137.16, 137.10, 136.24, 133.97, 132.76, 132.15, 130.84, 130.00, 129.22, 129.04, 128.97, 128.85, 128.27, 128.04, 128.00, 127.33, 126.98, 48.93. HRMS (ESI, m/z) calcd for C_22_H_18_ClN_2_ [M + H]^+^ 345.1159, found 345.1172.

#### Preparation of 2,4-bis(4-chlorophenyl)-1*H*-imidazole (19)

**19** was prepared using a modified procedure previously reported by the Li group^36^. A mixture of 4-chlorobenzene-1-carboximidamide hydrochloride (2.61 g, 20 mmol), THF (36 ml) and H_2_O (9 ml) was stirred and heated to 70 °C. α-Bromo-4-chloroacetophenone (4.20 g, 18 mmol) in THF (11 ml) was slowly added, and the reaction mixture was then stirred at 70 °C for 8 h. THF was removed under vacuum. The resulting mixture was dissolved in water and dichloromethane (30 ml/30 ml), and the slow addition of concentrated HCl led to the precipitation of the product, which was further filtered and washed with dichloromethane to afford **19** as a light yellow solid (1.12 g, 21.6%).

^1^H NMR (500 MHz, DMSO-*d*_6_) δ 12.81 (s, 1H), 8.07–7.99 (m, 2H), 7.91–7.85 (m, 2H), 7.82 (s, 1H), 7.58–7.52 (m, 2H), 7.47–7.42 (m, 2H). ^13^C NMR (126 MHz, DMSO-*d*_6_) δ 144.89, 140.00, 133.33, 132.64, 130.45, 129.18, 128.75, 128.39, 126.51, 125.94, 115.15. LC-MS (ESI, m/z) 288.8 [M + H]^+^.

#### General synthetic procedure for 1-substituted 2,4-bis(4-chlorophenyl)-1H-imidazoles (20)

Excess aliphatic halide and K_2_CO_3_ were added to a solution of 2,4-bis(4-chlorophenyl)-1*H*-imidazole (**19**) in the indicated solvent. The reaction mixture was stirred at the indicated temperature. The reaction mixture was extracted with ethyl acetate and water. The organic layer was collected, washed with water and brine, dried over anhydrous magnesium sulphate, and condensed under a vacuum. The resulting crude product was purified by column chromatography using petroleum ether/ethyl acetate as the eluent.

##### 1-Allyl-2,4-bis(4-chlorophenyl)-1*H*-imidazole (20a)

20a was prepared from 19 (0.58 g, 2 mmol) and allyl bromide (6.92 ml, 20 mmol) in acetonitrile at 80 °C. White solid (79 mg, 11.9%). ^1^H NMR (500 MHz, chloroform-*d*) δ 7.76 (d, *J* = 8.0 Hz, 2H), 7.59 (d, *J* = 8.0 Hz, 2H), 7.44 (d, *J* = 8.0 Hz, 2H), 7.34 (d, *J* = 8.0 Hz, 2H), 7.27 (s, 1H), 6.03–6.01 (m, 1H), 5.35 (d, *J* = 10.5 Hz, 1H), 5.19 (d, *J* = 17.0 Hz, 1H), 4.61 (s, 2H). ^13^C NMR (126 MHz, chloroform-*d*) δ 147.12, 140.43, 135.26, 132.97, 132.48, 132.40, 130.11, 128.91, 128.73, 126.17, 118.33, 117.06, 49.37, 29.71. LC-MS (ESI, m/z) 328.8 [M + H]^+^.

##### 2,4-Bis(4-chlorophenyl)-1-pentyl-1*H*-imidazole (20b)

20b was prepared from 19 (0.29 g, 1 mmol) and 1-pentyl bromide (0.17 g, 1.1 mmol) in acetone at 60 °C. White solid (30 mg, 8.36%). ^1^H NMR (500 MHz, chloroform-*d*) δ 7.75 (d, *J* = 8.0 Hz, 2H), 7.56 (d, *J* = 8.0 Hz, 2H), 7.45 (d, *J* = 8.0 Hz, 2H), 7.33 (d, *J* = 8.5 Hz, 2H), 7.28 (s, 1H), 3.96 (t, *J* = 7.5 Hz, 2H), 1.77 (t, *J* = 7.5 Hz, 2H), 1.30–1.25 (m, 4H), 0.87 (t, *J* = 7.0 Hz, 3H). ^13^C NMR (126 MHz, chloroform-*d*) δ 147.04, 140.20, 135.12, 132.37, 130.32, 128.93, 128.71, 126.11, 116.47, 47.08, 30.80, 28.62, 22.14, 13.84. LC-MS (ESI, m/z) 359.2 [M + H]^+^.

##### Methyl 2–(2,4-bis(4-chlorophenyl)-1*H*-imidazol-1-yl)acetate (20c)

20c was prepared from 19 (0.58 g, 2 mmol) and methyl bromoacetate (0.46 g, 3 mmol) in acetonitrile at 80 °C. White solid (420 mg, 58.2%). ^1^H NMR (500 MHz, chloroform-*d*) δ 7.77–7.74 (m, 2H), 7.54–7.45 (m, 4H), 7.35–7.26 (m, 3H), 4.71 (s, 2H), 3.81 (s, 3H). ^13^C NMR (126 MHz, chloroform-*d*) δ 168.07, 147.72, 140.79, 135.63, 132.64, 132.16, 130.29, 129.12, 128.72, 128.17, 126.28, 117.62, 53.02, 48.22. LC-MS (ESI, m/z) 360.8 [M + H]^+^.

##### 2–(2,4-Bis(4-chlorophenyl)-1*H*-imidazol-1-yl)acetamide (20d)

20d was prepared from 19 (0.58 g, 2 mmol) and bromoacetamide (0.414 g, 3 mmol) in DMF at 80 °C. White solid (120 mg, 17.3%). ^1^H NMR (500 MHz, DMSO-*d*_6_) δ 7.83–7.79 (m, 3H), 7.71 (s, 1H), 7.65 (d, *J* = 8.5 Hz, 2H), 7.57 (d, *J* = 8.5 Hz, 2H), 7.43 (d, *J* = 8.5 Hz, 2H), 7.39 (s, 1H), 4.71 (s, 2H). ^13^C NMR (126 MHz, DMSO-*d*_6_) δ 168.52, 146.37, 138.18, 133.45, 133.02, 130.56, 129.92, 129.07, 128.55, 128.46, 125.79, 120.41, 49.05. LC-MS (ESI, m/z) 345.9 [M + H]^+^.

##### 3–(2,4-Bis(4-chlorophenyl)-1*H*-imidazol-1-yl)propan-1-ol (20e)

20e was prepared from 19 (0.29 g, 1 mmol) and 3-bromo-1-propanol (132 μL, 1.1 mmol) in acetonitrile at 80 °C. White solid (143 mg, 41.2%). ^1^H NMR (500 MHz, chloroform-*d*) δ 7.76–7.73 (m, 2H), 7.60–7.57 (m, 2H), 7.46–7.43 (m, 2H), 7.35–7.30 (m, 3H), 7.27 (s, 1H), 4.16 (t, *J* = 7.0 Hz, 2H), 3.62 (t, *J* = 5.5 Hz, 2H), 1.97 (t, *J* = 6.0 Hz, 2H). ^13^C NMR (126 MHz, chloroform-*d*) δ 147.12, 140.29, 135.25, 132.50, 132.35, 130.28, 129.00, 128.88, 128.76, 126.15, 116.84, 58.82, 43.68, 33.30. LC-MS (ESI, m/z) 347.3 [M + H]^+^.

#### General synthetic procedure for 2,4-bis(benzyloxy)-5-arylpyrimidines (22)

2,4-Bis(benzyloxy)-5-bromopyrimidine **21** is commercially available and was used without further purification. **22** was prepared using a procedure similar to that for **14**. To a solution of **21** in 1,4-dioxane (9 ml), aryl boronic acid (2.5 equiv.), Pd(dppf)Cl_2_ (5 mol%) and aqueous Na_2_CO_3_ solution (3.3 M, 2.0 equiv.) was added. The reaction mixture was heated to reflux and stirred under a nitrogen atmosphere for 4 h. After cooling to room temperature, the reaction mixture was extracted with water and ethyl acetate. The organic layer was collected, washed with water and brine, dried over anhydrous magnesium sulphate, and condensed under a vacuum. The resulting crude product was purified by column chromatography using petroleum ether/ethyl acetate as the eluent.

##### 2,4-Bis(benzyloxy)-5-phenylpyrimidine (22a)

White solid, yield 38.55%. ^1^H NMR (500 MHz, Chloroform-*d*) δ 8.31 (s, 1H), 7.53–7.50 (m, 4H), 7.44–7.27 (m, 11H), 5.50 (s, 2H), 5.46 (s, 2H). ^13^C NMR (126 MHz, Chloroform-*d*) δ 167.45, 163.83, 158.11, 136.62, 136.23, 133.22, 128.81, 128.49, 128.47, 128.43, 128.08, 128.02, 127.95, 127.64, 127.54, 116.33, 69.23, 68.34. HRMS (ESI, m/z) calcd for C_24_H_21_N_2_O_2_ [M + H]^+^ 369.1603, found 369.1615.

##### 2,4-Bis(benzyloxy)-5-(*o*-tolyl)pyrimidine (22b)

White solid, yield 42.02%. ^1^H NMR (500 MHz, Chloroform-*d*) δ 8.13 (s, 1H), 7.51 (d, *J* = 7.5 Hz, 2H), 7.39 (t, *J* = 8.0 Hz, 2H), 7.36–7.20 (m, 9H), 7.15 (d, *J* = 7.5 Hz, 1H), 5.46 (s, 2H), 5.45 (s, 2H), 2.14 (s, 3H). ^13^C NMR (126 MHz, Chloroform-*d*) δ 167.74, 164.14, 158.66, 137.33, 136.64, 136.26, 132.99, 130.41, 130.05, 128.50, 128.43, 128.20, 128.15, 128.06, 127.92, 127.60, 125.76, 116.56, 69.26, 68.13, 20.02. HRMS (ESI, m/z) calcd for C_25_H_22_N_2_O_2_ [M + H]^+^ 383.1760, found 383.1773.

##### 2,4-Bis(benzyloxy)-5-(*m*-tolyl)pyrimidine (22c)

White solid, yield 39.02%. ^1^H NMR (500 MHz, Chloroform-*d*) δ 8.31 (s, 1H), 7.50 (d, *J* = 7.5 Hz, 2H), 7.41–7.28 (m, 11H), 7.16 (d, *J* = 7.0 Hz, 1H), 5.49 (s, 2H), 5.46 (s, 2H), 2.38 (s, 3H). ^13^C NMR (126 MHz, Chloroform-*d*) δ 167.48, 163.79, 158.04, 138.03, 136.67, 136.32, 133.12, 129.59, 128.49, 128.35, 128.11, 128.04, 127.94, 127.52, 125.92, 116.43, 69.23, 68.32, 21.49. HRMS (ESI, m/z) calcd for C_25_H_23_N_2_O_2_ [M + H]^+^ 383.1760; found 383.1771.

##### 2,4-Bis(benzyloxy)-5-(*p*-tolyl)pyrimidine (22d)

White solid, yield 43.70%. ^1^H NMR (500 MHz, Chloroform-*d*) δ 8.30 (s, 1H), 7.49 (d, *J* = 7.5 Hz, 2H), 7.44–7.28 (m, 10H), 7.27–7.19 (m, 2H), 5.49 (s, 2H), 5.45 (s, 2H), 2.38 (s, 3H). ^13^C NMR (126 MHz, Chloroform-*d*) δ 167.44, 163.66, 157.90, 137.48, 136.67, 136.30, 129.17, 128.64, 128.48, 128.46, 128.06, 127.57, 116.28, 69.18, 68.31, 21.20. HRMS (ESI, m/z) calcd for C_25_H_23_N_2_O_2_ [M + H]^+^ 383.1760, found 383.1771.

##### 2,4-Bis(benzyloxy)-5–(4-fluorophenyl)pyrimidine (22e)

White solid, yield 34.33%. ^1^H NMR (500 MHz, Chloroform-*d*) δ 8.28 (s, 1H), 7.49–7.42 (m, 4H), 7.36 (m, 10H), 7.12–7.07 (m, 2H), 5.49 (s, 2H), 5.46 (s, 2H). ^13^C NMR (126 MHz, Chloroform-*d*) δ 167.37, 163.87, 163.35, 161.39, 157.93, 136.57, 136.09, 130.49, 128.55, 128.48, 128.07, 128.06, 127.58, 115.52, 115.44, 115.35, 69.27, 68.45. HRMS (ESI, m/z) calcd for C_24_H_20_FN_2_O_2_ [M + H]^+^ 387.1509, found 387.1519.

##### 2,4-Bis(benzyloxy)-5–(4-chlorophenyl)pyrimidine (22f)

White solid, yield 18.47%. ^1^H NMR (500 MHz, Chloroform-*d*) δ 8.29 (s, 1H), 7.49 (d, *J* = 7.5 Hz, 2H), 7.45 (d, *J* = 7.5 Hz, 2H), 7.41–7.25 (m, 10H), 5.48 (s, 2H), 5.46 (s, 2H). ^13^C NMR (126 MHz, Chloroform-*d*) δ 167.34, 163.99, 157.98, 136.51, 136.00, 133.66, 131.67, 130.03, 128.65, 128.57, 128.49, 128.10, 128.07, 127.62, 115.23, 69.31, 68.52. HRMS (ESI, m/z) calcd for C_24_H_20_ClN_2_O_2_ [M + H]^+^ 403.1213, found 403.1230.

##### 2,4-Bis(benzyloxy)-5–(2-chlorophenyl)pyrimidine (22g)

A colourless liquid, yield 23.55%. ^1^H NMR (500 MHz, Chloroform-*d*) δ 8.18 (s, 1H), 7.51–7.46 (m, 2H), 7.46–7.39 (m, 1H), 7.38–7.32 (m, 2H), 7.32–7.19 (m, 9H), 5.45 (s, 2H), 5.43 (s, 2H). ^13^C NMR (126 MHz, Chloroform-*d*) δ 167.82, 164.56, 158.96, 136.64, 136.24, 134.40, 132.59, 131.94, 129.74, 129.55, 128.59, 128.49, 128.25, 128.17, 128.00, 127.68, 114.66, 69.45, 68.46. HRMS (ESI, m/z) calcd for C_24_H_20_ClN_2_O_2_ [M + H]^+^ 403.1213, found 403.1227.

##### 2,4-Bis(benzyloxy)-5–(3-chlorophenyl)pyrimidine (22h)

White solid, yield 20.73%. ^1^H NMR (500 MHz, Chloroform-*d*) δ 8.31 (s, 1H), 7.54 (s, 1H), 7.52–7.47 (m, 2H), 7.41–7.30 (m, 11H), 5.50 (s, 2H), 5.47 (s, 2H). ^13^C NMR (126 MHz, Chloroform-*d*) δ 167.36, 164.12, 158.13, 136.49, 135.99, 135.02, 134.29, 129.67, 128.90, 128.57, 128.50, 128.10, 127.71, 127.55, 126.86, 115.08, 69.36, 68.51. HRMS (ESI, m/z) calcd for C_24_H_20_ClN_2_O_2_ [M + H]^+^ 403.1213, found 403.1226.

##### 2,4-Bis(benzyloxy)-5–(2,4-dichlorophenyl)pyrimidine (22i)

White solid, yield 9.56%. ^1^H NMR (500 MHz, Chloroform-*d*) δ 8.17 (s, 1H), 7.53–7.47 (m, 3H), 7.41–7.35 (m, 2H), 7.34–7.25 (m, 7H), 7.34–7.25 (m, 7H), 7.21 (d, *J* = 8.0 Hz, 1H), 5.46 (s, 2H), 5.44 (s, 2H). ^13^C NMR (126 MHz, Chloroform-*d*) δ 167.64, 164.58, 158.86, 136.41, 135.93, 135.08, 134.65, 132.55, 131.08, 129.57, 128.50, 128.45, 128.16, 128.11, 128.03, 127.68, 127.11, 113.49, 69.42, 68.51. HRMS (ESI, m/z) calcd for C_24_H_19_Cl_2_N_2_O_2_ [M + H]^+^ 437.0824, found 437.0842.

##### (4–(2,4-bis(benzyloxy)pyrimidin-5-yl)phenyl)methanol (22j)

White solid, yield 44.04%. ^1^H NMR (500 MHz, Chloroform-*d*) δ 8.28 (s, 1H), 7.53–7.47 (m, 4H), 7.43–7.27 (m, 10H), 5.49 (s, 2H), 5.46 (s, 2H), 4.72 (d, *J* = 3.5 Hz, 2H), 1.84 (s, 1H). ^13^C NMR (126 MHz, DMSO-*d_6_*) δ 162.72, 159.08, 153.30, 135.64, 131.86, 131.42, 127.80, 124.21, 123.79, 123.75, 123.28, 122.88, 122.34, 111.27, 64.52, 63.70, 60.29. HRMS (ESI, m/z) calcd for C_25_H_23_N_2_O_3_ [M + H]^+^ 399.1709, found 399.1722.

##### 4–(2,4-Bis(benzyloxy)pyrimidin-5-yl)phenol (22k)

White solid, yield 22.00%. ^1^H NMR (500 MHz, DMSO-*d_6_*) δ 9.56 (s, 1H), 8.34 (s, 1H), 7.47–7.30 (m, 12H), 6.83–6.78 (m, 2H), 5.46 (s, 2H), 5.41 (s, 2H). ^13^C NMR (126 MHz, DMSO-*d_6_*) δ 166.68, 162.71, 157.36, 156.97, 136.64, 136.25, 129.77, 128.35, 128.33, 127.91, 127.86, 127.82, 127.58, 126.89, 123.09, 115.60, 115.46, 115.14, 68.33, 67.73. HRMS (ESI, m/z) calcd for C_24_H_21_N_2_O_3_ [M + H]^+^ 385.1552, found 385.1564.

##### 3–(2,4-Bis(benzyloxy)pyrimidin-5-yl)phenol (22l)

White solid, yield 18.40%. ^1^H NMR (500 MHz, Chloroform-*d*) δ 8.83 (s, 1H), 8.23 (s, 1H), 7.40–7.36 (m, 2H), 7.38–7.10 (m, 9H), 7.08 (s, 1H), 7.00 (d, *J* = 7.5 Hz, 1H), 6.89 (dd, *J* = 8.0, 2.5 Hz, 1H), 5.39 (s, 2H), 5.37 (s, 2H). ^13^C NMR (126 MHz, Chloroform-*d*) δ 167.58, 163.54, 157.63, 156.90, 136.39, 136.13, 134.14, 129.73, 128.62, 128.59, 128.15, 128.10, 127.86, 127.54, 120.65, 116.39, 116.15, 115.47, 69.48, 68.65. HRMS (ESI, m/z) calcd for C_24_H_21_N_2_O_3_ [M + H]^+^ 385.1552, found, 385.1562.

##### 2–(2,4-Bis(benzyloxy)pyrimidin-5-yl)phenol (22m)

White solid, yield 12.53%. ^1^H NMR (500 MHz, Chloroform-d) δ 8.30 (s, 1H), 7.52–7.47 (m, 2H), 7.40–7.27 (m, 9H), 7.19 (dd, *J* = 7.5, 1.5 Hz, 1H), 7.05–6.96 (m, 2H), 5.52 (s, 2H), 5.47 (s, 2H). ^13^C NMR (126 MHz, Chloroform-*d*) δ 166.97, 164.29, 160.23, 153.74, 136.38, 135.48, 131.04, 129.92, 128.66, 128.52, 128.36, 128.13, 128.07, 127.91, 121.19, 120.66, 117.38, 112.81, 69.45, 68.87. HRMS (ESI, m/z) calcd for C_24_H_20_N_2_O_3_ [M + H]^+^ 385.1552, found 385.1563.

##### 4–(2,4-Bis(benzyloxy)pyrimidin-5-yl)benzoic acid (22n)

White solid, yield 20.04%. ^1^H NMR (500 MHz, DMSO-*d_6_*) δ 12.97 (s, 1H), 8.51 (s, 1H), 7.97 (d, *J* = 8.5 Hz, 2H), 7.72 (d, *J* = 8.5 Hz, 2H), 7.51–7.46 (m, 2H), 7.46–7.29 (m, 8H), 5.49 (s, 2H), 5.46 (s, 2H). ^13^C NMR (126 MHz, DMSO-*d_6_*) δ 166.90, 166.82, 163.60, 158.51, 137.23, 136.44, 135.99, 129.20, 128.70, 128.39, 128.36, 127.98, 127.68, 114.59, 68.59, 68.07. HRMS (ESI, m/z) calcd for C_25_H_20_N_2_O_4_ [M + H]^+^ 413.1501, found 413.1518.

##### 2,4-Bis(benzyloxy)-5-(thiophen-2-yl)pyrimidine (22o)

Brownish solid, yield 15.86%. ^1^H NMR (500 MHz, Chloroform-*d*) δ 8.56 (s, 1H), 7.48 (t, *J* = 7.0 Hz, 4H), 7.43–7.30 (m, 8H), 7.09–7.04 (m, 1H), 5.55 (s, 2H), 5.46 (s, 2H). ^13^C NMR (126 MHz, Chloroform-*d*) δ 166.07, 163.33, 156.38, 136.54, 135.89, 134.48, 128.59, 128.51, 128.22, 128.10, 128.03, 127.30, 125.46, 125.18, 110.61, 69.37, 69.02. HRMS (ESI, m/z) calcd for C_22_H_19_N_2_O_2_S [M + H]^+^ 375.1167, found 375.1181.

##### 2,4-Bis(benzyloxy)-5-(furan-2-yl)pyrimidine (22p)

White solid, yield 47.78%. ^1^H NMR (500 MHz, Chloroform-*d*) δ 8.75 (s, 1H), 7.51–7.29 (m, 11H), 6.74 (d, *J* = 2.5 Hz, 1H), 6.43 (d, *J* = 1.0 Hz, 1H), 5.54 (s, 2H), 5.46 (s, 2H). ^13^C NMR (126 MHz, Chloroform-*d*) δ 165.51, 162.97, 154.50, 146.54, 141.70, 136.55, 135.93, 128.69, 128.48, 128.34, 128.12, 128.07, 111.68, 109.53, 107.59, 69.34, 68.99. HRMS (ESI, m/z) calcd for C_22_H_18_N_2_O_3_ [M + H]^+^ 359.1396, found 359.1409.

### Biological evaluation

#### Cell culture

Three breast cancer cell lines, MCF-7, MDA-MB-231, and 4T1 were used in this study. The cells were purchased from the Institute of Basic Medicine, Chinese Academy of Medical Sciences (Beijing, China). MCF-7 and MDA-MB-23 cells were cultured in DMEM, and 4T1 cells were cultured in RPMI-1640 using 10% foetal bovine serum (FBS) and 1% penicillin-streptomycin solution (PS) in humidified air containing 5% CO_2_ at 37 °C.

#### Antiproliferative activity assay

The antiproliferative activity of the target compounds against the breast cancer cell lines MCF-7, MDA-MB-231, and 4T1 was determined using Cell Counting Kit-8 (Beyotime, Shanghai, China). The target compounds were dissolved in DMSO, diluted with cell culture medium to the desired concentration, and stored at −20 °C before use. Cells were seeded into a 96-well plate at a density of 7000 cells/well (200 μL/well) and incubated overnight. Compound solutions at different concentrations were added and incubated for 48 h. CCK8 solution was added and further incubated for 1–2 h in the dark according to the manufacturer’s instructions. Absorbance was measured at a wavelength of 450 nm using Synergy H1 (BioTek Instruments, Inc., Winooski, VT, USA). IC_50_ values were calculated by GraphPad Prism 8 (GraphPad Software, Inc., San Diego, CA, USA). The experiments were performed in triplicate.

#### Western blotting assay

Primary antibodies against HSP70, HSP90, and GAPDH were purchased from Abcam (Cambridge, MA, USA). Primary antibodies against ERK and AKT were purchased from Beyotime (Shanghai, China). HRP (horseradish peroxidase)-labelled anti-rabbit immunoglobulin G (H + L) secondary antibody was purchased from Abbkine (Redlands, CA, USA). MCF-7 cells were incubated in a 6-well plate at a density of 10^6^ cells/well (4 ml/well) at 37 °C for 12 h. The cells were then treated with the compounds and 17-AAG at different concentrations at 37 °C for 24 h. The 6-well plate was placed on ice for 15 min. Cells were collected into a centrifuge tube and lysed by radioimmunoprecipitation (RIPA) lysis solution containing 1% phenylmethanesulphonyl fluoride (PMSF). The lysate was centrifuged at a speed of 14,000 r/min in a cryogenic high-speed centrifuge for 10 min. The cell supernatant was collected to determine the protein content. Cell lysates were mixed with SDS loading buffer and boiled for 20 min. Equal amounts of protein in cell lysates were separated by 10% sodium dodecyl sulphate–polyacrylamide gel electrophoresis (SDS–PAGE), which was prepared using the PAGE Gel Fast Preparation Kit (Epizyme, PG112, China). The protein on the separating gel was transferred to a polyvinylidene fluoride (PVDF) membrane. The membrane was blocked in skim milk for 80 min and incubated with primary antibody (1:4000) at 4 °C overnight. After further incubation with HRP anti-rabbit IgG (H + L) (1:5000) at room temperature for 1 h, the protein bands were detected with a gel imager (ProteinSimple, San Jose, CA, USA). The density of proteins was determined using AlphaView SA (Alpha Innotech Corp, version 3.4.0.0, San Leandro, CA, USA). The experiments were performed in triplicate.

#### Fluorescence polarisation (FP) assay

The binding affinity for HSP90α was evaluated using a commercially available Hsp90α N-terminal domain assay kit (Catalogue: #50293, BPS Bioscience, CA, USA). According to the manufacturer’s instructions, 5x HSP90 assay buffer (5 μL), 40 mM dithiothreitol (5 μL), 2 mg/mL BSA (5 μL), and H_2_O (40 μL) were sequentially added to each well. After the addition of diluted FITC-labelled geldanamycin solution (100 nM, 5 μL), compounds at different concentrations (10 μM, 1.0 μM, 0.1 μM, 0.01 μM) were added. The reaction was initiated by the addition of 20 μL of Hsp90α (17 ng/μL). After 2 h of incubation with slow shaking at room temperature, the fluorescent polarisation was measured using Synergy H1 (BioTek Instruments, Inc.). The excitation and emission wavelengths were 485 nm and 530 nm, respectively. IC_50_ values were calculated based on the signal changes in the FP competition assay. The experiments were performed in triplicate.

### Molecular docking

The crystal structure of the human HSP90α N-terminus was downloaded from the Protein Data Bank (PDB ID: 1YET) ^37^. Molecular docking was performed using BIOVIA Discovery Studio 2016 (Dassault Systèmes, San Diego, USA). The protein structure was prepared using the “Prepare Protein” procedure before docking. In this procedure, multiple tasks, including building loops, protonation at pH 7.4, and removing water molecules, were performed. Meanwhile, ligands were prepared by the “Prepare Ligand” procedure. The CHARMM force field was used in the preparation of protein and ligand structures. The binding site was defined using the module “Define site-From PDB Site Records.” The coordinates (in XYZ) of the docking region centre were as follows: *x* = 40.72, *y* = −45.98, *z* = 64.47, and the radius of the active site sphere was 10.3. Docking simulations were performed using CDOCKER, a semiflexible docking program. All other docking parameters were set as default. The protein-ligand docking pose with the highest -CDOCKER_INTERACTION_ENERGY score was taken from the docking results and described.

### Statistical analysis

All values obtained from the experiments are expressed as the means ± SDs. The IC_50_ values at which the concentration of inhibitor reduces cell viability or enzyme activity by 50% were evaluated by nonlinear regression using GraphPad Prism 8.0 software (GraphPad Software, Inc., La Jolla, USA).

## Results and discussions

### Chemical synthesis

#### Synthesis of 1-benzyl-2,4-diarylimidazoles 16

**Scheme 1.**
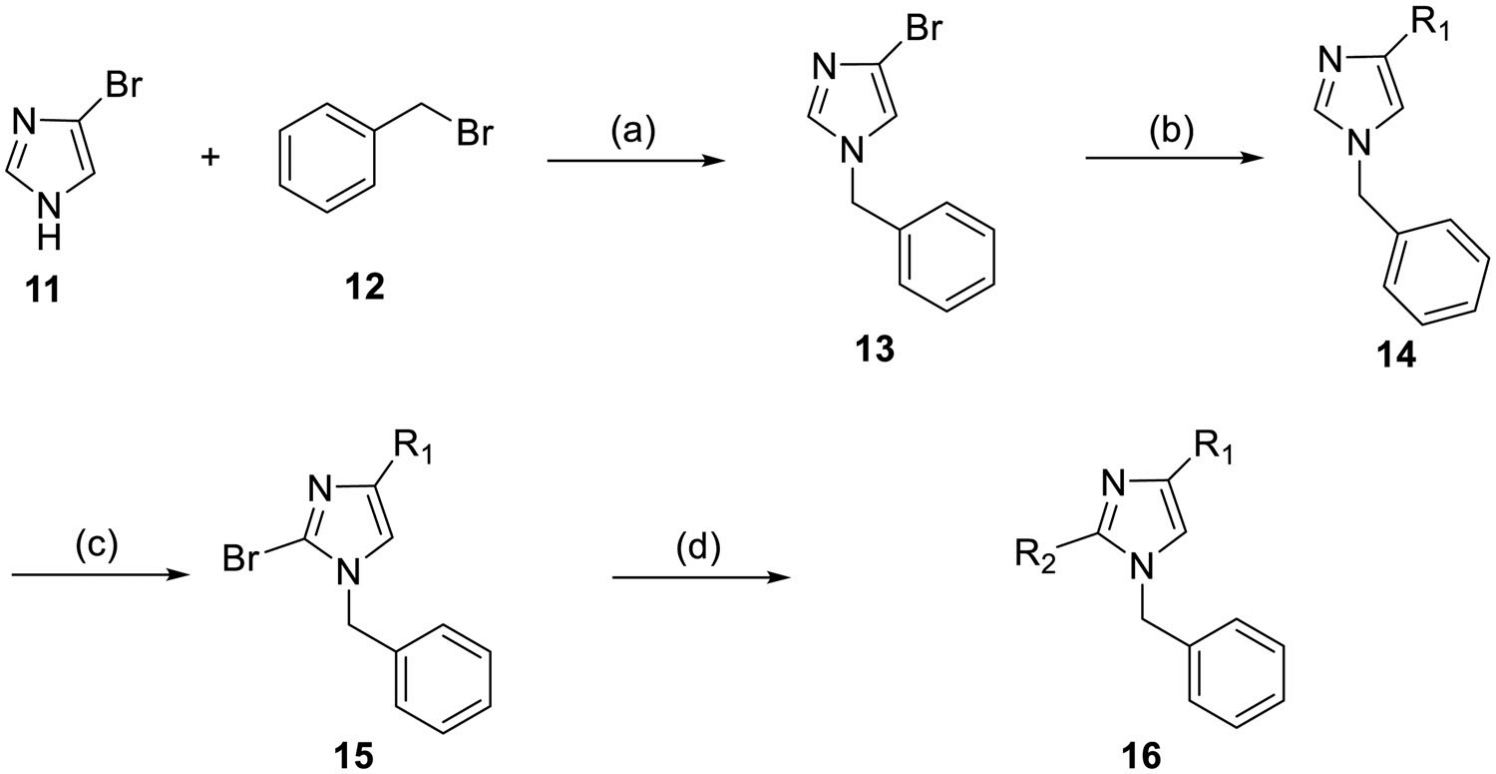
Synthetic route for 1-benzyl-2, 4-diarylimidazoles **16.** Reagents and conditions: (a) 4-bromo-1*H*-imidazole, benzyl bromide, K_2_CO_3_, acetone, r.t., 22 h; (b) Pd(dppf)Cl_2_, Na_2_CO_3_, 1,4-dioxane/H_2_O, N_2_, reflux, 4 h; (c) NBS, CCl_4_, 35 °C, 14 h; (d) Pd(dppf)Cl_2_, Na_2_CO_3_, 1,4-dioxane/H_2_O, N_2_, reflux, 4 h.

1-Benzyl-2,4-diarylimidazoles **16** were synthesised via a four-step synthetic route (Scheme 1). The benzylation of 4-bromoimidazole **11** with benzyl bromide **12** in the presence of K_2_CO_3_ provided 1-benzyl-3-bromoimidazole **13**, which further coupled with aryl boronic acid to afford **14** through a Pd(dppf)Cl_2_-catalysed Suzuki cross-coupling reaction. The bromination of the imidazole ring of **14** with NBS in CCl_4_ gave **15**, which was then converted into the final product **16** through a second Suzuki cross-coupling reaction. The final reaction step always gave low yields, which could be attributed to the debromination of **15**. Simple condition screening experiments revealed that Pd(dppf)Cl_2_ was more effective than other palladium catalysts, such as Pd(PPh_3_)_4_ and Pd(PPh_3_)_2_Cl_2_, at this step.

#### Synthesis of bis(4-chlorophenyl)imidazoles 20

**Scheme 2.**
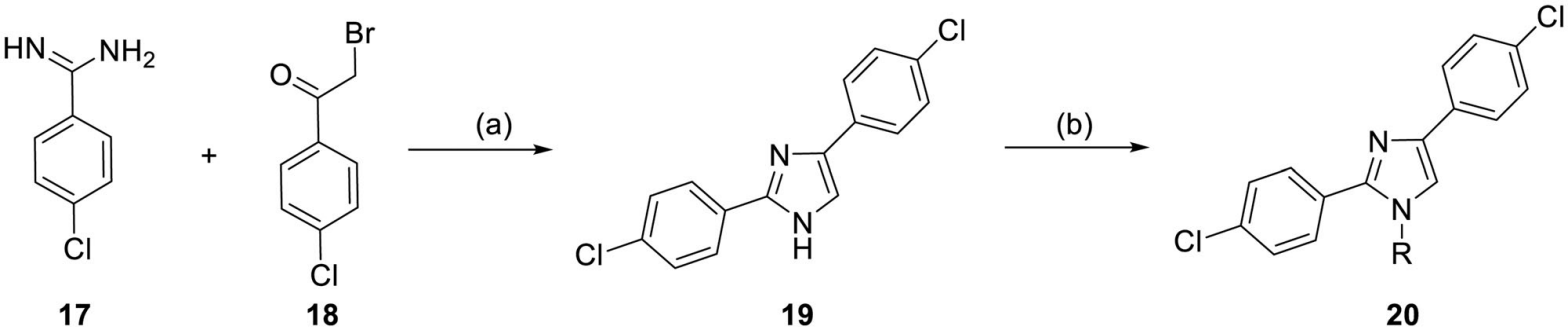
Synthetic route for 2,4-bis(4-chlorophenyl) imidazoles **20**. Reagents and conditions: (a) NaHCO_3_, THF/H_2_O, 70 °C, 5 h; (b) alkyl halides, K_2_CO_3_.

To further investigate the effect of substituents at the N1 position of the imidazole ring, bis(4-chlorophenyl)imidazoles **20** bearing different functional groups at the N1 position were synthesised (Scheme 2). **19** was prepared by a single-step cyclisation reaction from 4-chlorobenzene-1-carboximidamide hydrochloride **17** and 2-chloro-1–(4-chlorophenyl)ethan-1-one **18** in the presence of NaHCO_3_ in THF/H_2_O. Treating **19** with different alkyl halides in the presence of K_2_CO_3_ afforded the *N*-alkylated product **20**.

#### Synthesis of 2,4-bis(benzyloxy)-5-arylpyrimidines

**Scheme 3.**
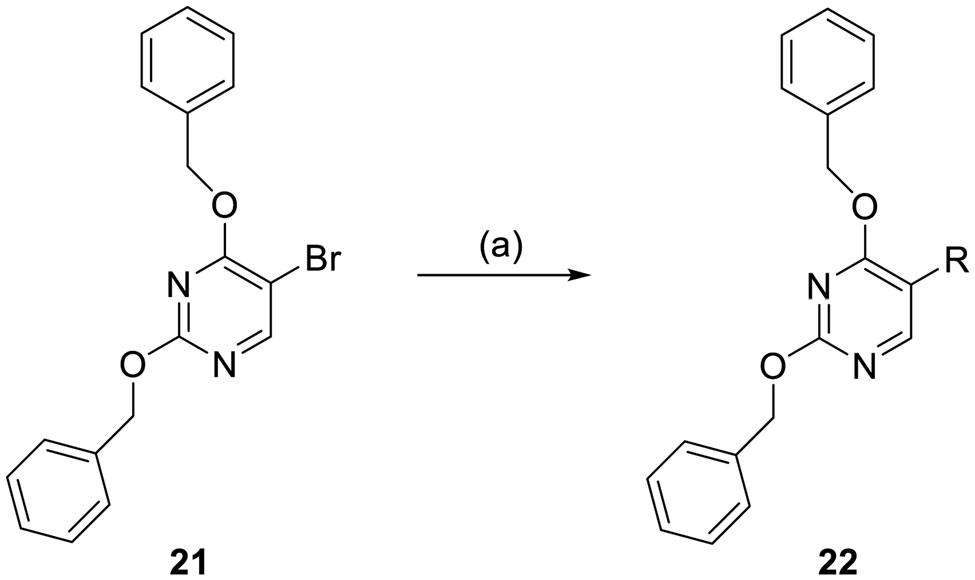
Synthetic route for 2, 4-bis(benzyloxy)-5-arylpyrimidines **22.** Reagents and conditions: (a) Pd(dppf)Cl_2_, Na_2_CO_3_, 1,4-dioxane/H_2_O, N_2_, reflux, 4 h.

2,4-Bis(benzyloxy)-5-arylpyrimidines **22** were obtained through a Pd(dppf)Cl_2-_catalysed Suzuki cross-coupling reaction between 2,4-bis(benzyloxy)-5-bromopyrimidine **21** and a variety of (hetero)aryl boronic acids in the presence of Na_2_CO_3_ in 1,4-dioxane/H_2_O (Scheme 3).

### Biological evaluation

#### Antiproliferative activity evaluation

Female breast cancer is the most commonly diagnosed cancer, with an estimated 2.3 million new cases, and the fifth leading cause of cancer mortality, with an estimated 685,000 new deaths around the world in 2020^38^. HSP90 inhibitors have been extensively studied in clinical trials for the treatment of breast cancer^39^. Although none has been approved in clinical practice, HSP90 inhibitors have shown great promise in breast cancer therapy. In this study, the antiproliferative activity of the synthesised compounds was evaluated against human breast cancer cell lines MCF-7 and MDA-MB-231 and mouse breast cancer cell line 4T1 using a CCK-8 assay kit. As shown in Table 1, it is remarkable that 1-benzyl-2,4-diphenyl imidazole (**16a**) bearing no substituents on any phenyl ring showed antiproliferative activity against all three cancer cell lines. The effect of substituent R_2_ at the C2 position of the imidazole ring was subsequently studied. The methyl group at the *meta*-position of the phenyl ring slightly increased the activity; however, a chlorine atom led to the loss of activity. In the case of the *para*-position, functional groups such as methyl, chloro, and acetyl could preserve the activity, while the hydroxyl group was ineffective. Replacing the phenyl ring with heteroaromatic rings, such as furan and thiophene rings, decreased the activity. The subsequent investigation of substituent R_1_ at the C4 position of the imidazole ring revealed that chloro substitution on the phenyl ring was beneficial for increasing activity. Among these 1-benzyl-2,4-diarylimidazoles, **16j**, **16l**, and **16m** exhibited strong antiproliferative activity with IC_50_ values in the low micromolar range against three breast cancer cell lines. **8a**, the most potent compound among the imidazolidine-based HSP90 inhibitors, was evaluated for comparison. It exhibited weak antiproliferative activity against MCF-7 and 4T1 cells with IC_50_ values of 31.25 ± 0.31 μM and 42.10 ± 1.10 μM, respectively, and it was not cytotoxic towards MDA-MB-231 cells. The above results supported our hypothesis that replacing the imidazolidine ring in **8** with a stable aromatic ring could improve the anticancer activity.

**Table 1.** Antiproliferative activity of 1-benzyl-2, 4-diaryl imidazoles **16**. 
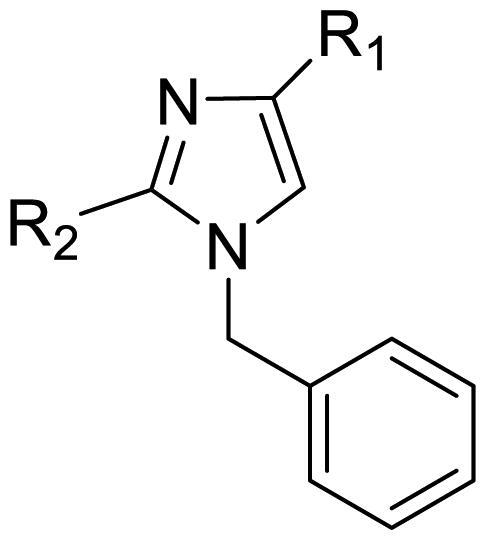

			IC_50_ (μM)		
Comp.	R_1_	R_2_	MCF-7	MDA-MB-231	4T1
**16a**	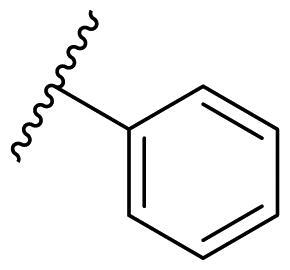	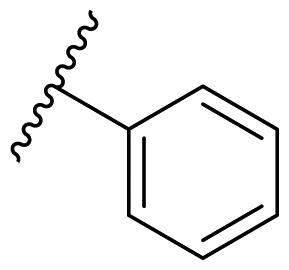	19.89 ± 2.76	20.31 ± 2.16	23.40 ± 0.28
**16b**	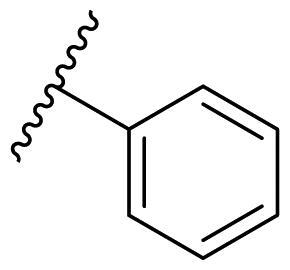	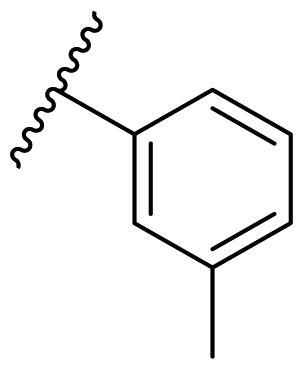	14.56 ± 0.25	16.68 ± 0.80	10.54 ± 0.05
**16c**	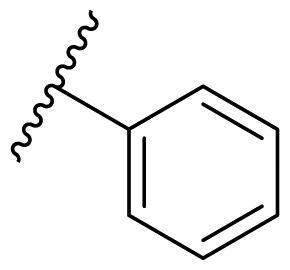	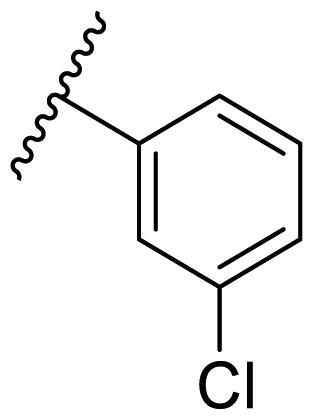	>50	>50	>50
**16d**	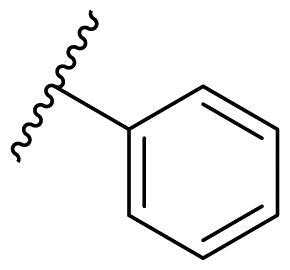	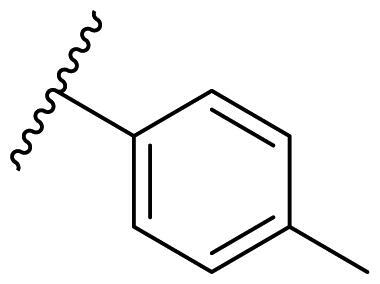	15.29 ± 0.65	20.21 ± 0.67	11.69 ± 1.02
**16e**	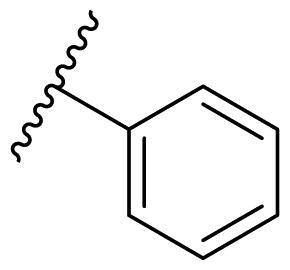	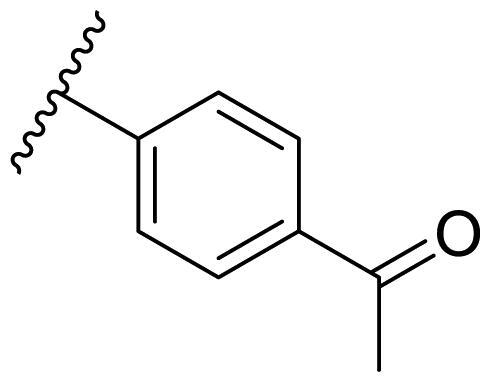	14.56 ± 0.92	23.77 ± 0.23	9.87 ± 0.79
**16f**	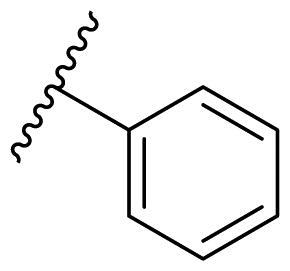	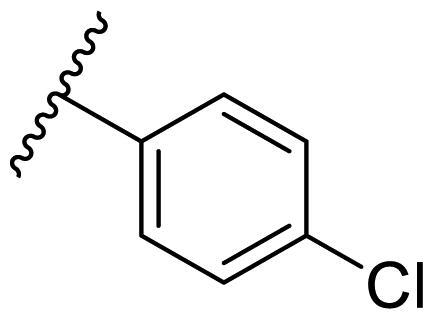	14.27 ± 0.43	21.41 ± 0.96	9.48 ± 0.35
**16g**	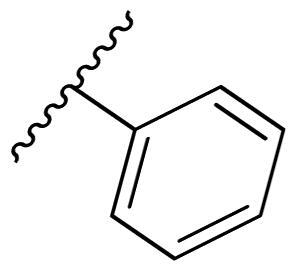	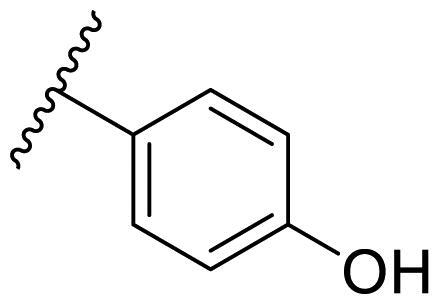	>50	>50	>50
**16h**	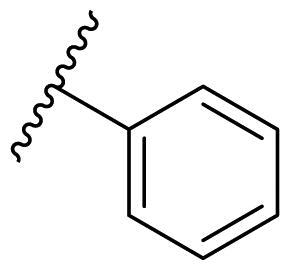	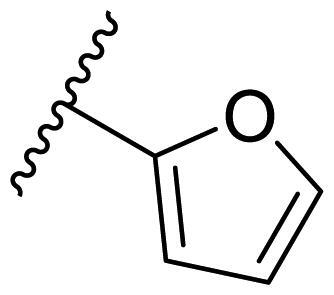	>50	>50	>50
**16i**	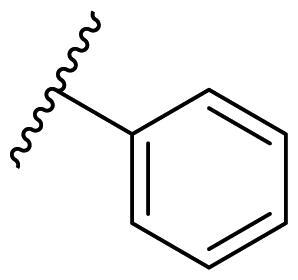	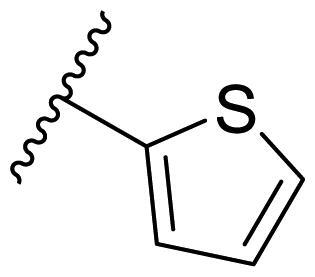	>50	29.83 ± 2.06	30.93 ± 3.35
**16j**	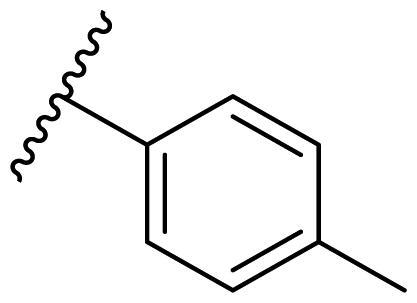	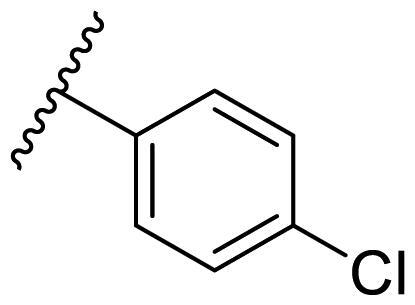	8.13 ± 0.93	14.12 ± 0.11	10.40 ± 0.84
**16k**	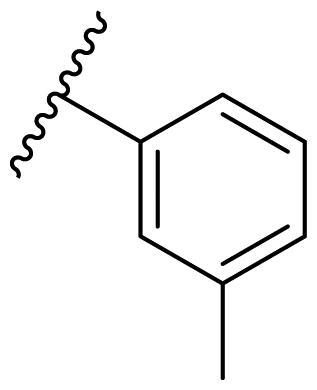	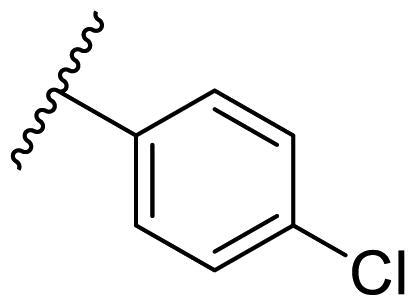	13.95 ± 0.99	14.18 ± 0.86	11.68 ± 0.37
**16l**	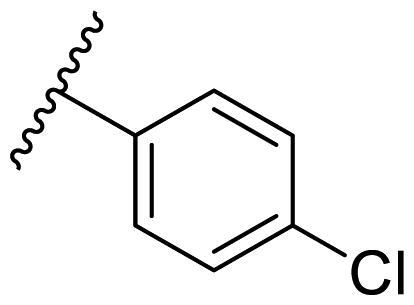	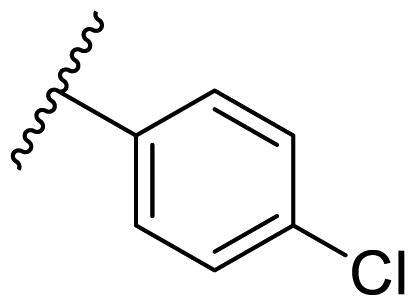	6.16 ± 0.16	7.27 ± 0.16	9.41 ± 0.67
**16m**	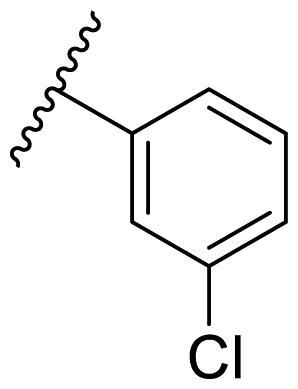	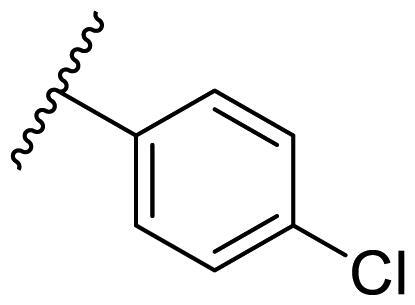	6.07 ± 0.12	7.80 ± 0.32	10.21 ± 0.80
**16n**	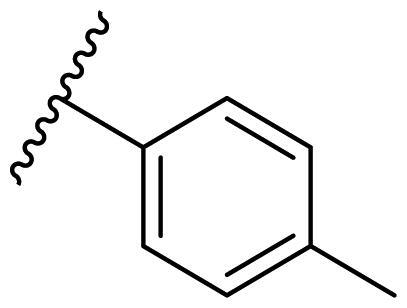	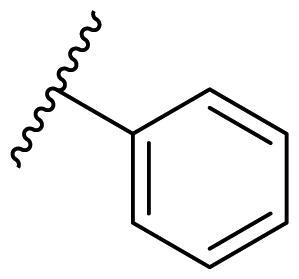	19.83 ± 0.60	21.20 ± 3.60	10.72 ± 0.99
**16o**	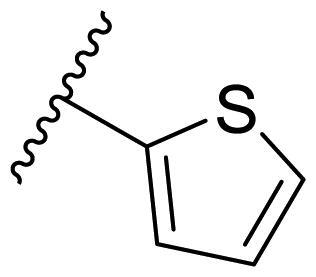	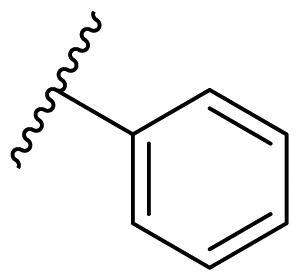	14.75 ± 0.64	21.15 ± 3.10	22.97 ± 1.70
**16p**	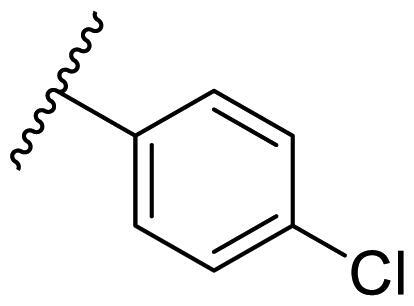	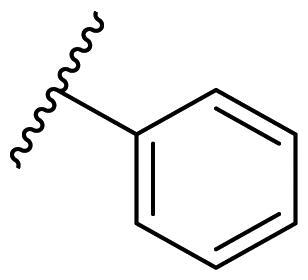	19.29 ± 0.76	17.24 ± 1.20	22.29 ± 3.20
**8a** ^[a]^	--	--	31.25 ± 0.31	>50	42.10 ± 1.10
**17-AAG**	--	--	10.60 ± 0.65	39.07 ± 1.30	0.45 ± 0.09

^[a]^ **8a**: 1,3-dibenzyl-2–(5-nitrothiophen-2-yl)imidazolidine.

The effect of the substituent at the N1 position of the imidazole ring was further studied. The benzyl group proved to be the best substituent at the N1 position. As shown in Table 2, the antiproliferative activity decreased after replacing the benzyl group with other functional groups, such as allyl, aliphatic chain, ester, amide, and aliphatic alcohol. This result indicated that the benzyl group may play essential roles in occupying the space and forming hydrophobic interactions with amino acid residues in the binding site of the HSP90 N-terminus.

**Table 2.** Antiproliferative activity of bis(4-chlorophenyl)imidazoles **20**. 
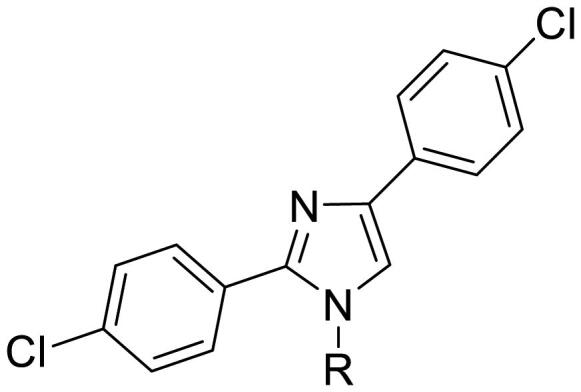

Cmpd.	R	IC_50_ (μM)		
		MCF-7	MDA-MB-231	4T1
**20a**	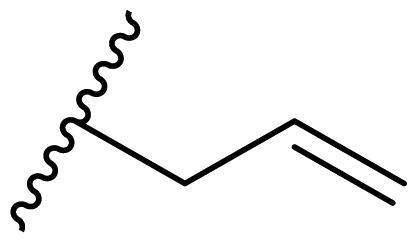	23.14 ± 3.14	20.79 ± 1.47	22.36 ± 1.99
**20b**	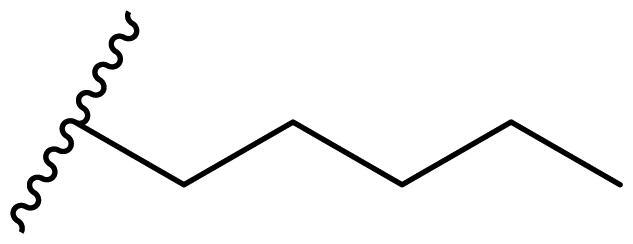	>50	>50	>50
**20c**	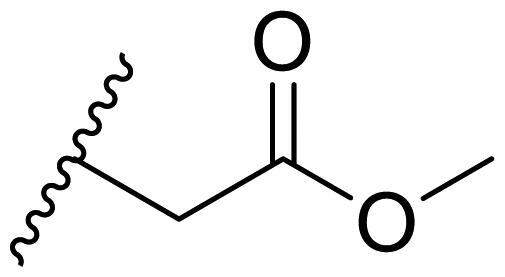	>50	>50	>50
**20d**	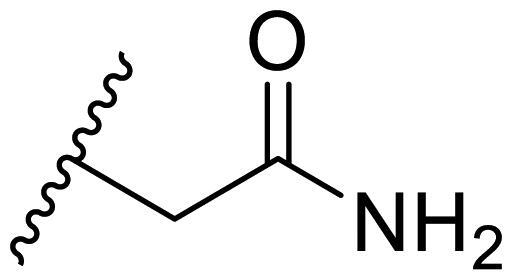	19.46 ± 2.28	>50	>50
**20e**	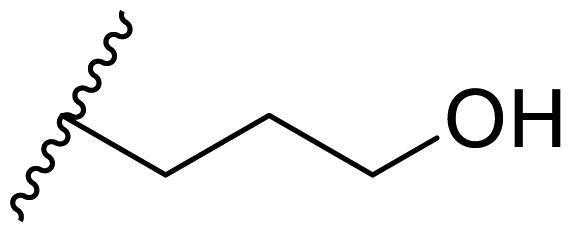	26.67 ± 3.39	39.13 ± 4.38	24.70 ± 3.63
**17-AAG**	--	10.60 ± 0.65	39.07 ± 1.30	0.45 ± 0.09

**Table 3.** Antiproliferative activity of 2,4-bis(benzyloxy)-5-arylpyrimidines **22**. 
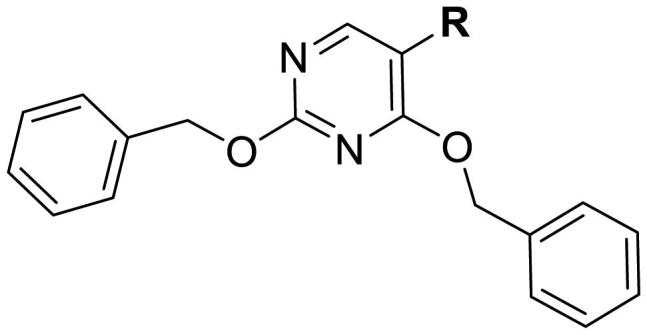

Cmpd.	R	IC_50_ (μM)		
		MCF-7	MDA-MB-231	4T1
**22a**	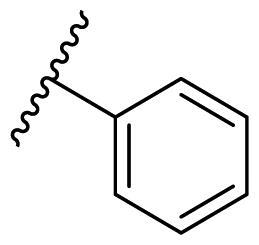	>50	>50	>50
**22b**	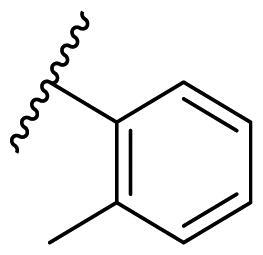	>50	>50	>50
**22c**	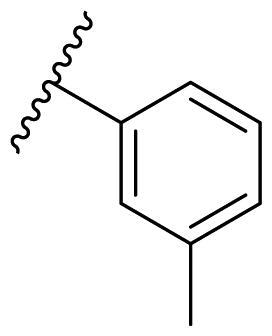	>50	>50	>50
**22d**	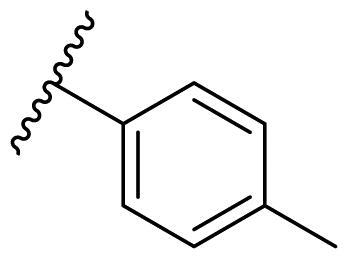	>50	>50	>50
**22e**	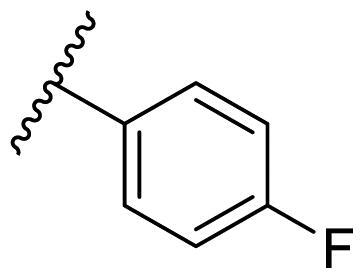	>50	>50	>50
**22f**	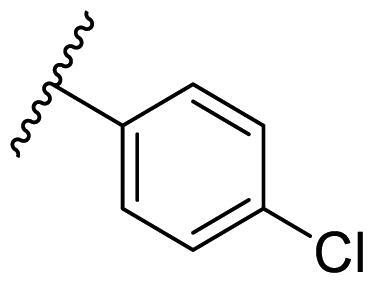	>50	>50	>50
**22g**	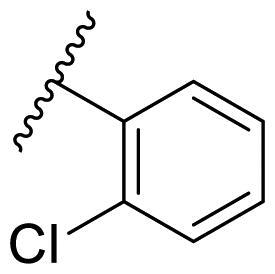	>50	>50	>50
**22h**	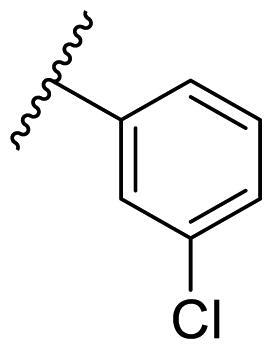	>50	>50	>50
**22i**	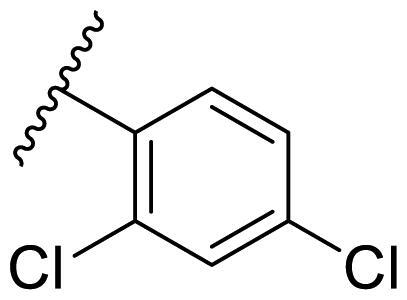	>50	>50	>50
**22j**	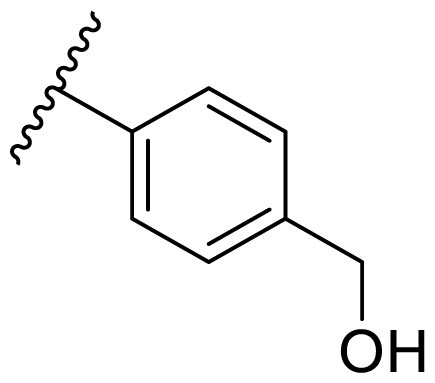	>50	>50	>50
**22k**	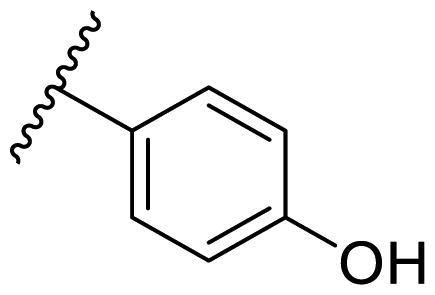	7.72 ± 0.86	7.89 ± 0.21	7.86 ± 0.76
**22l**	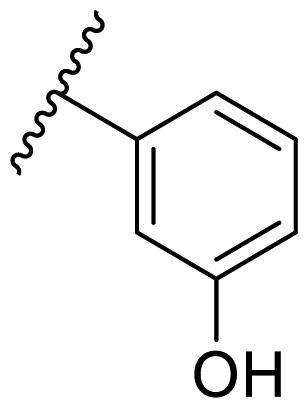	12.34 ± 0.50	11.17 ± 0.70	8.53 ± 0.50
**22m**	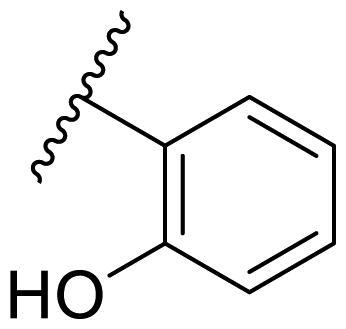	13.77 ± 0.28	12.66 ± 0.03	8.25 ± 0.68
**22n**	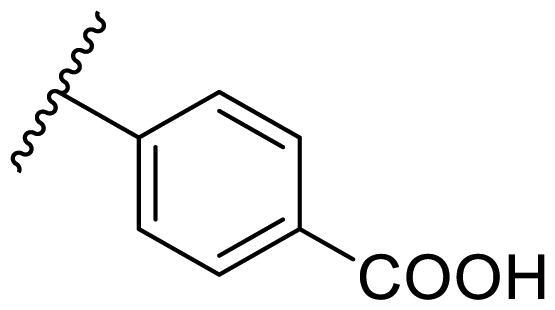	28.78 ± 2.09	>50	14.96 ± 2.03
**22o**	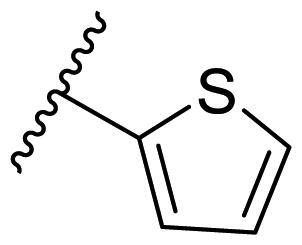	>50	>50	>50
**22p**	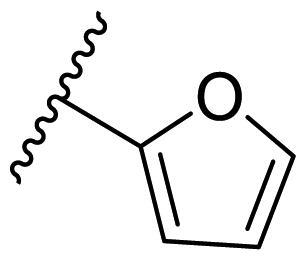	>50	>50	>50
**17-AAG**		10.60 ± 0.65	39.07 ± 1.30	0.45 ± 0.09

In the case of 2,4-bis(benzyloxy)-5-arylpyrimidines **22**, most compounds in this series were ineffective in inhibiting the proliferation of cancer cell lines (Table 3). However, the introduction of the hydroxyl group dramatically increased the activity. Compound **22k,** bearing a 4-hydroxyphenyl group at the C5 position of the pyrimidine ring, exhibited antiproliferative activity with IC_50_ values of 7.72 ± 0.86, 7.89 ± 0.21, and 7.86 ± 0.76 μM against three breast cancer cell lines. This result suggested that the hydroxyl group may play a crucial role in binding to the target.

#### Western blotting assay

**Figure 3.**
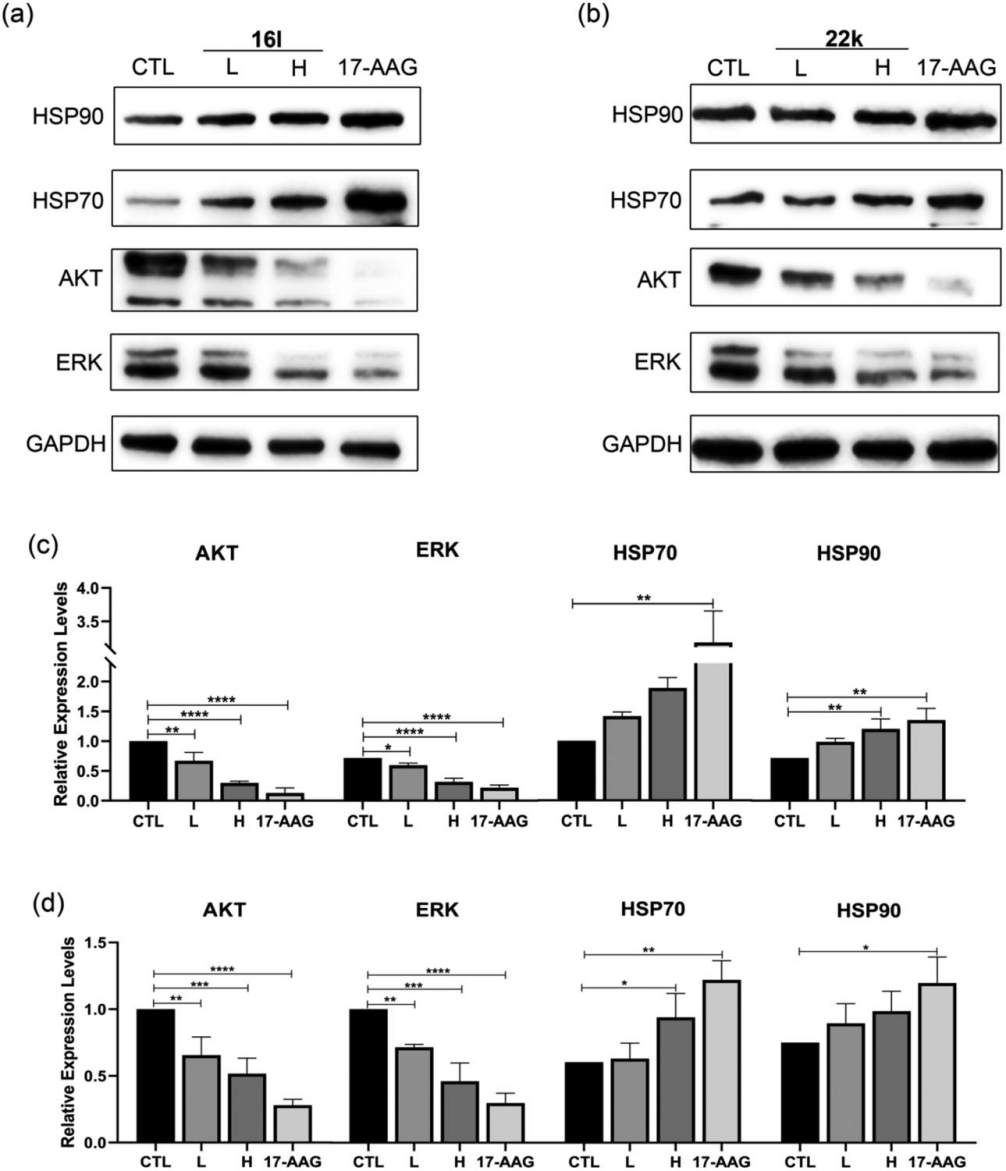
Western blotting assay. (a) (b) Effects of **16l** (L: 6 μM; H: 18 μM) and **22k** (L: 8 μM; H: 24 μM) on the expression of AKT, ERK, HSP70, and HSP90 in MCF-7 cells, with GAPDH as an internal reference. 17-AAG (15 μM) was used as a positive control. (c) (d) Statistical analysis of western blotting assays of **16l** and **22k**, respectively. Data are presented as the means ± SDs. *p < 0.05, **p < 0.01, ***p < 0.001 versus control.

Treating cells with HSP90 N-terminal inhibitors causes the degradation of HSP90 client proteins and the compensatory expression of heat shock proteins. Protein kinase B (also known as AKT) is a serine/threonine kinase and plays a key role in the PI3K signalling pathway^40^. An extracellular regulated kinase (ERK) is a member of the mitogen-activated protein kinase (MAPK) signalling pathway^41^. Both AKT and ERK are client proteins of HSP90 and are essential for cancer progression. The effect of **16l** and **22k** on the expression of the HSP90 client proteins AKT and ERK and the heat shock proteins HSP90 and HSP70 was evaluated by western blotting. As shown in Figure 3, both **16l** and **22k** significantly decreased the expression levels of AKT and ERK in MCF-7 cells, which was consistent with the features of the classical HSP90 N-terminal inhibitor 17-AAG. It is interesting that, even at a concentration 3 times higher than the IC_50_ value, the expression level of HSP70 was not significantly increased in the **16l**-treated MCF-7 cells, as was the expression level of HSP90 in the **22k** treated cells. This result suggested that **16l** and **22k** inhibited cancer cell proliferation with a lower level of heat shock response in comparison with 17-AAG.

#### HSP90 binding affinity evaluation using FP assay

**Figure 4.**
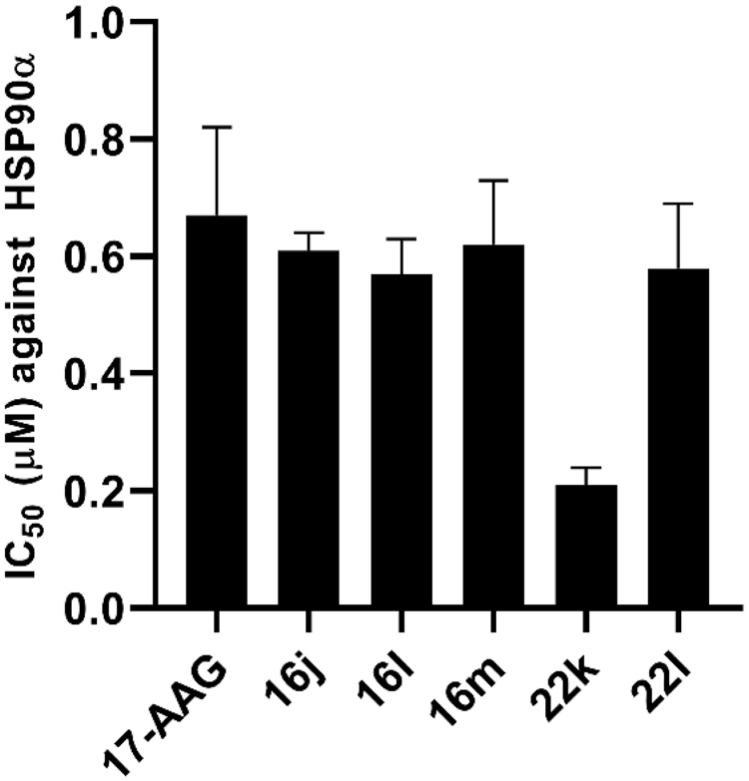
Binding affinity evaluation of representative compounds to HSP90 N-terminus.

To further verify the binding of the synthesised compounds to the HSP90 N-terminus, the binding affinity of five typical compounds (**16j**, **16l**, **16m**, **22k**, and **22l**) was evaluated using an FP assay. The addition of compounds with HSP90 inhibitory capacity into the assay would compete with the fluorescence probe for binding into HSP90, resulting in a decrease in FP in comparison with the probe alone^42^. As shown in Figure 4, all five compounds exhibited similar binding affinities to HP90 in comparison with 17-AAG. Among them, **22k** had the strongest affinity for the HSP90 N-terminus, with an IC_50_ value of 0.21 ± 0.03 μM.

### Molecular docking study

**Figure 5.**
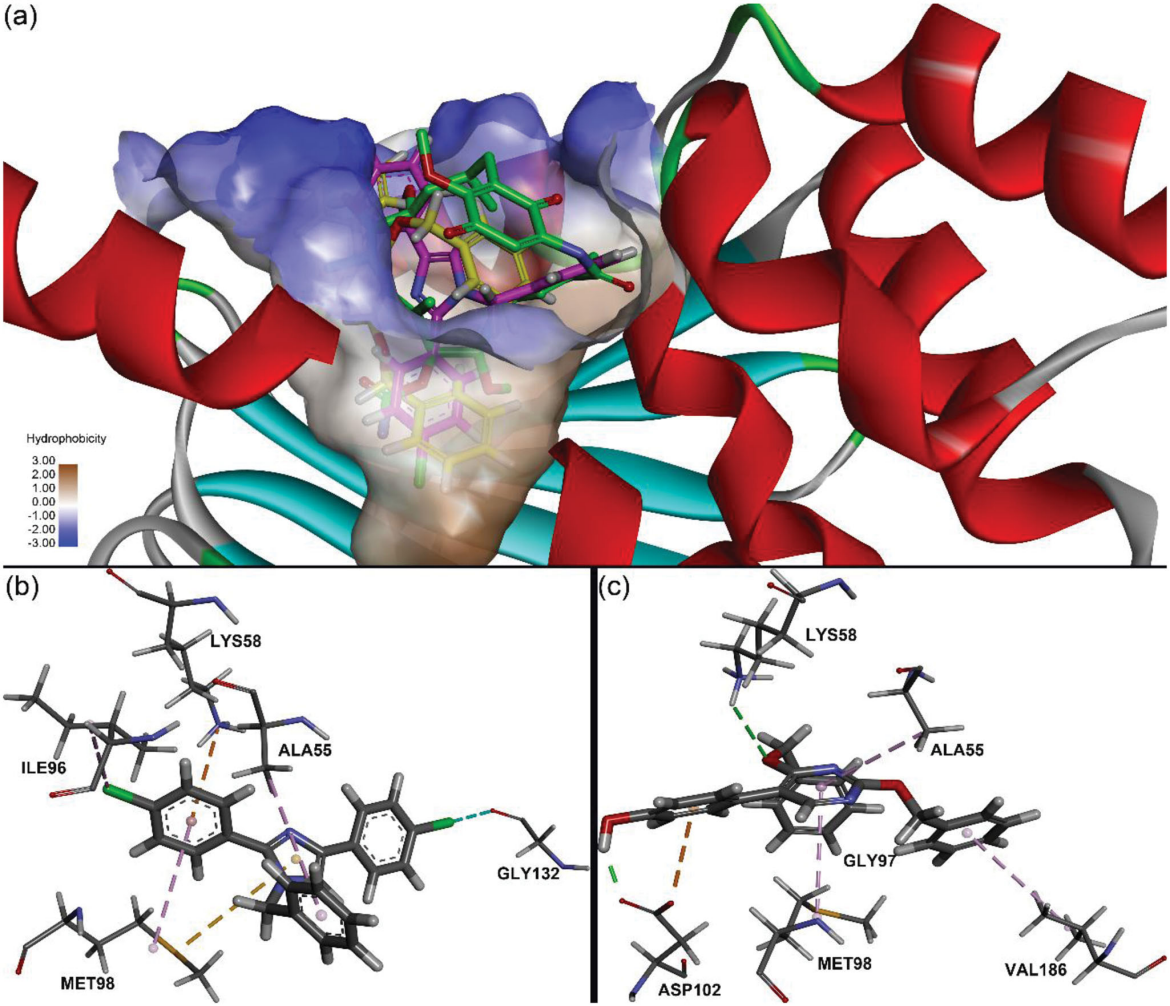
Predicted binding modes of **16l** and **22k** to the N-terminus of HSP90α (PDB code: 1YET). (a) Overlap of **16l** (purple), **22k** (yellow) and geldanamycin (green) in the hydrophobic bond surface of the binding pocket; (b) Interactions between **16l** and HSP90α residues; (c) Interactions between **22k** and HSP90α residues.

Molecular docking was performed to predict the binding modes of **16l** and **22k** with the HSP90α N-terminus. Because the FP assay revealed that **16l** and **22k** could competitively bind to the geldanamycin binding site at the HSP90α N-terminus, a crystal structure of the HSP90α N-terminus and geldanamycin (PDB code: 1YET) was used to generate the receptor structure, and the geldanamycin binding site was employed as the active site for docking. To test the feasibility of our docking method, we docked geldanamycin into the prepared HSP90α N-terminus. The resulting top 10 binding poses were very similar to the experimental pose, with all RMSD values less than 1 Å, which suggested that our docking procedure could afford good pose reproduction (Figure S115 in the supporting information). The top-scoring pose has a -CDOCKER_INTERACTION_ENERGY score of 65.32 with an RMSD value of 0.70. As shown in Figure 5(a), **16l** and **22k** were successfully docked into the binding pocket of geldanamycin at the N-terminus of HSP90α with -CDOCKER_INTERACTION_ENERGY scores of 36.36 and 55.08, respectively.

**16l** presented a “T-shaped lock” conformation and was possibly able to block the entrance of ATP into the binding pocket. The benzyloxy group at the N1 position of **16l** was directed towards the bottom of the binding pocket, while two 4-chlorophenyl groups were positioned at the mouth of the binding pocket. **16l** may form a halogen bond with GLY132, a Pi-cation interaction with LYS58, a Pi-sulphur interaction with MET98, and multiple hydrophobic interactions with residues ALA55, ILE96, and MET98 (Figure 5b). In the case of **22k**, the benzyloxy group at the C2 position of the pyrimidine ring was oriented towards the bottom of the binding pocket. It should be noted that **22k** may have formed hydrogen bonds with LYS58 and ASP102 (Figure 5c). The hydrogen bond interaction between the hydroxyl group of **22k** and ASP102 indicated that the hydroxyl group may be important for stabilising the protein-ligand complex. This result is consistent with the observations in the biological evaluation that the introduction of the hydroxyl group enhanced the antiproliferative activity. In addition, hydrophobic interactions with residues ALA55, MET98, and VAL186 and a Pi-Anion interaction with ASP102 were observed in the predicted binding mode of **22k** and the HSP90 N-terminus.

## Conclusion

In summary, our work discovered two kinds of novel HSP90 N-terminal inhibitors bearing 2,4-diarylimidazole and 2,4-bis(benzyloxy)-5-arylpyrimidine as their scaffolds. **16l** and **22k** exhibited strong antiproliferative activities against three breast cancer cell lines, MCF-7, MDA-MB-231, and 4T1. Their inhibitory activity towards the HSP90 N-terminus was validated by western blotting and FP assays, and possible interaction modes were predicted by molecular docking. **16l** and **22k** can serve as a starting point for more in-depth research on anticancer drugs targeting the HSP90 N-terminus. Our future efforts will focus on evaluating the anticancer activity *in vivo* and investigating the pharmacokinetic behaviours of **16l** and **22k**.

## Supplementary Material

Supplemental Material
