# Supplementary material for: Design, synthesis, biological evaluation and molecular docking study of 2,4-diarylimidazoles and 2,4-bis(benzyloxy)-5-arylpyrimidines as novel HSP90 N-terminal inhibitors
Source: J Enzyme Inhib Med Chem. 2022 Sep 19;37(1):2551–65. doi: 10.1080/14756366.2022.2124407 (PMC9518286; doi:10.1080/14756366.2022.2124407)
Supplement: Supplemental Material [file IENZ_A_2124407_SM9082.pdf]

Supporting Information

Design, synthesis, biological evaluation and molecular docking study of 2, 4-diarylimidazoles and 2, 4-bis(benzyloxy)-5-arylpyrimidines as novel HSP90 N-terminal inhibitors

Man Yang, Chenyao Li, Yajing Li, Chen Cheng, Meiyun Shi, Lei Yin, Hongyu Xue, Yajun Liu*

School of Life and Pharmaceutical Sciences, Dalian University of Technology, Dagong Road 2, Liaodongwan district, Panjin 124221, China.

Corresponding author: Yajun Liu, E-mail: yjliu85@dlut.edu.cn, Fax: +86-427-263-1899.

1. Spectra for synthesized compounds

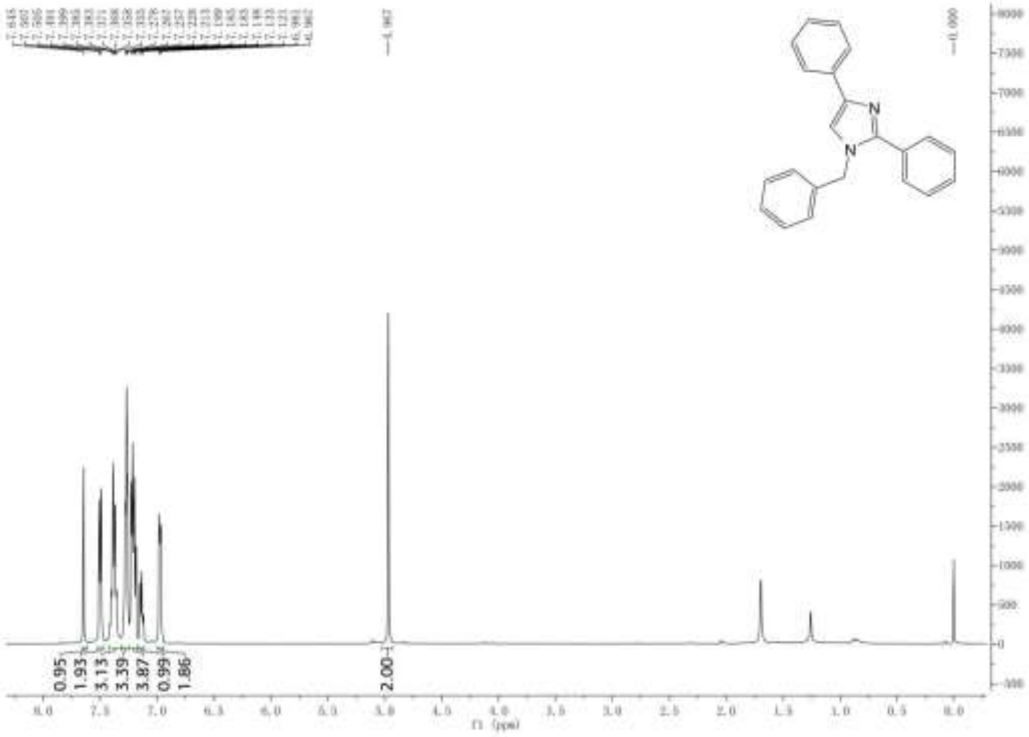

Figure S1. ^{1}H NMR spectrum of **16a**

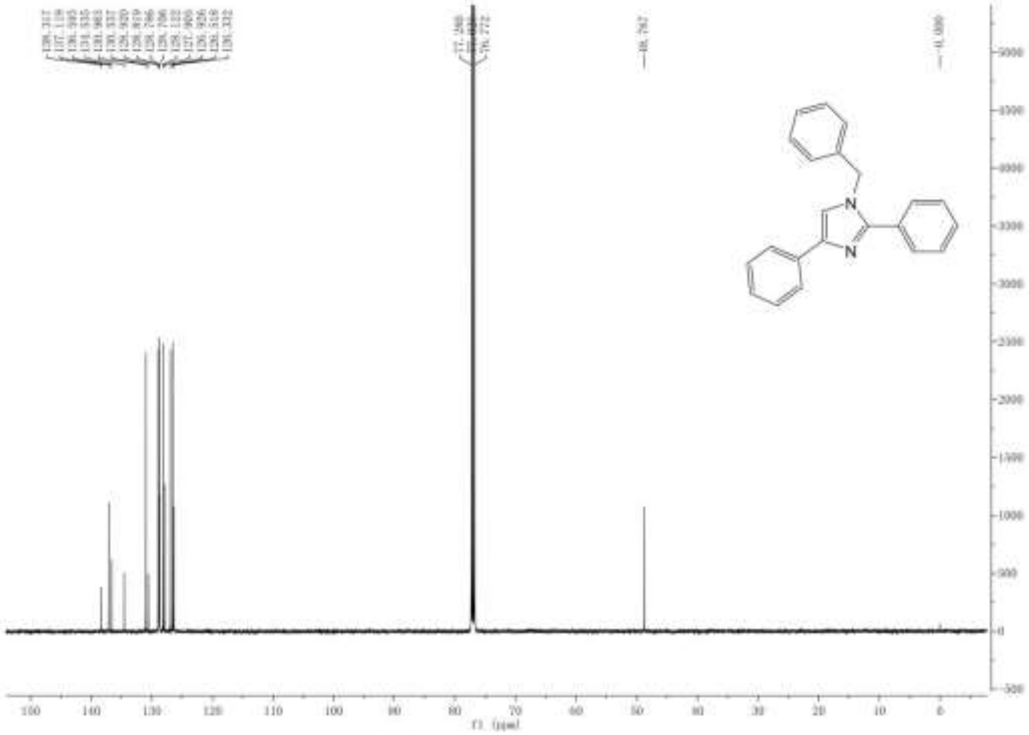

Figure S2. ^{13}C NMR spectrum of **16a**

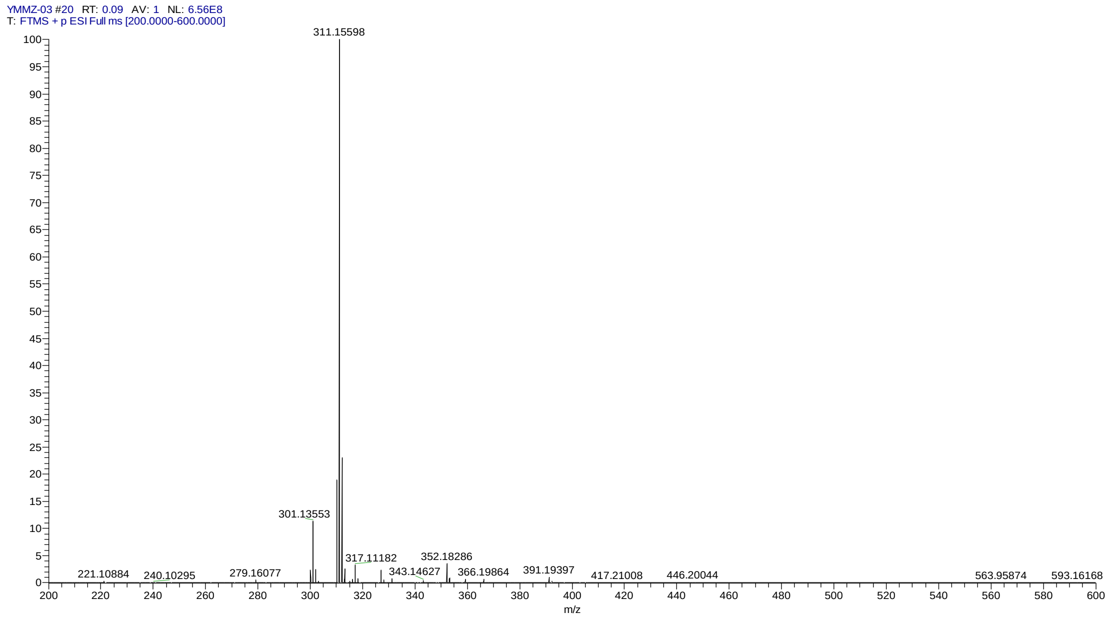

Figure S3. HRMS spectrum of **16a**

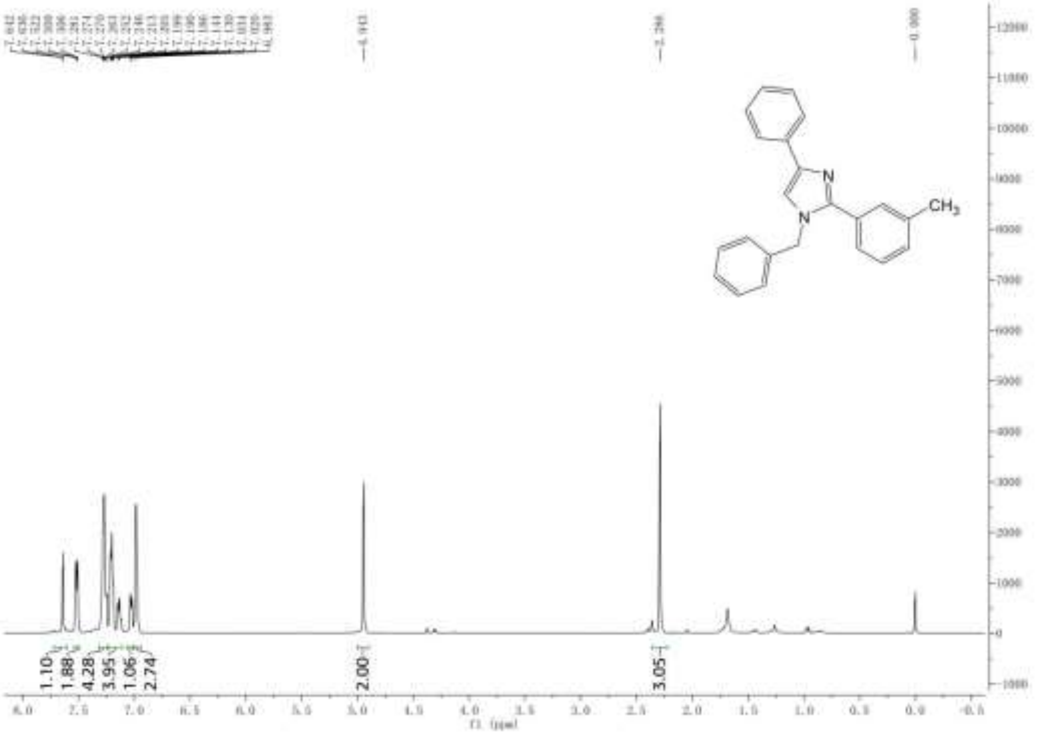

Figure S4. ^{1}H NMR spectrum of **16b**

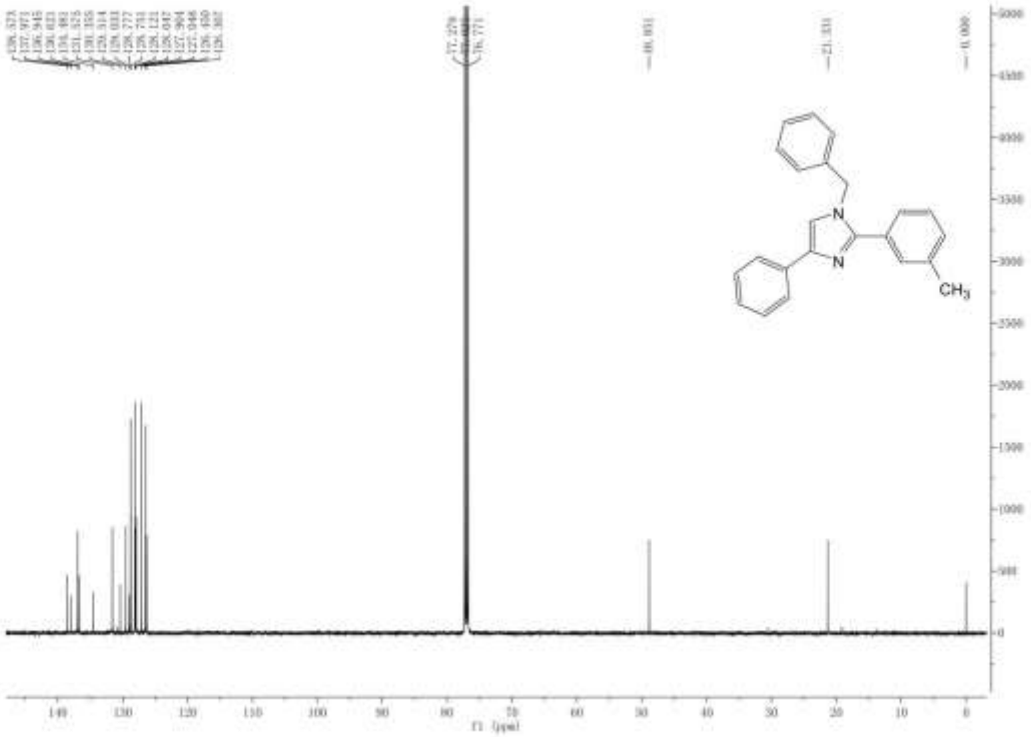

Figure S5. ^{13}C NMR spectrum of **16b**

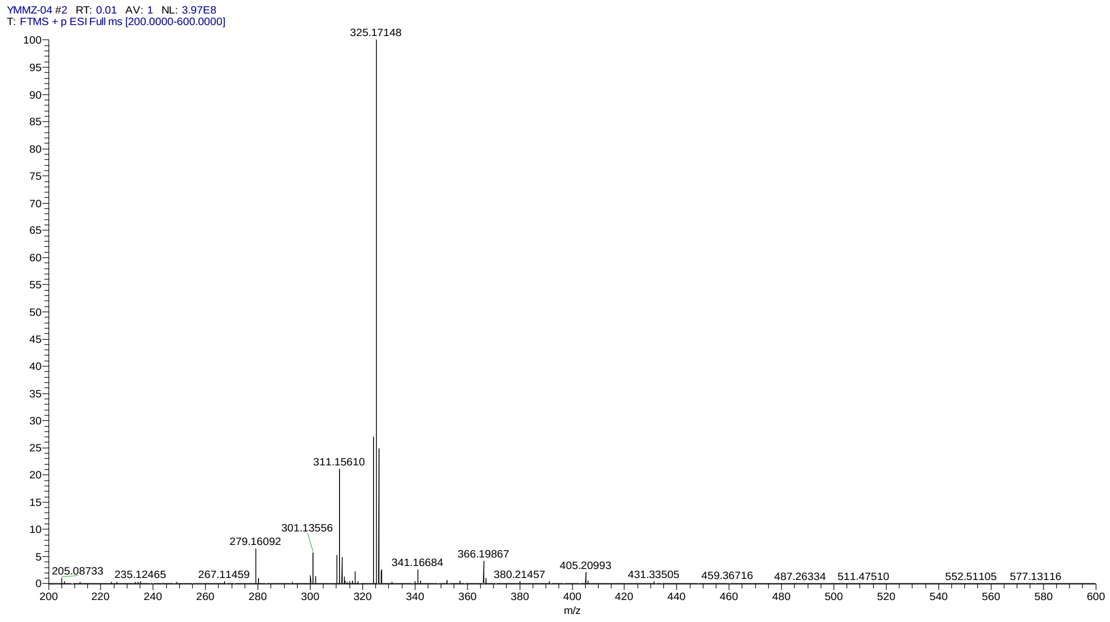

Figure S6. HRMS spectrum of **16b**

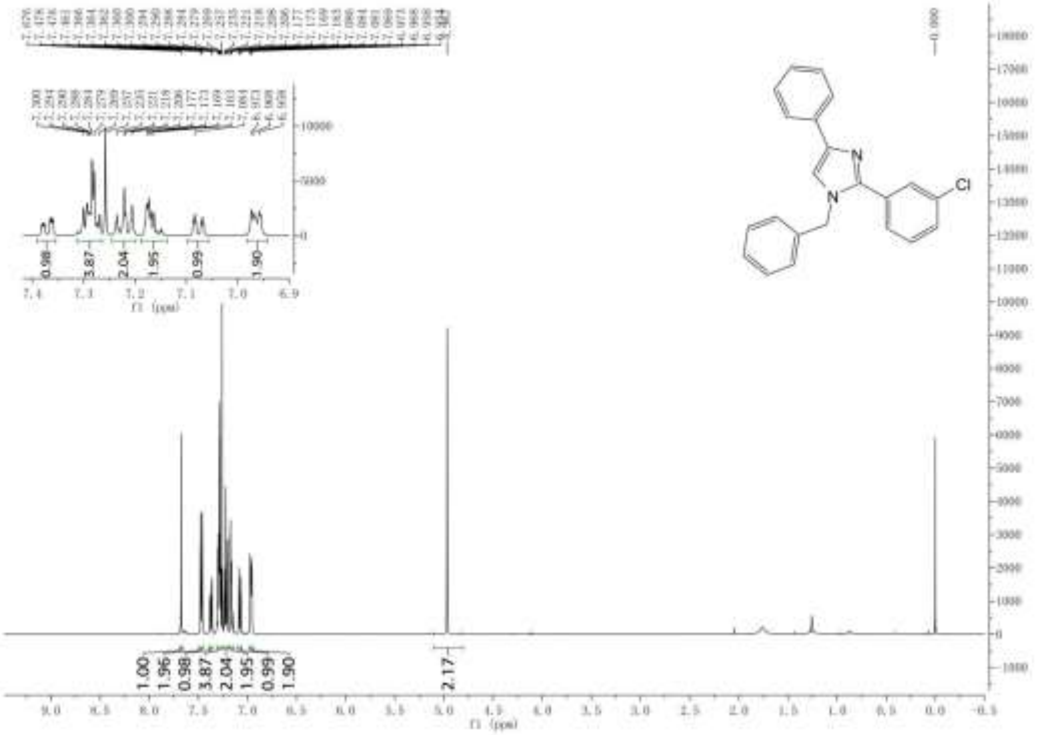

Figure S7. ^{1}H NMR spectrum of **16c**

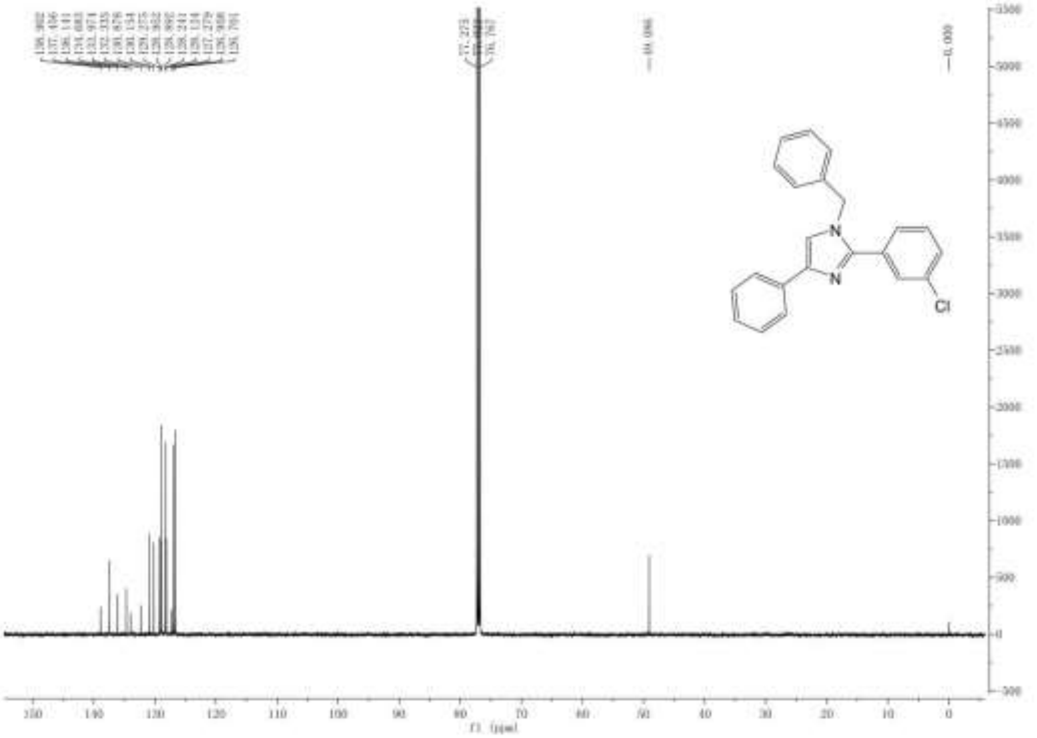

Figure S8. ^{13}C NMR spectrum of **16c**

Information Classification: General

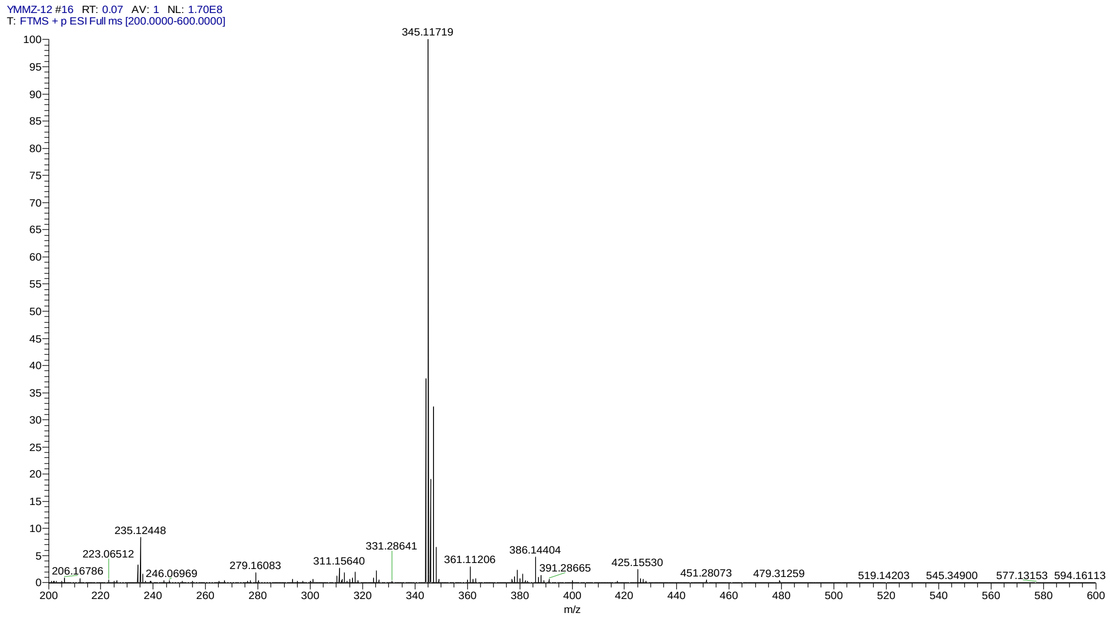

Figure S9. HRMS spectrum of **16c**

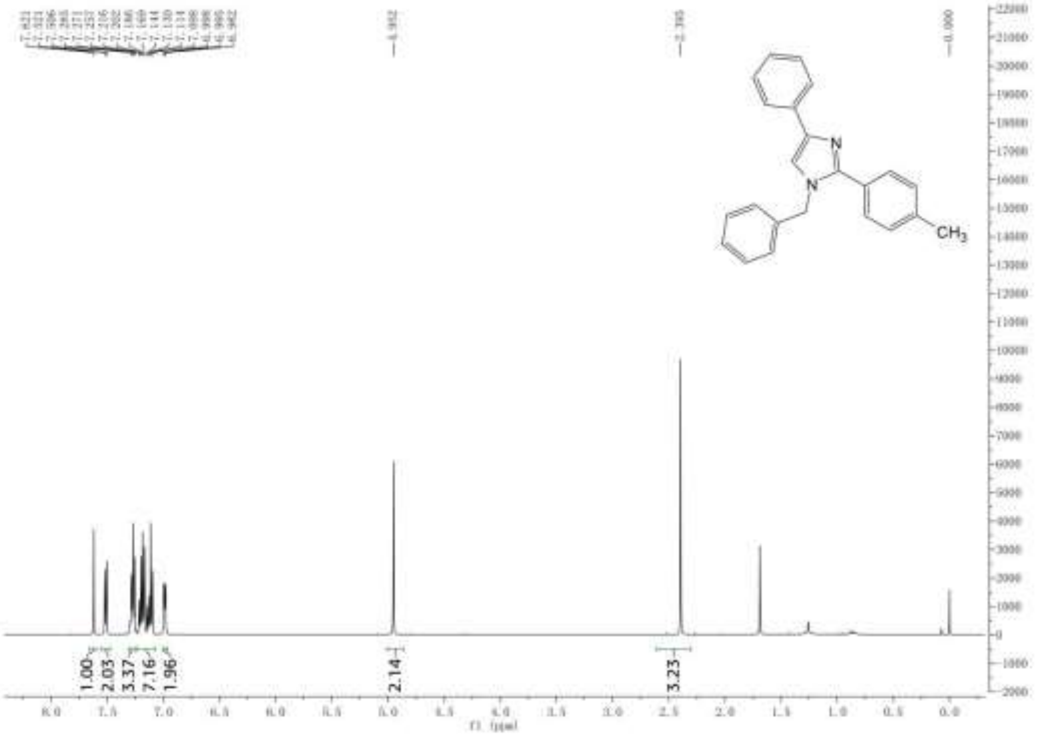

Figure S10. ^{1}H NMR spectrum of **16d**

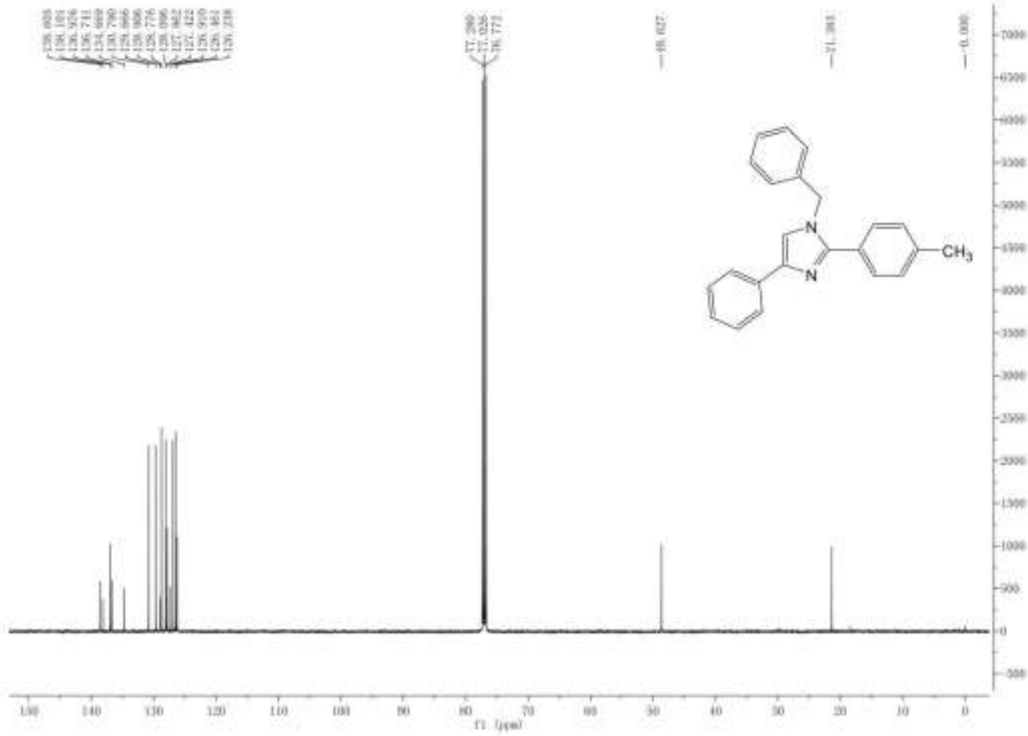

Figure S11. ^{13}C NMR spectrum of **16d**

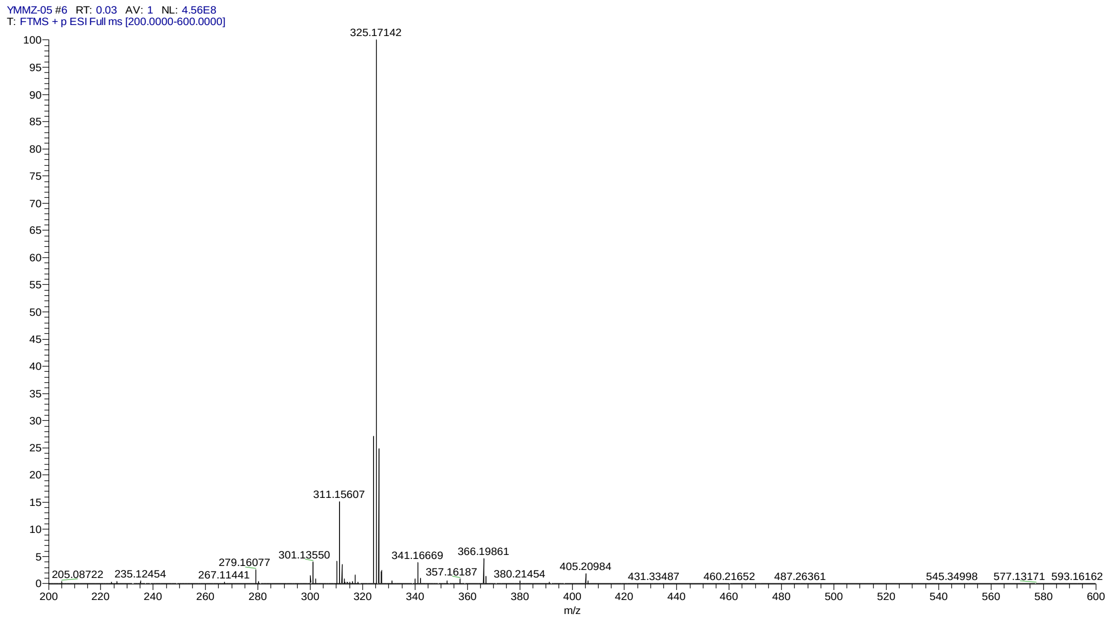

Figure S12. HRMS spectrum of **16d**

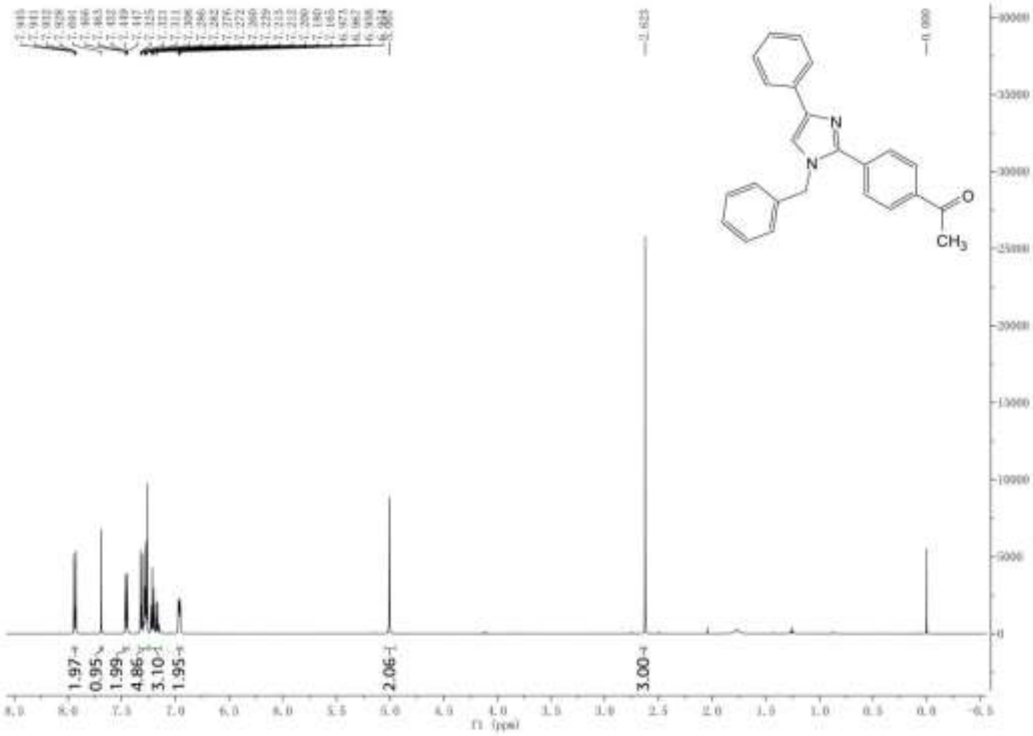

Figure S13. ^{1}H NMR spectrum of **16e**

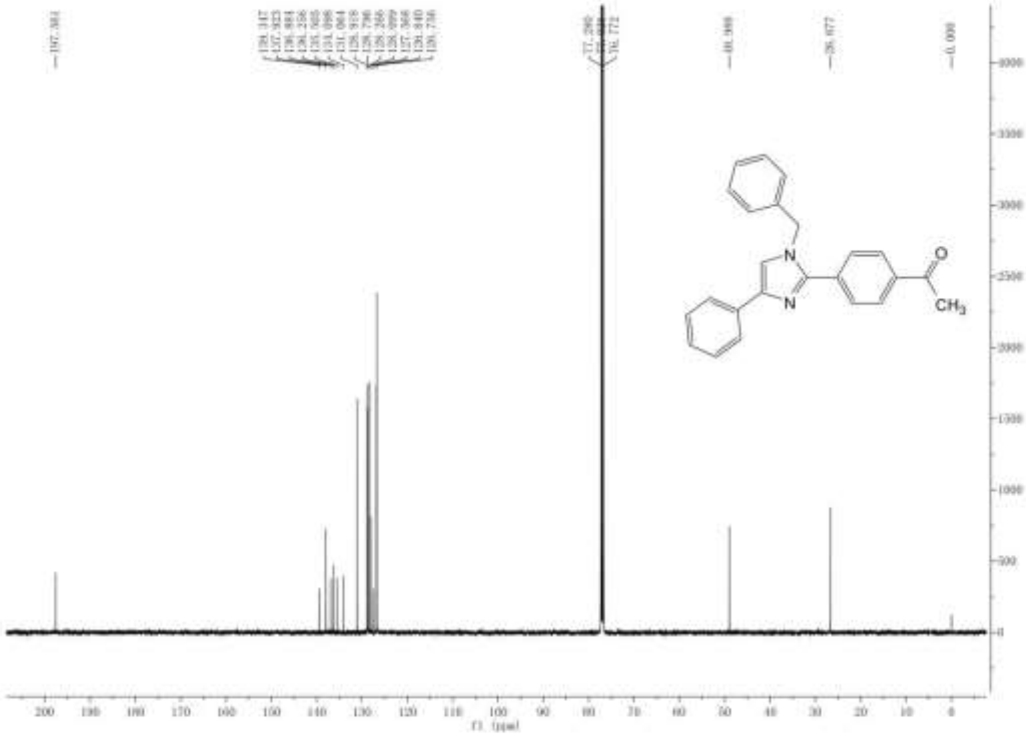

Figure S14. ^{13}C NMR spectrum of **16e**

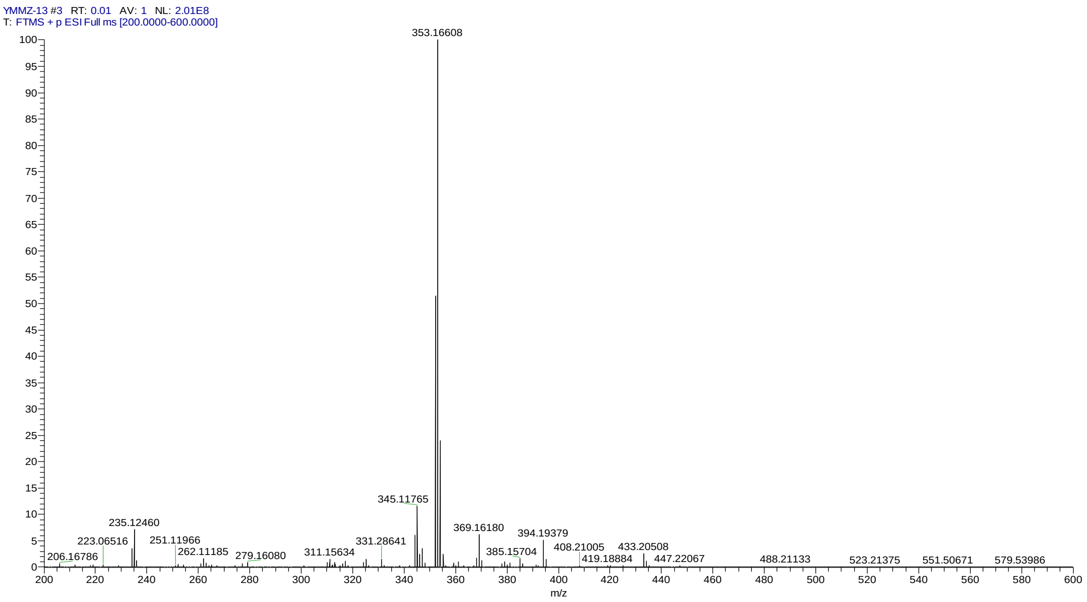

Figure S15. HRMS spectrum of **16e**

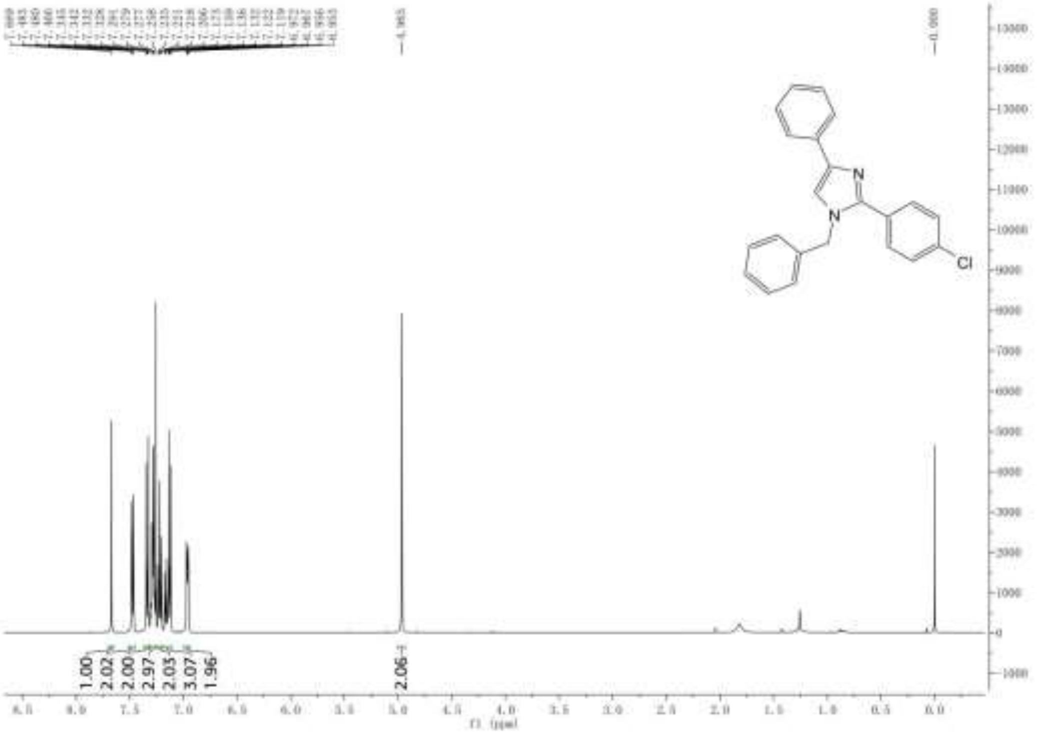

Figure S16. ^{1}H NMR spectrum of **16f**

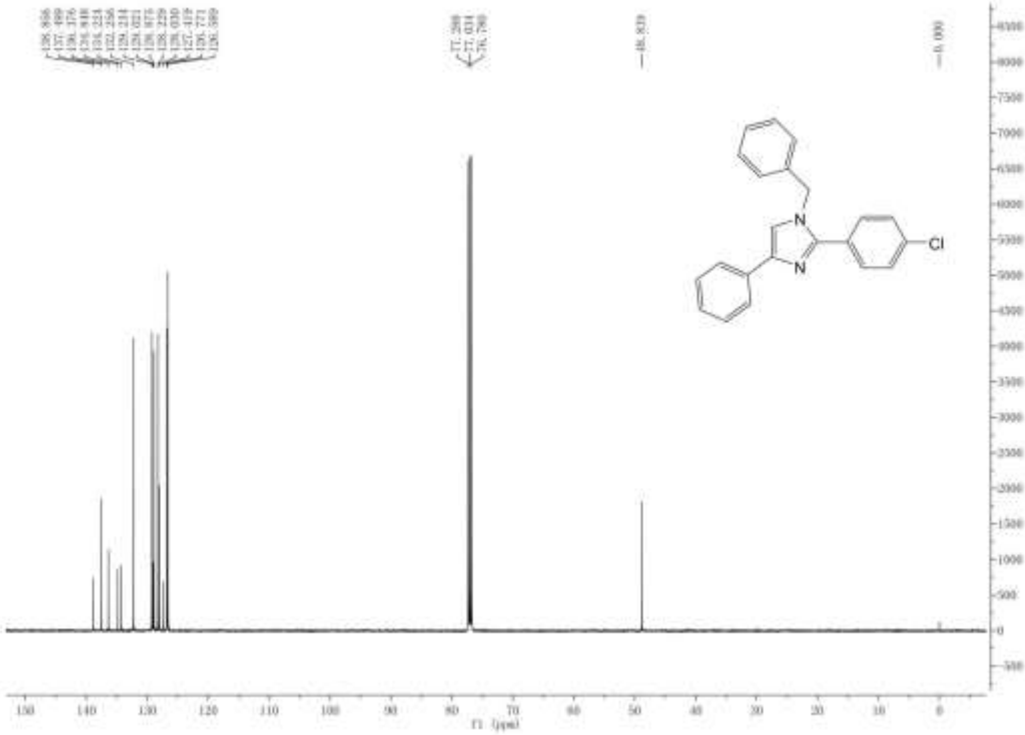

Figure S17. ^{13}C NMR spectrum of **16f**

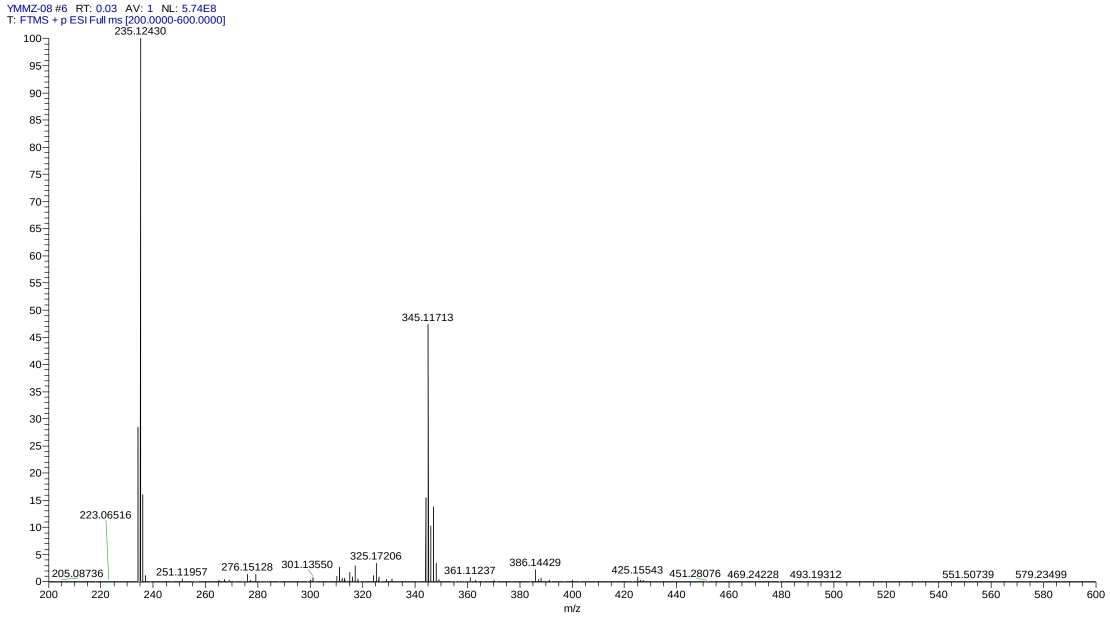

Figure S18. HRMS spectrum of **16f**

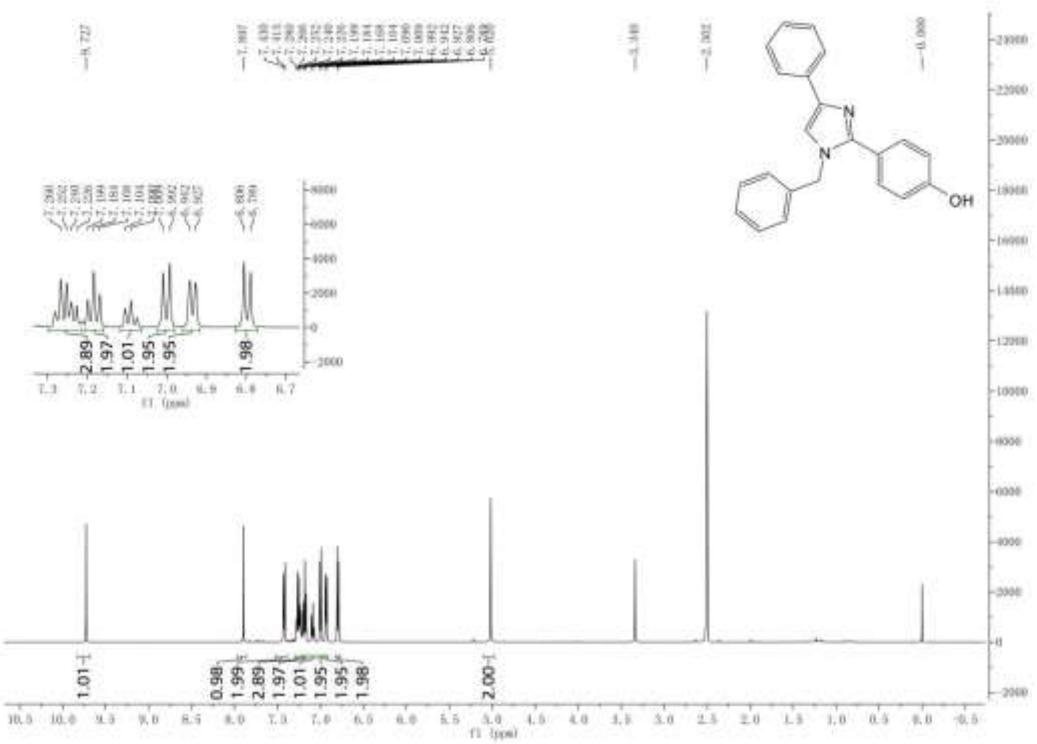

Figure S19. ^{1}H NMR spectrum of **16g**

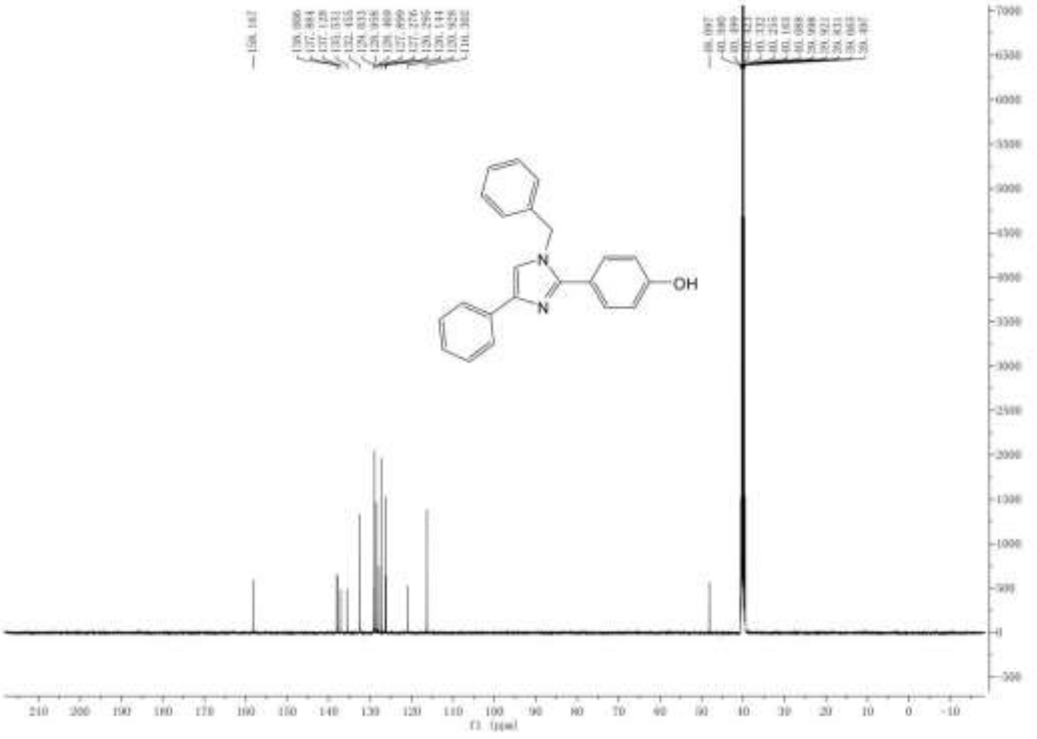

Figure S20. ^{13}C NMR spectrum of **16g**

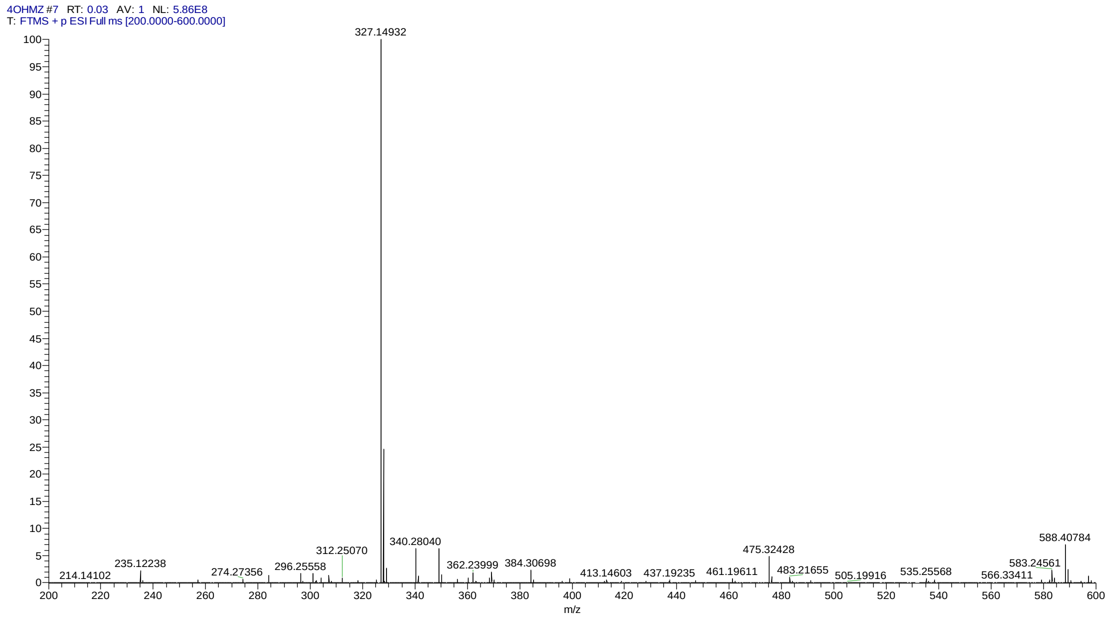

Figure S21. HRMS spectrum of **16g**

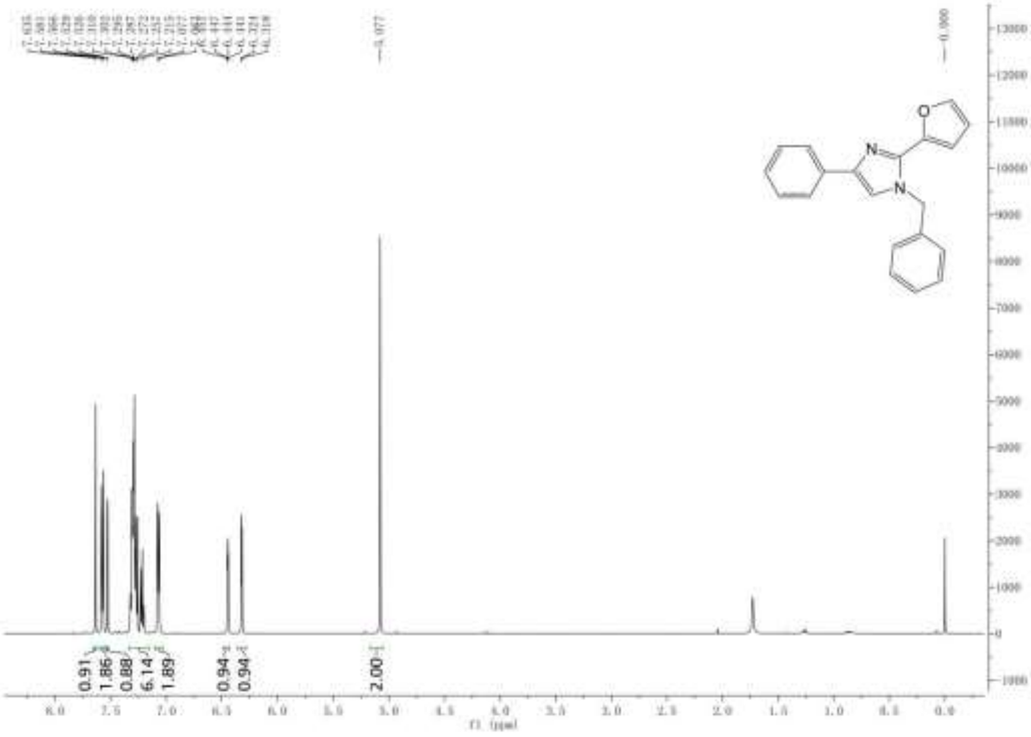

Figure S22. ^{1}H NMR spectrum of **16h**

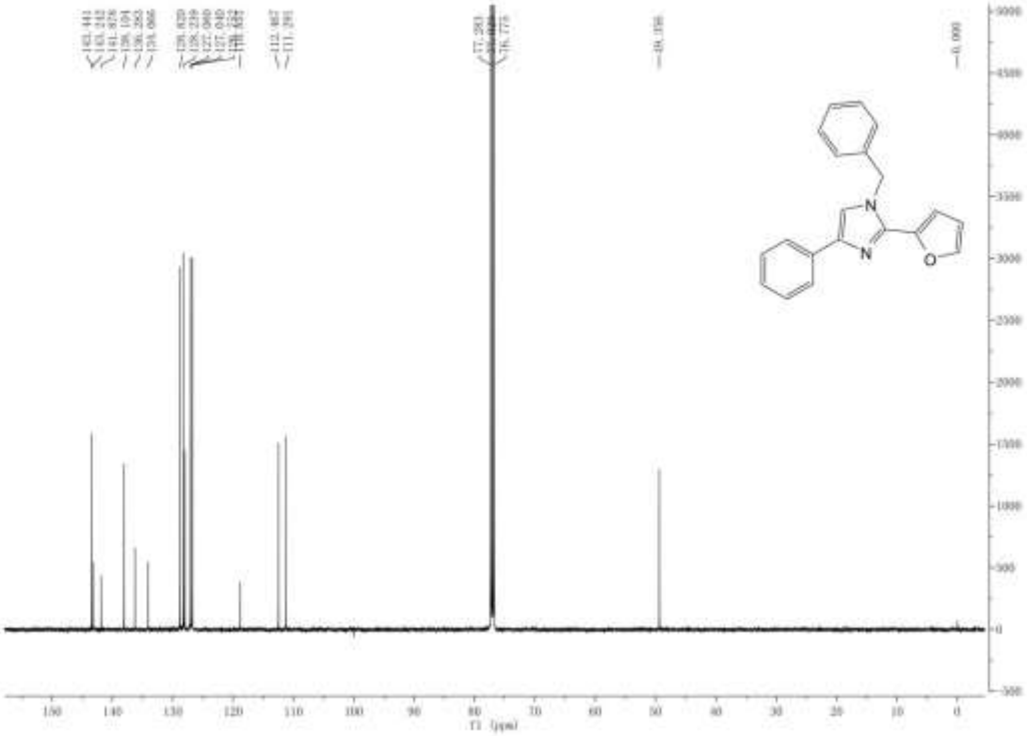

Figure S23. ^{13}C NMR spectrum of **16h**

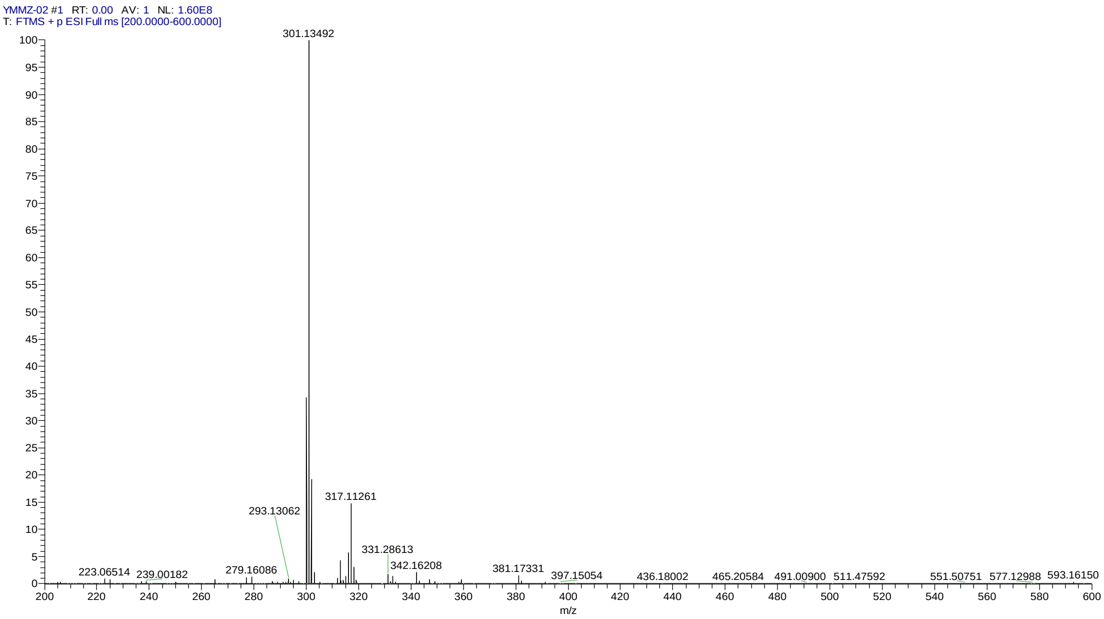

Figure S24. HRMS spectrum of **16h**

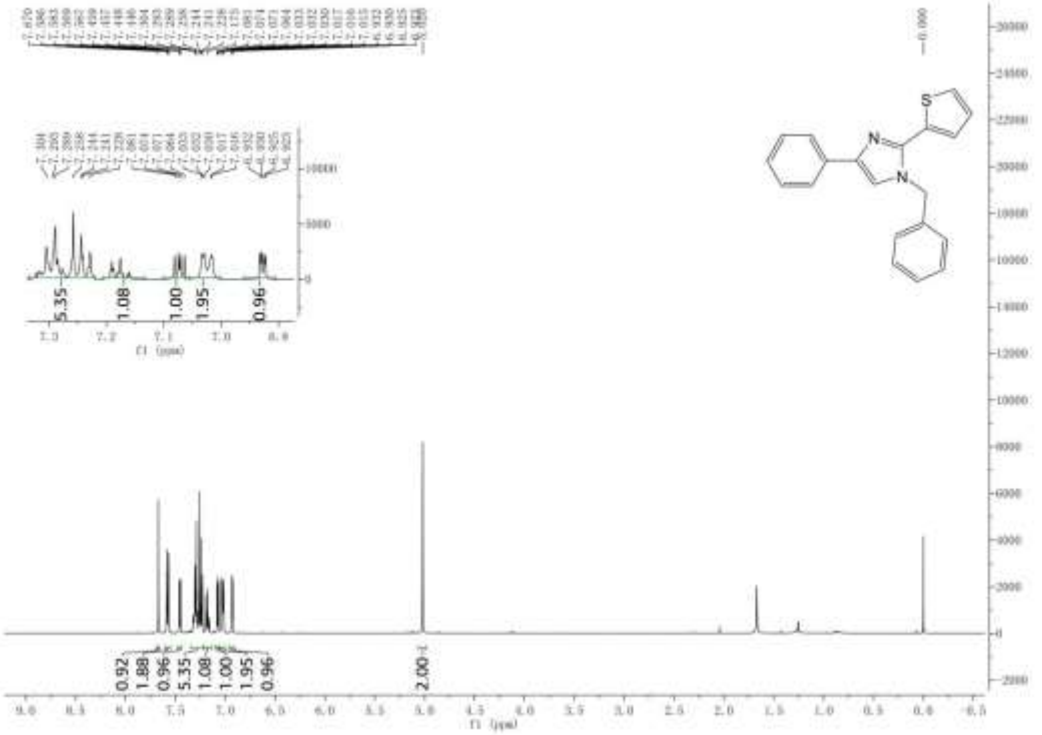

Figure S25. ^{1}H NMR spectrum of **16i**

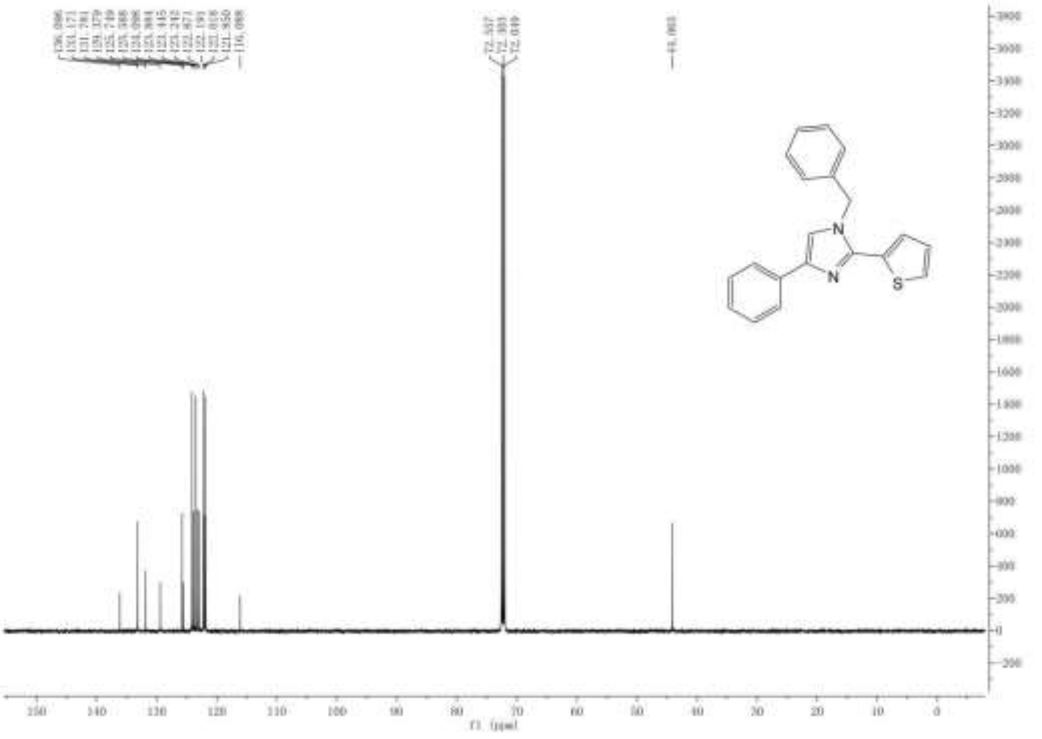

Figure S26. ^{13}C NMR spectrum of **16i**

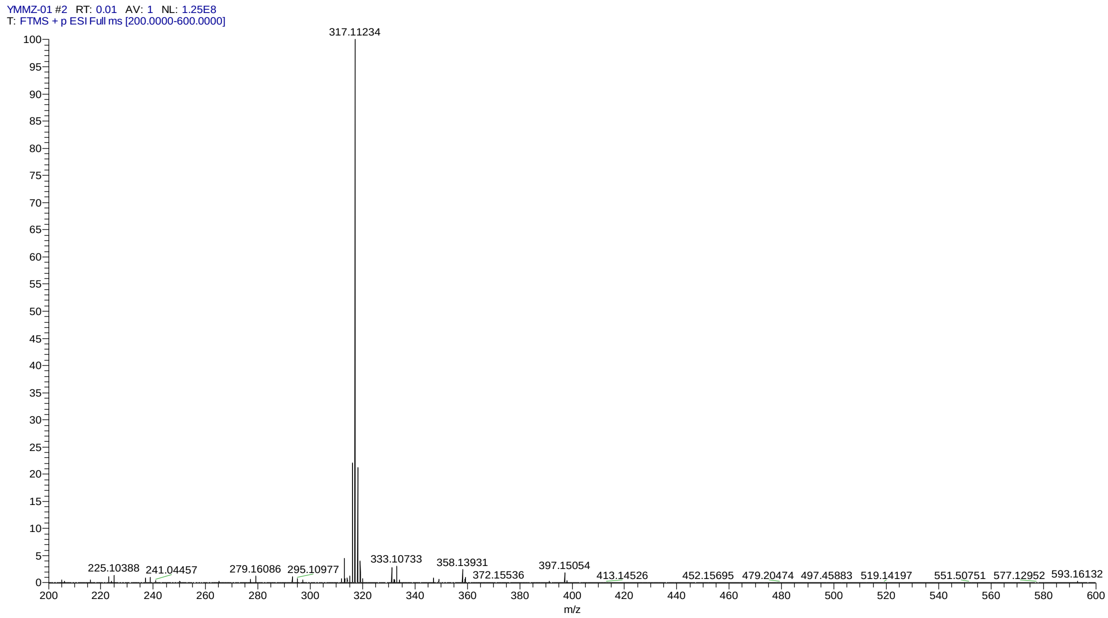

Figure S27. HRMS spectrum of **16i**

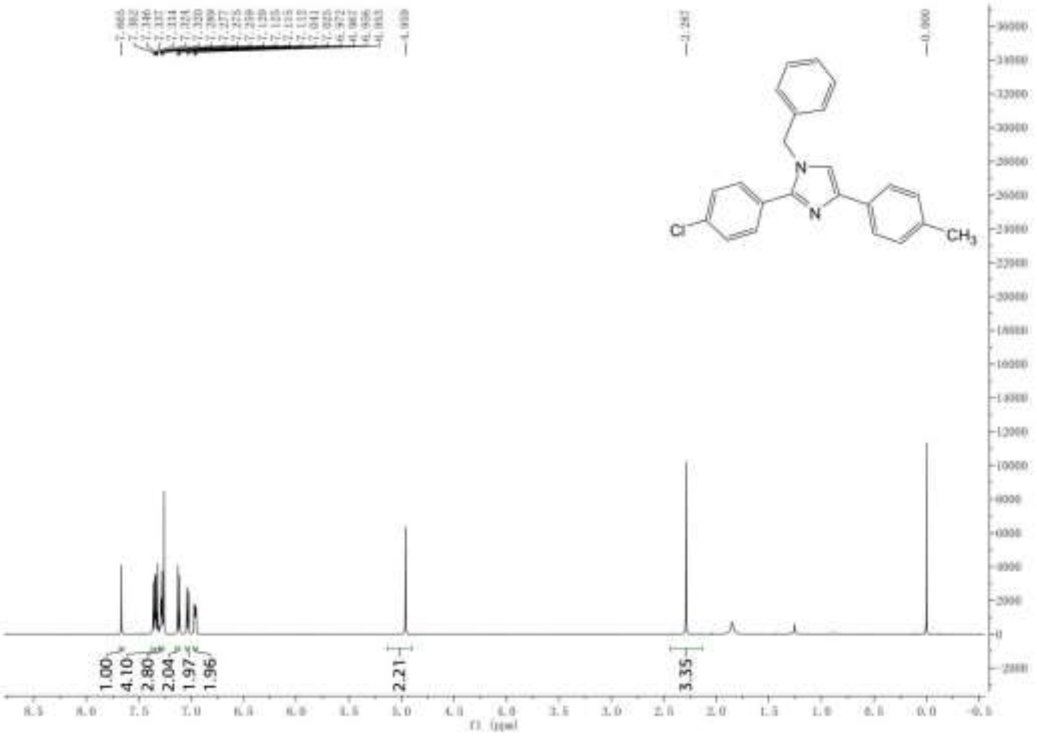

Figure S28. ^{1}H NMR spectrum of **16j**

Information Classification: General

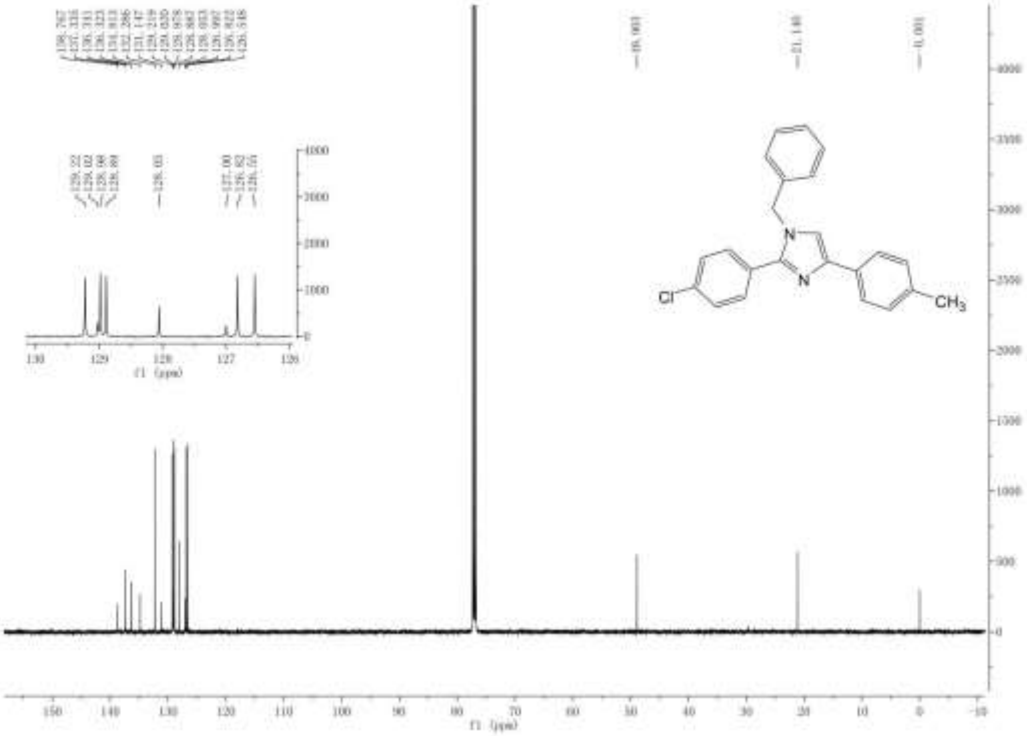

Figure S29. ^{13}C NMR spectrum of **16j**

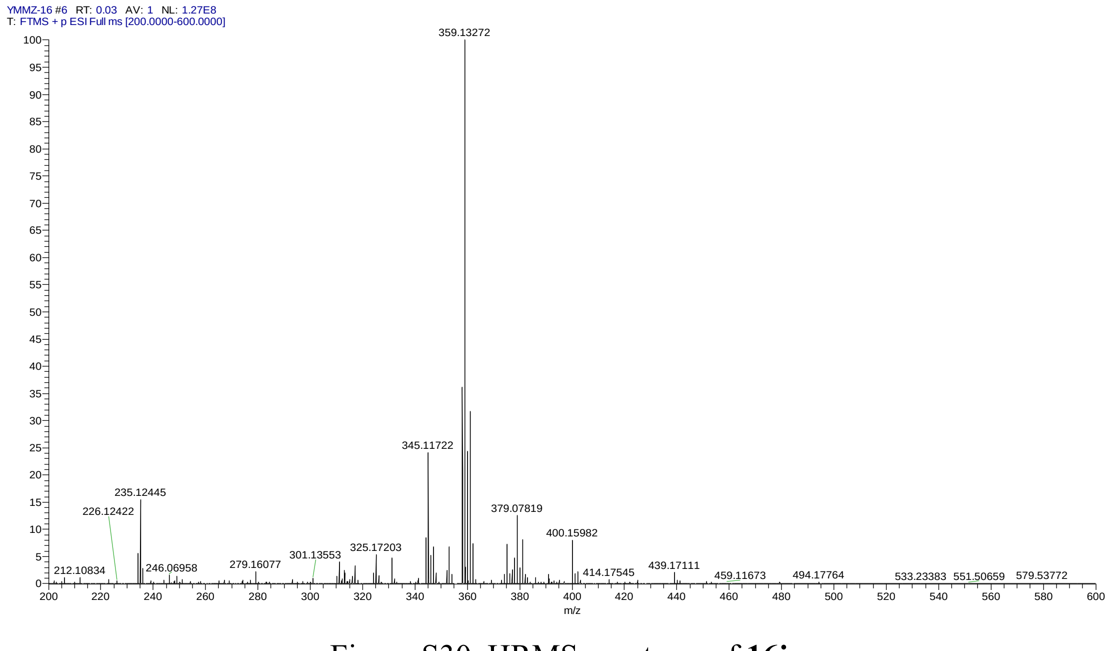

Figure S30. HRMS spectrum of **16j**

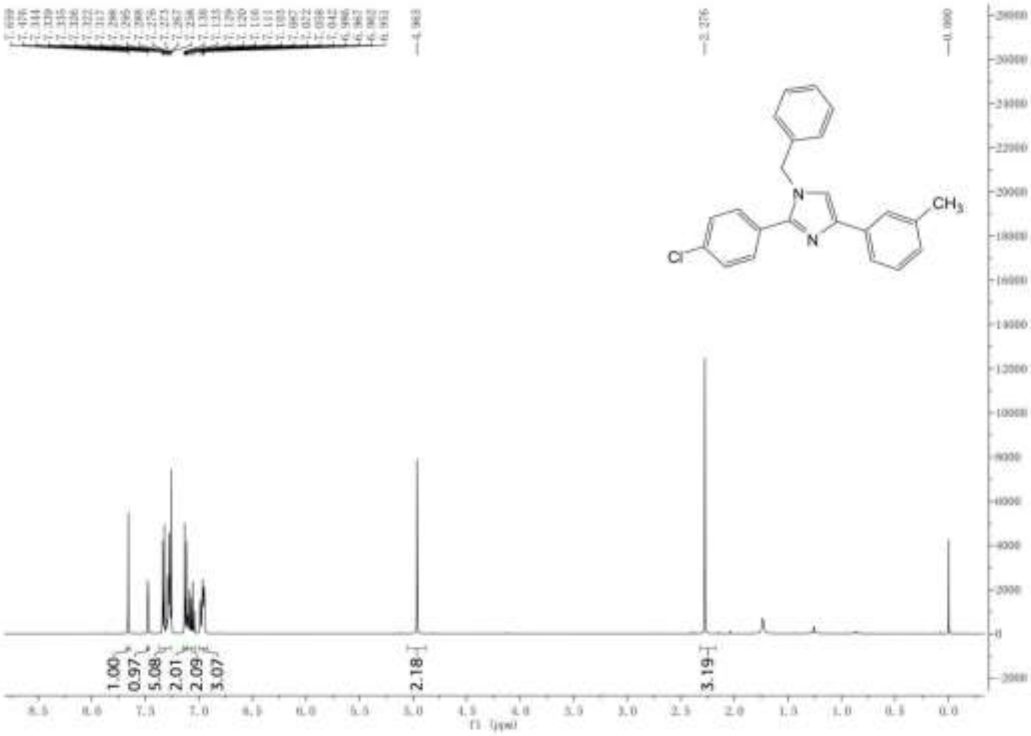

Figure S31. ^{1}H NMR spectrum of **16k**

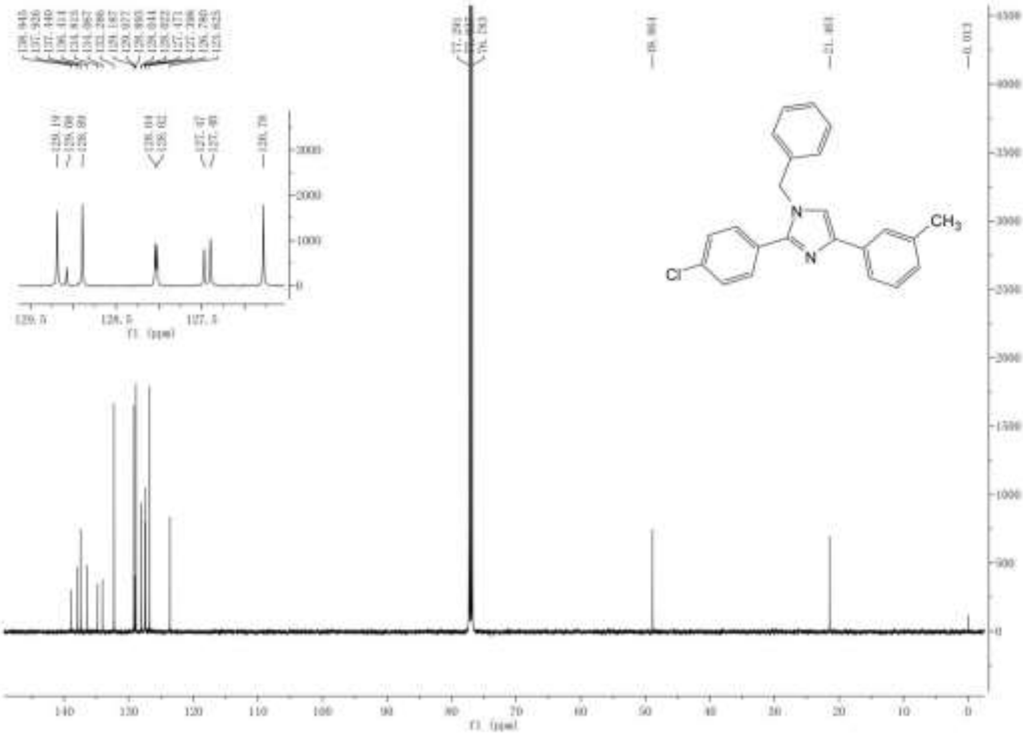

Figure S32. ^{13}C NMR spectrum of **16k**

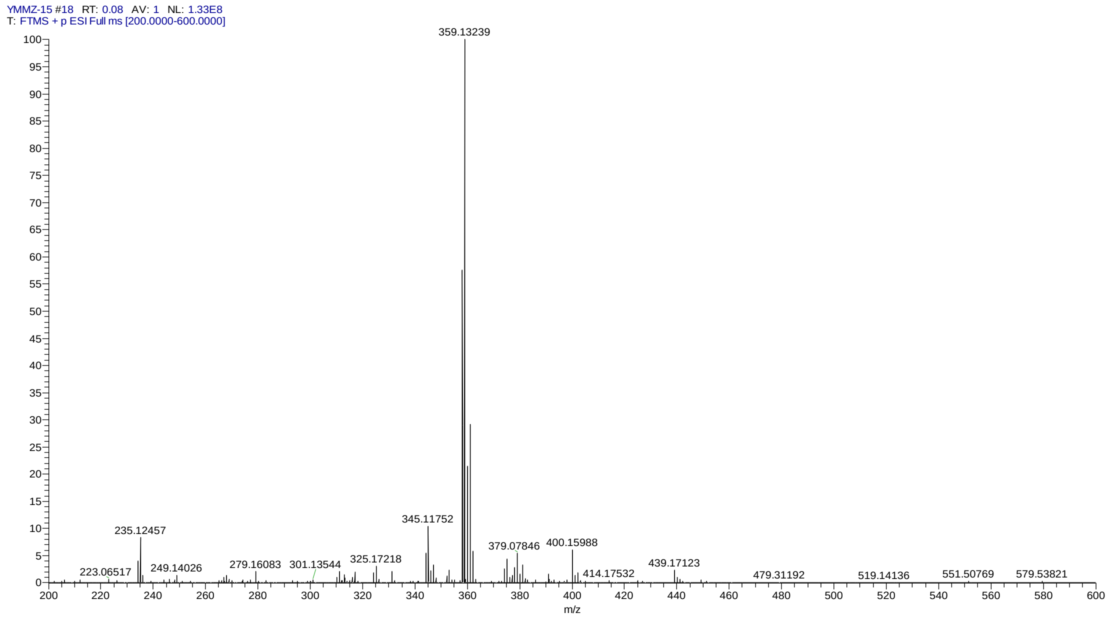

Figure S33. HRMS spectrum of **16k**

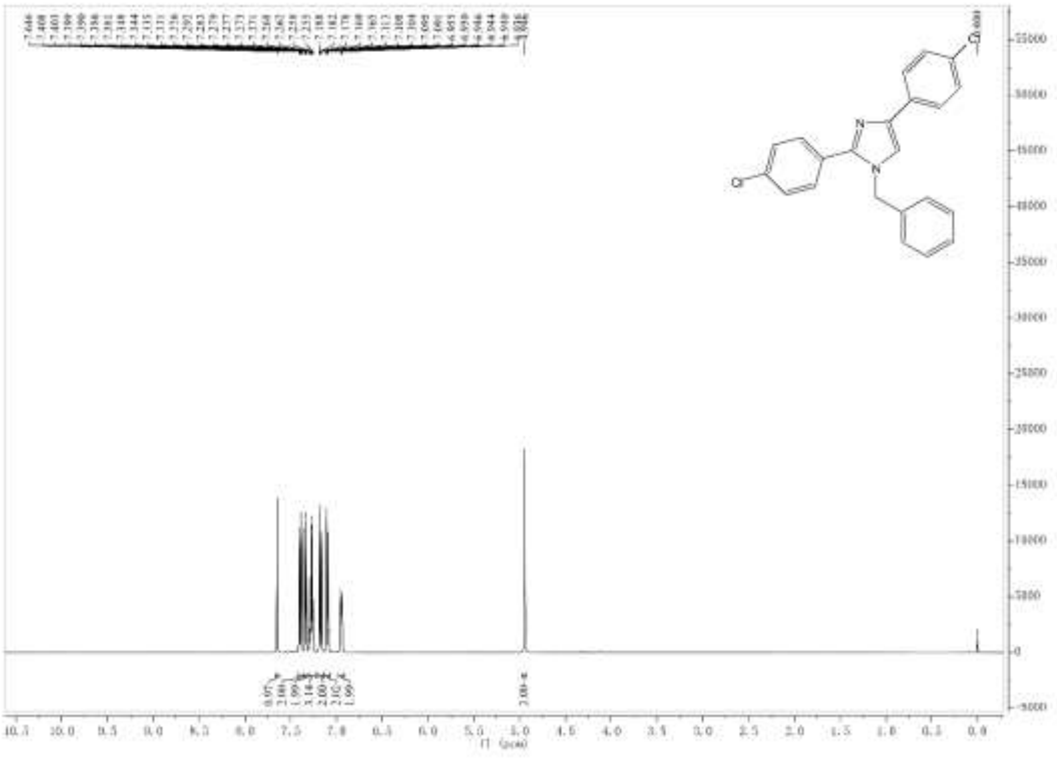

Figure S34. ^{1}H NMR spectrum of **16l**

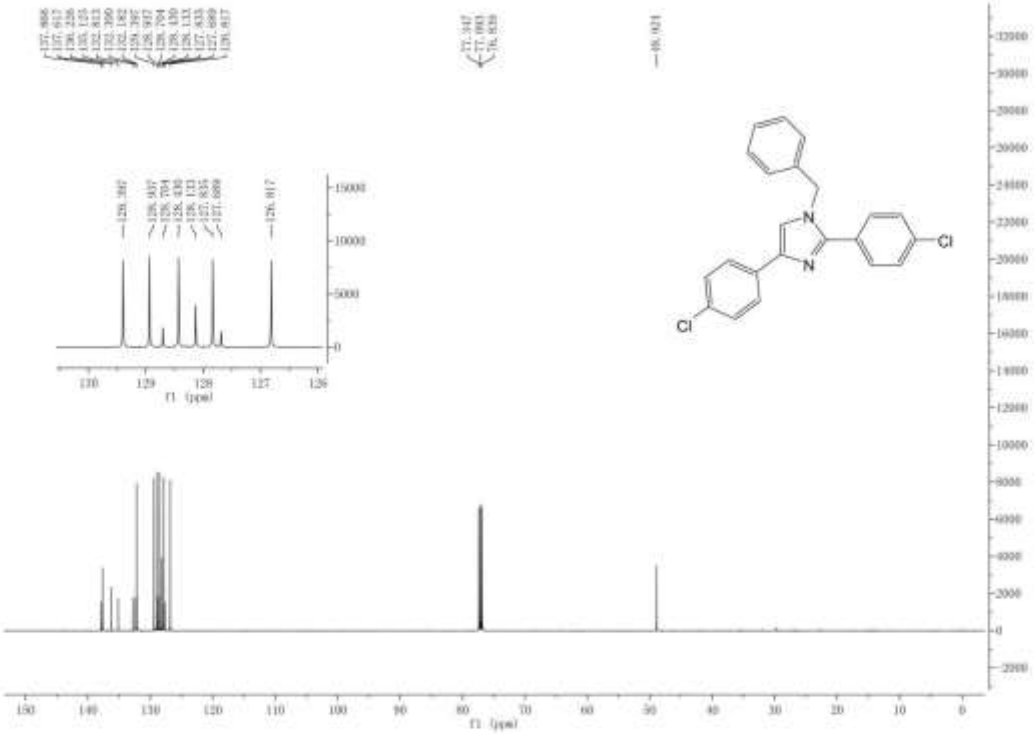

Figure S35. ^{13}C NMR spectrum of **16l**

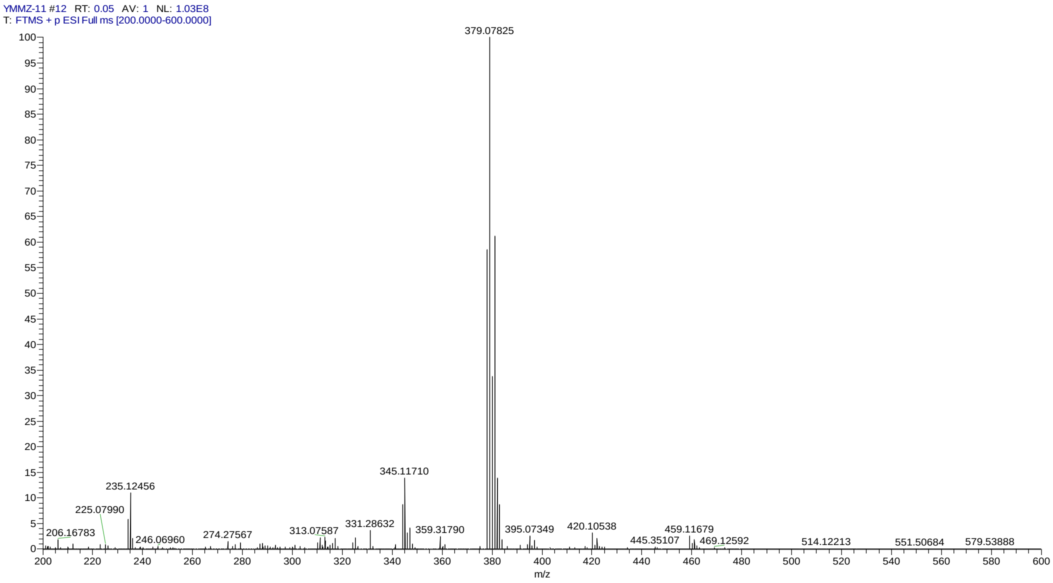

Figure S36. HRMS spectrum of **16l**

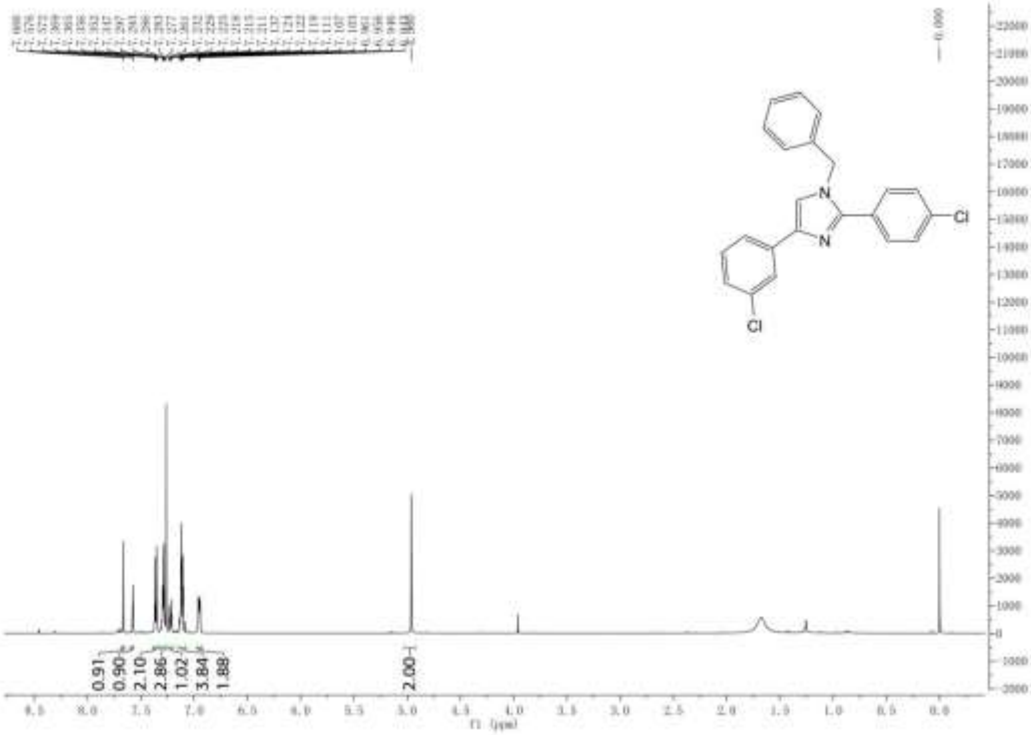

Figure S37. ^{1}H NMR spectrum of **16m**

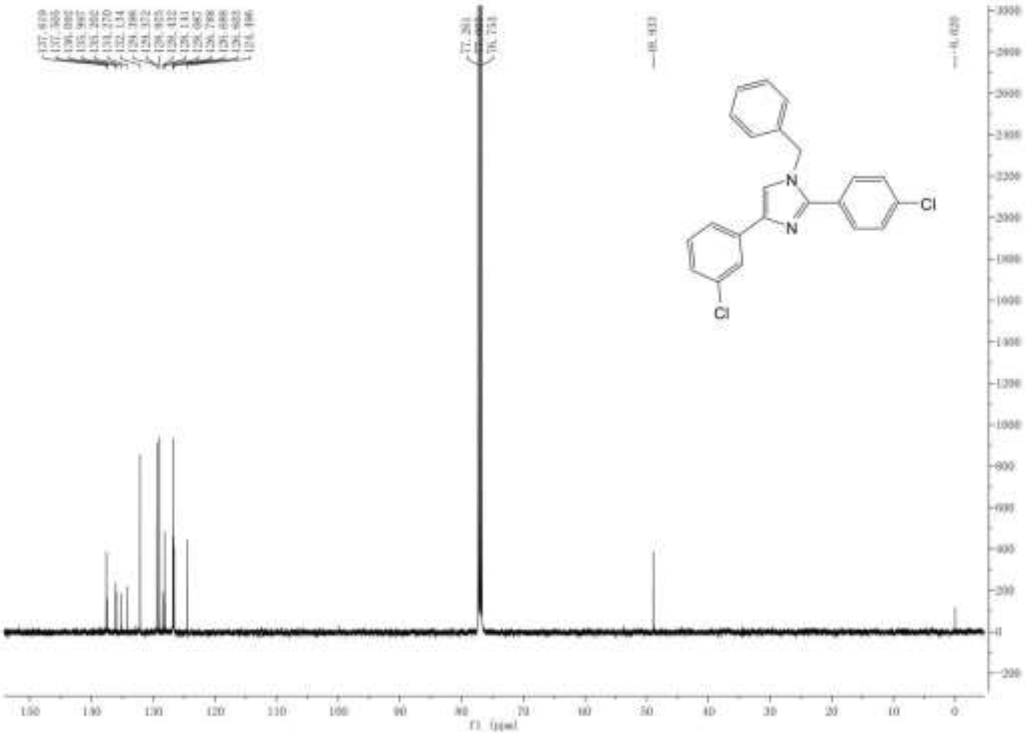

Figure S38. ^{13}C NMR spectrum of **16m**

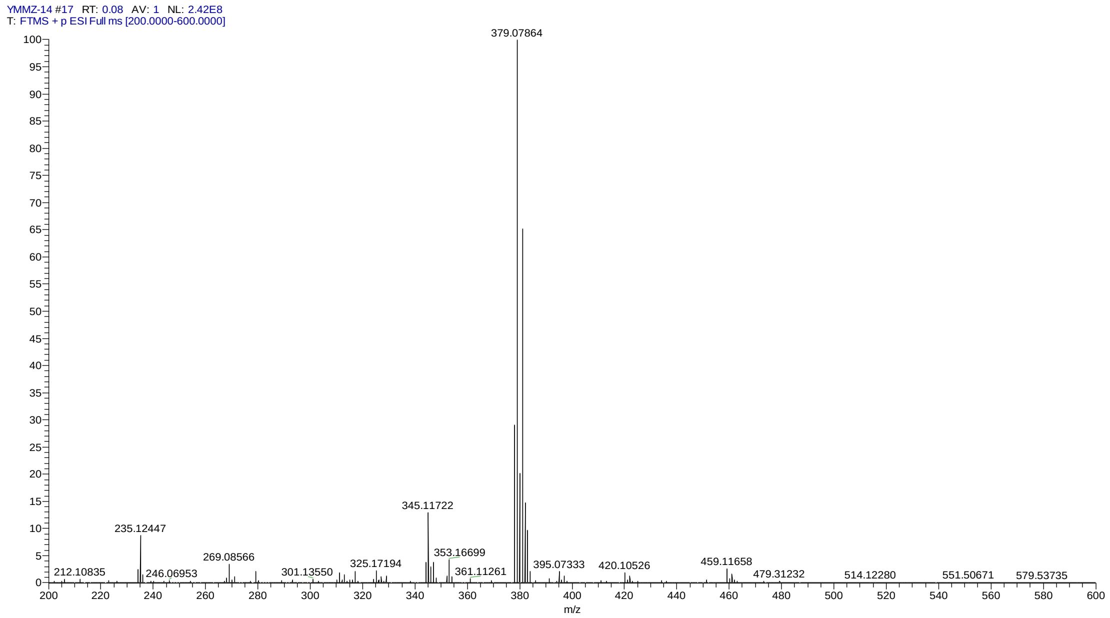

Figure S39. HRMS spectrum of **16m**

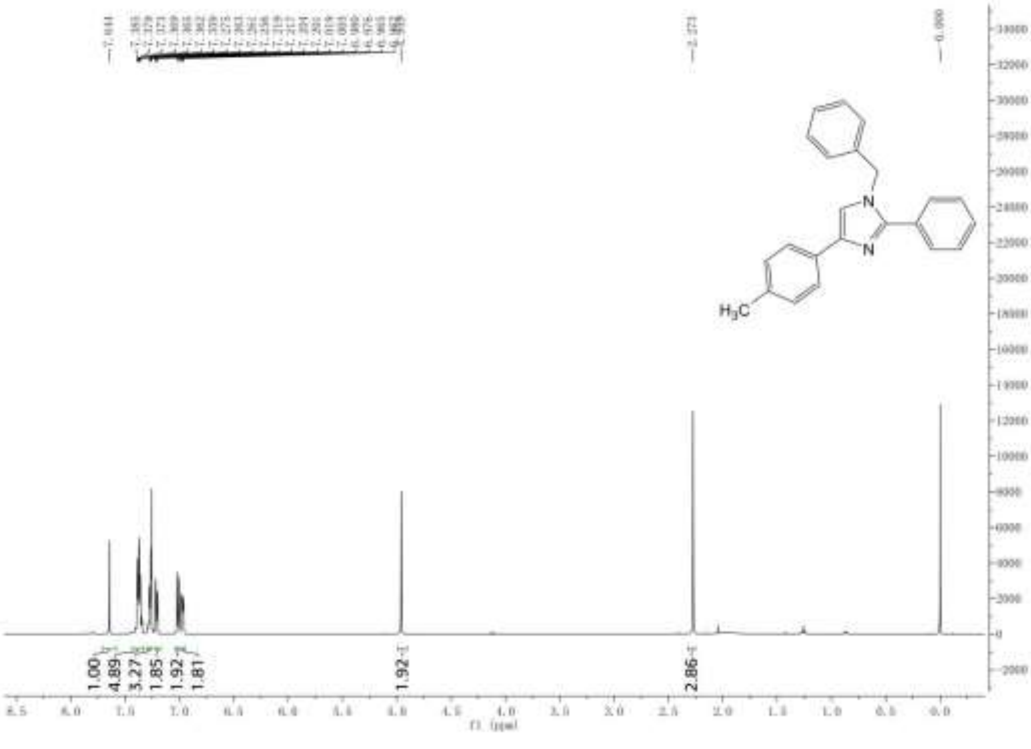

Figure S40. ^{1}H NMR spectrum of **16n**

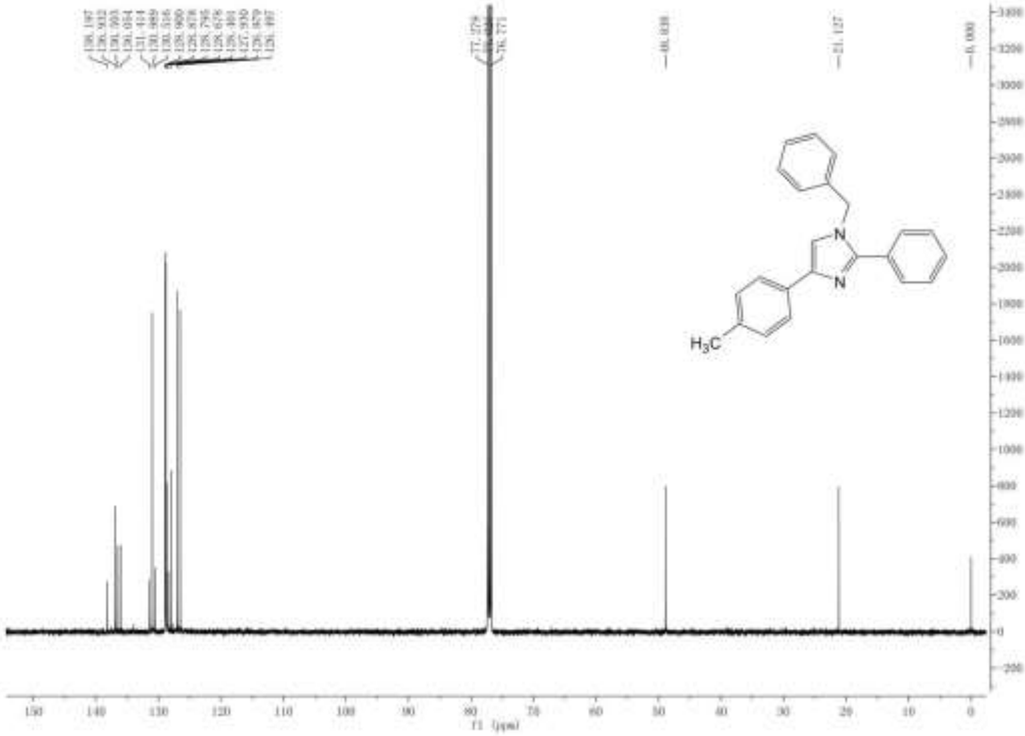

Figure S41. ^{13}C NMR spectrum of **16n**

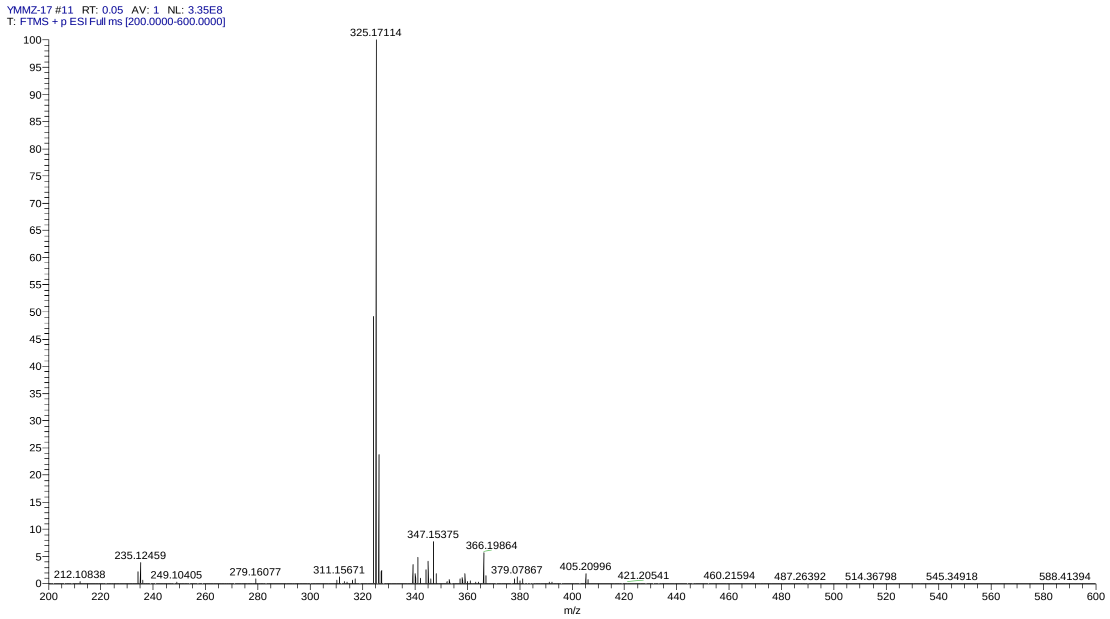

Figure S42. HRMS spectrum of **16n**

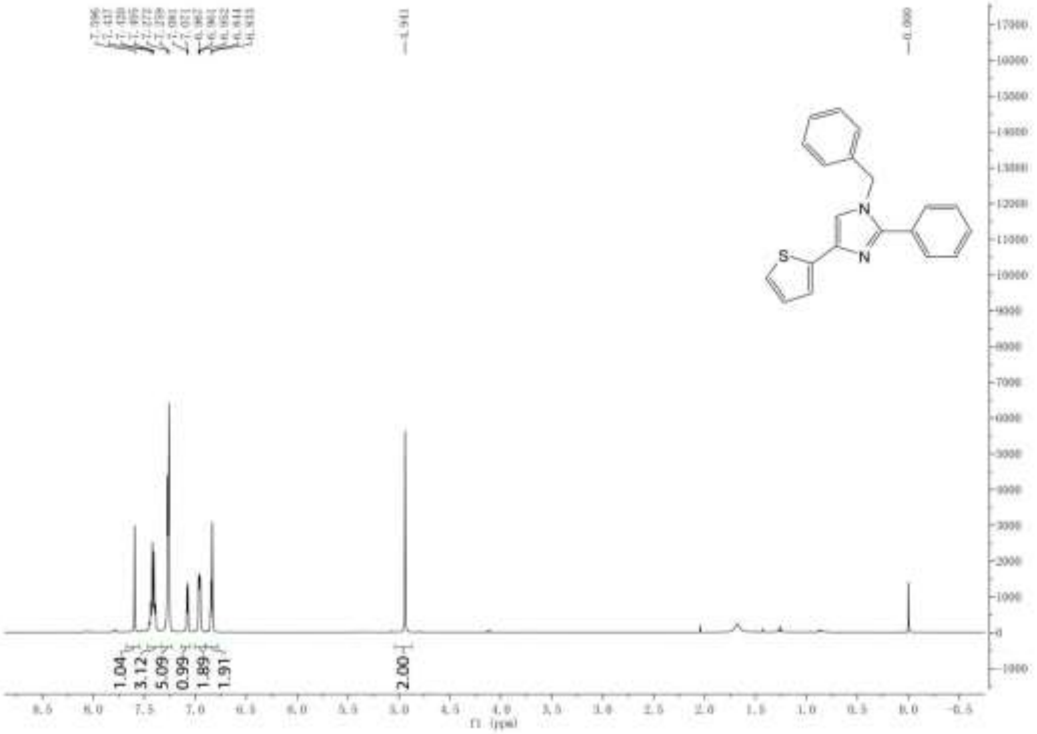

Figure S43. ^{1}H NMR spectrum of **16o**

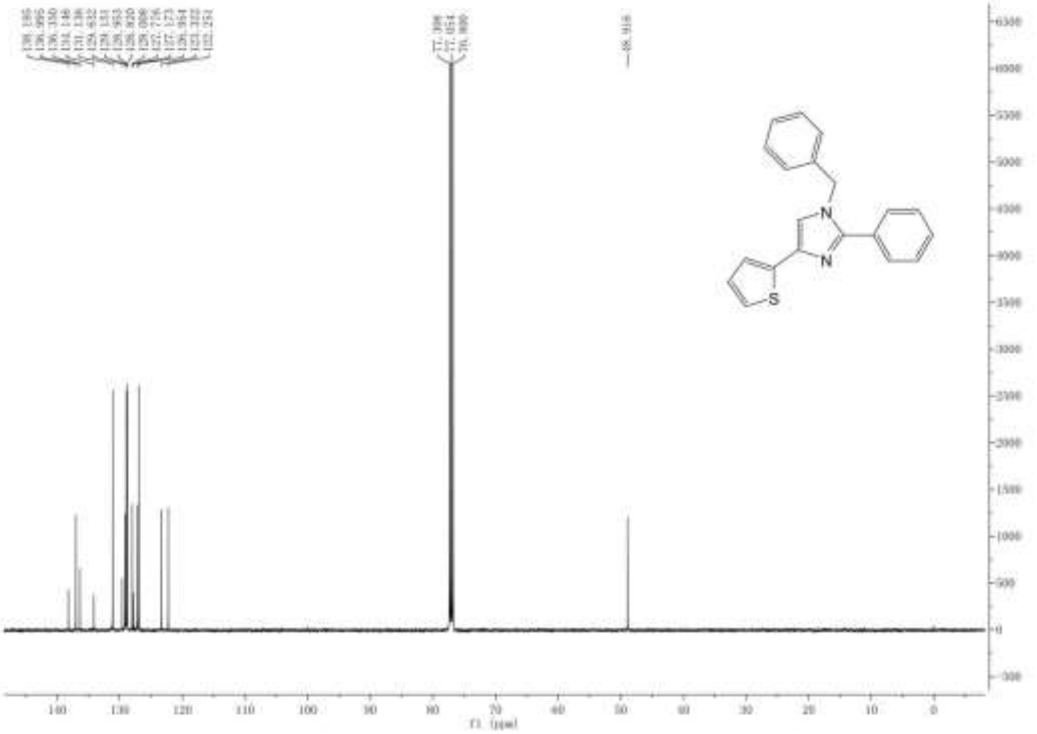

Figure S44. ^{13}C NMR spectrum of **16o**

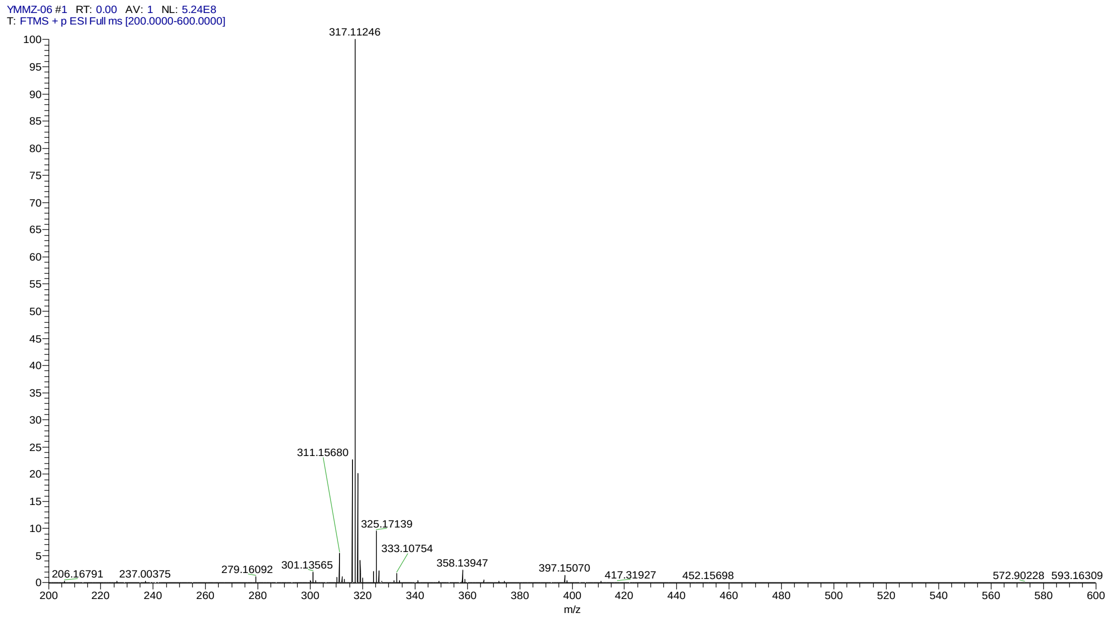

Figure S45. HRMS spectrum of **16o**

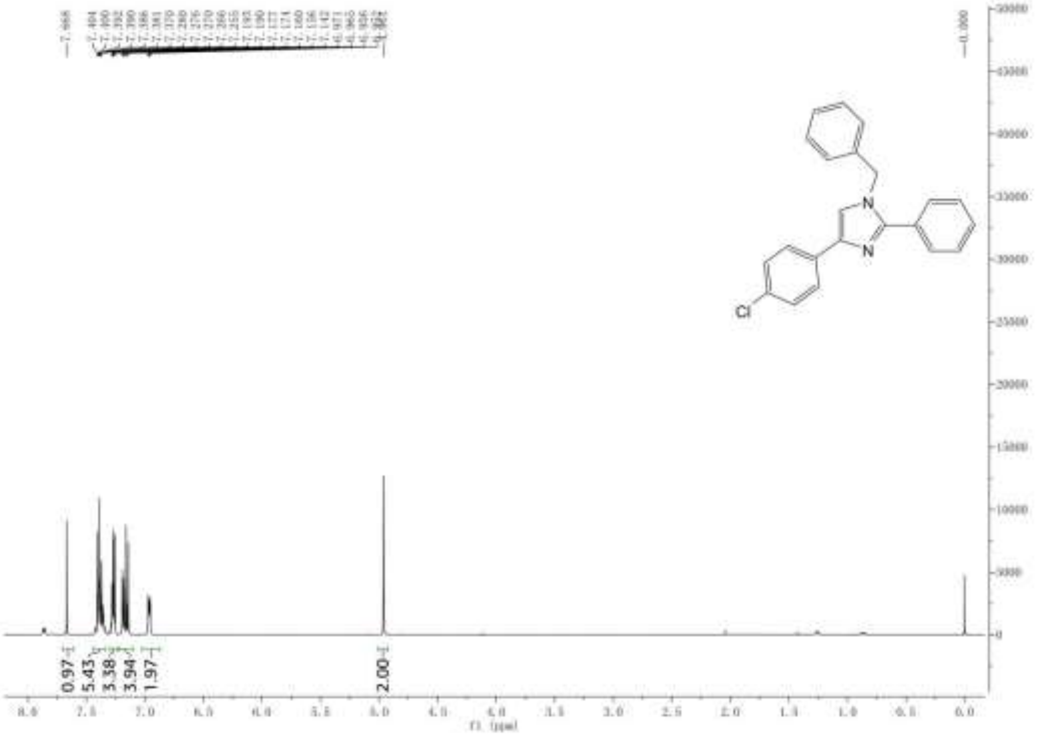

Figure S46. ^{1}H NMR spectrum of **16p**

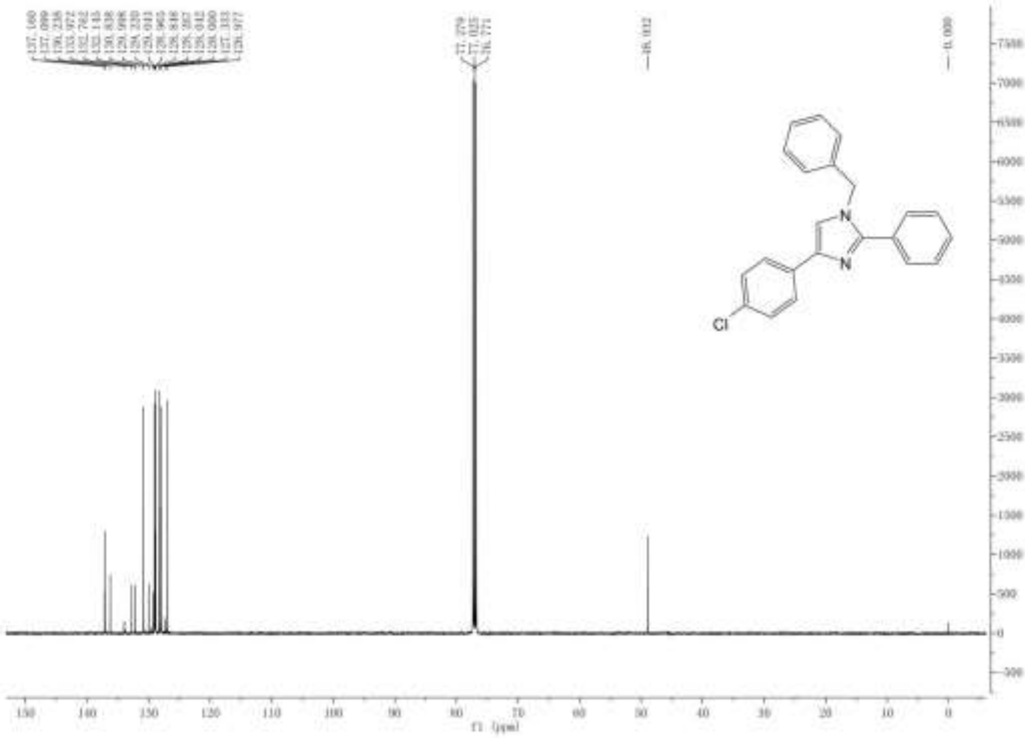

Figure S47. ^{13}C NMR spectrum of **16p**

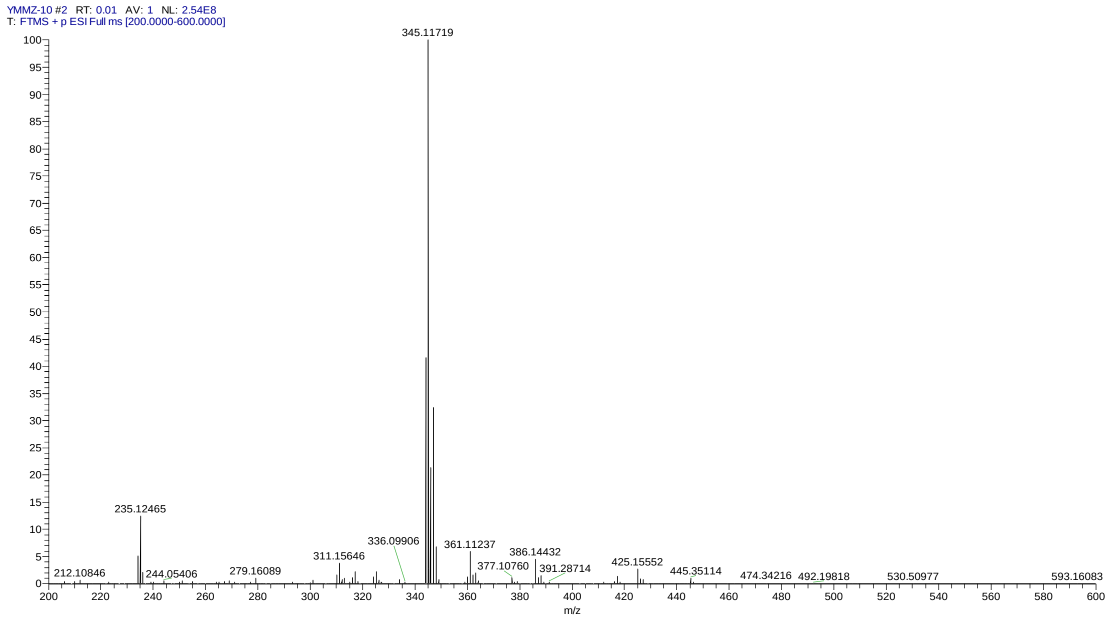

Figure S48. HRMS spectrum of **16p**

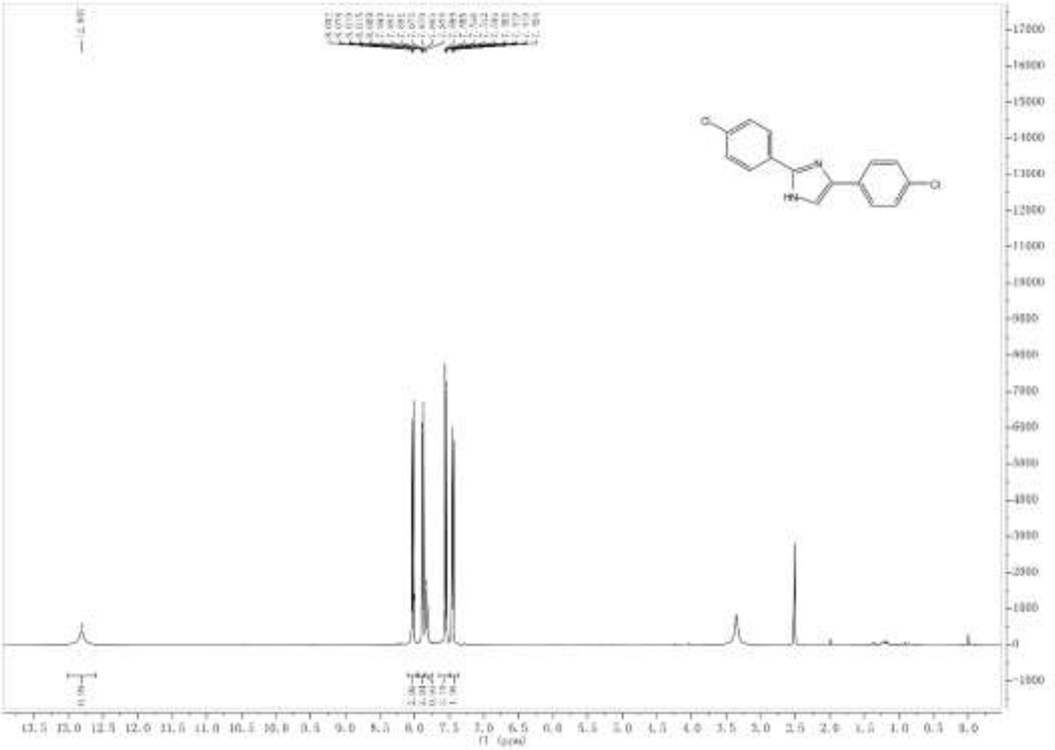

Figure S49. ^{1}H NMR spectrum of **19**

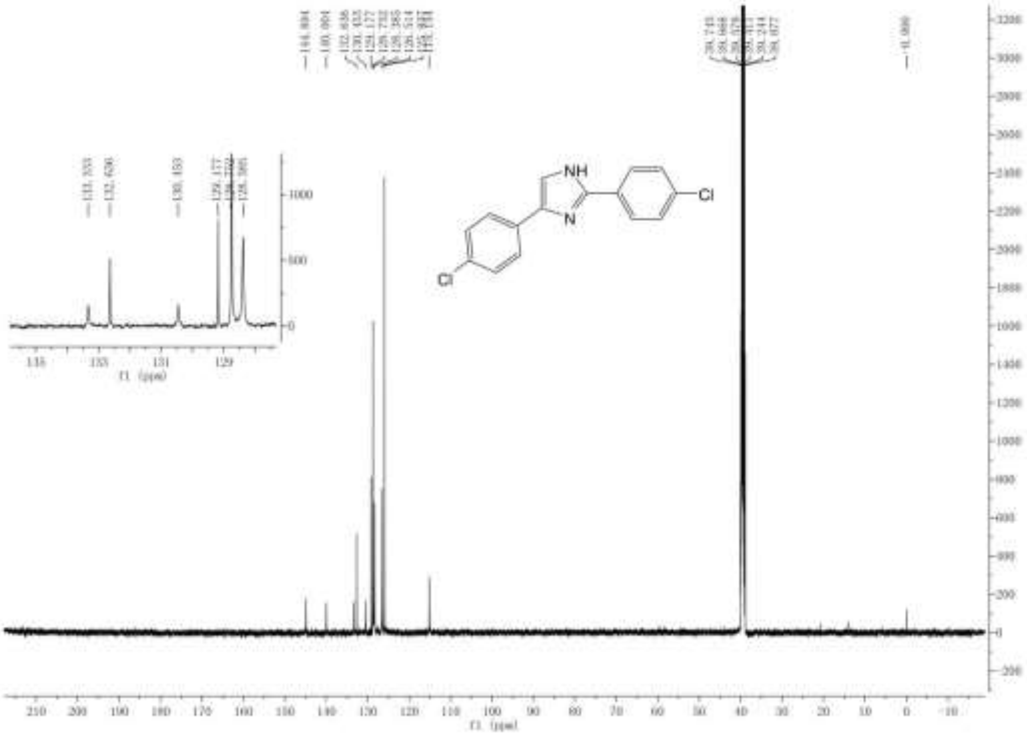

Figure S50. ^{13}C NMR spectrum of **19**

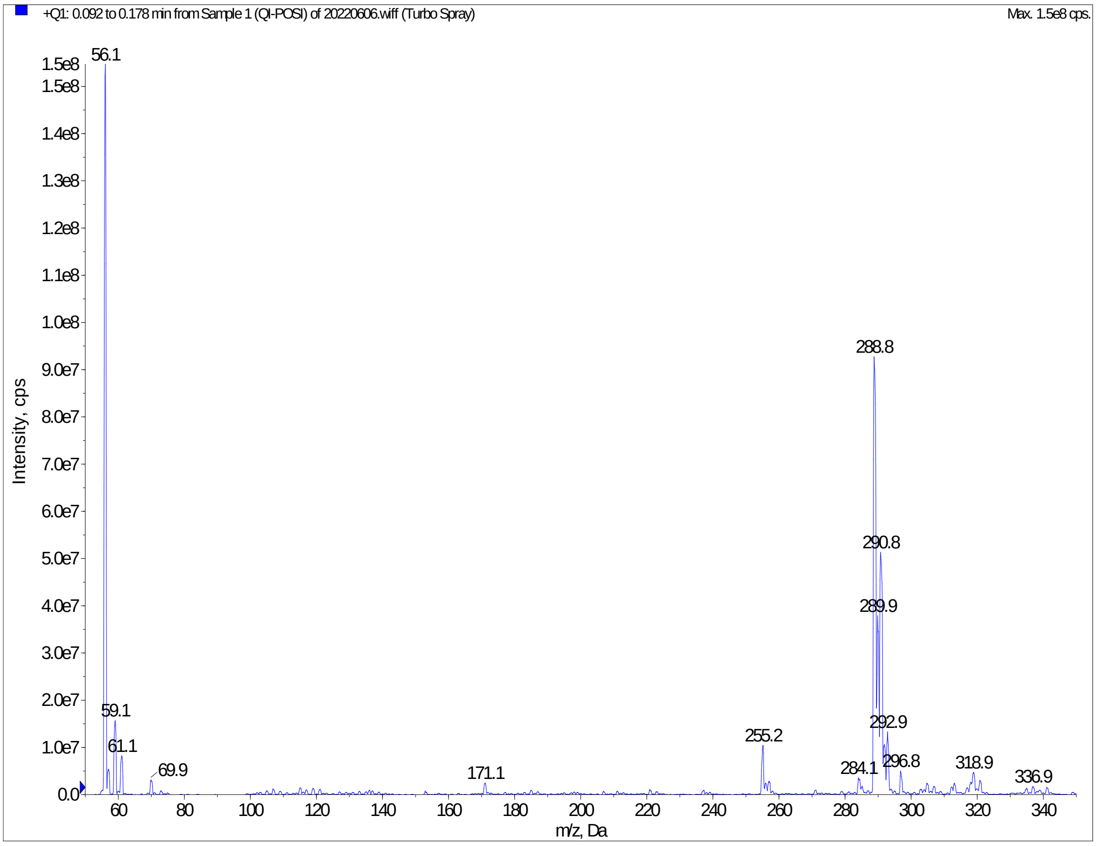

Figure S51. HRMS spectrum of **19**

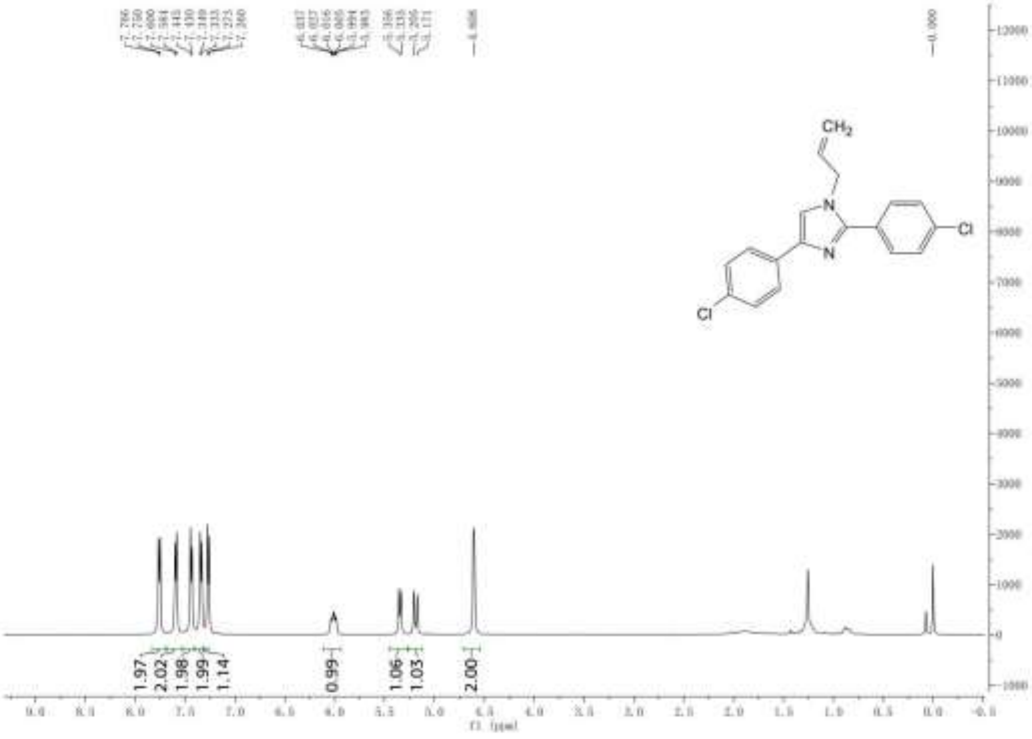

Figure S52. ^{1}H NMR spectrum of **20a**

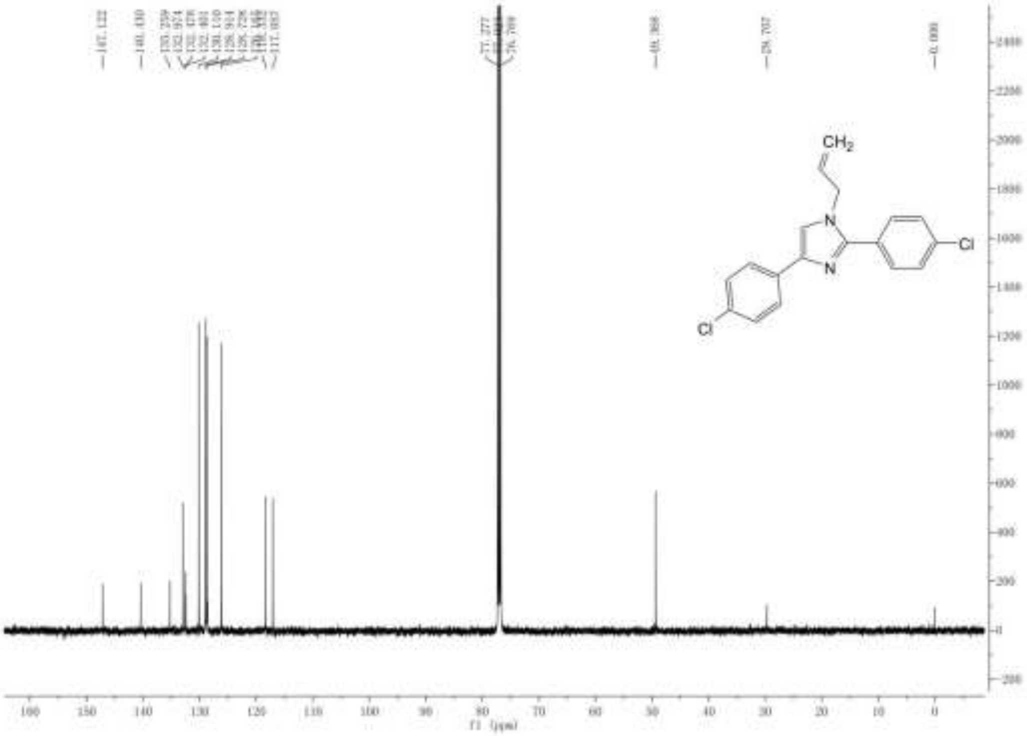

Figure S53. ^{13}C NMR spectrum of **20a**

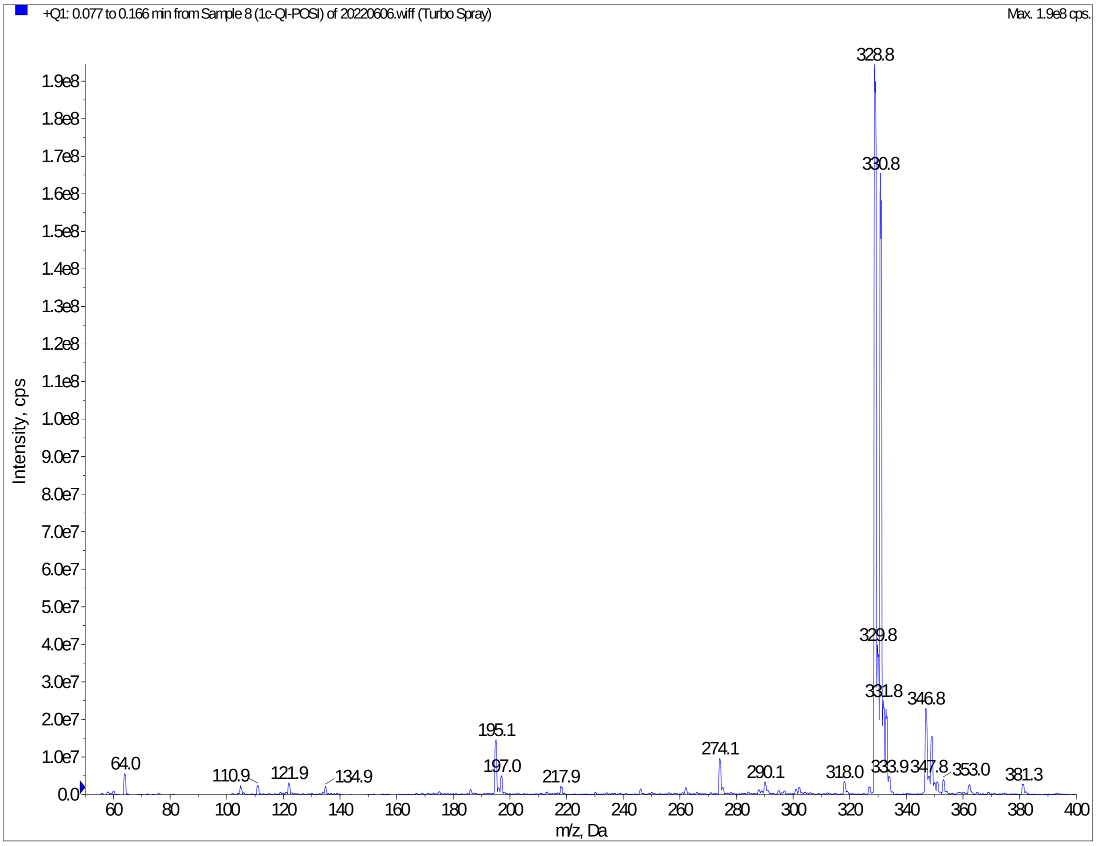

Figure S54. HRMS spectrum of **20a**

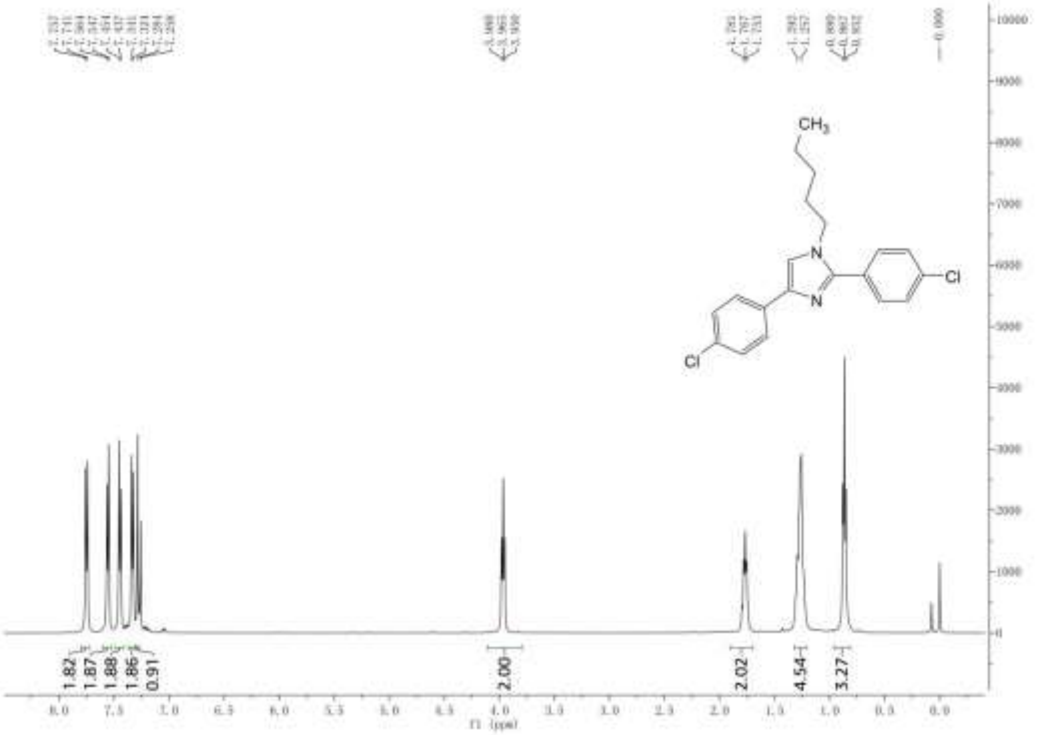

Figure S55. ^{1}H NMR spectrum of **20b**

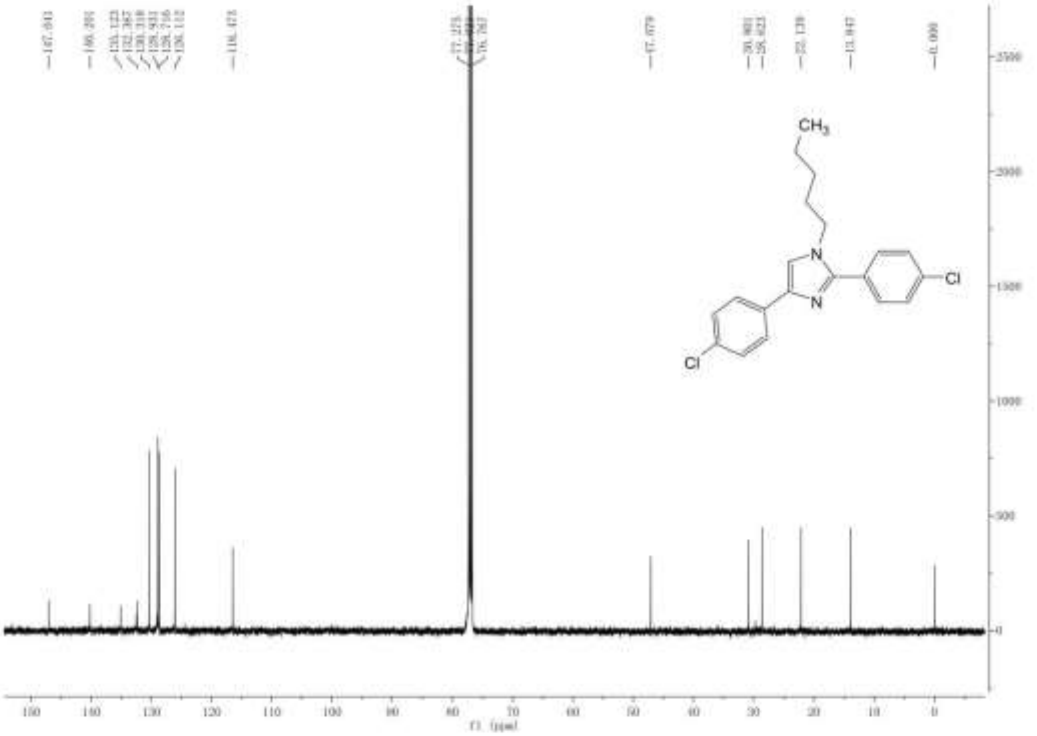

Figure S56. ^{13}C NMR spectrum of **20b**

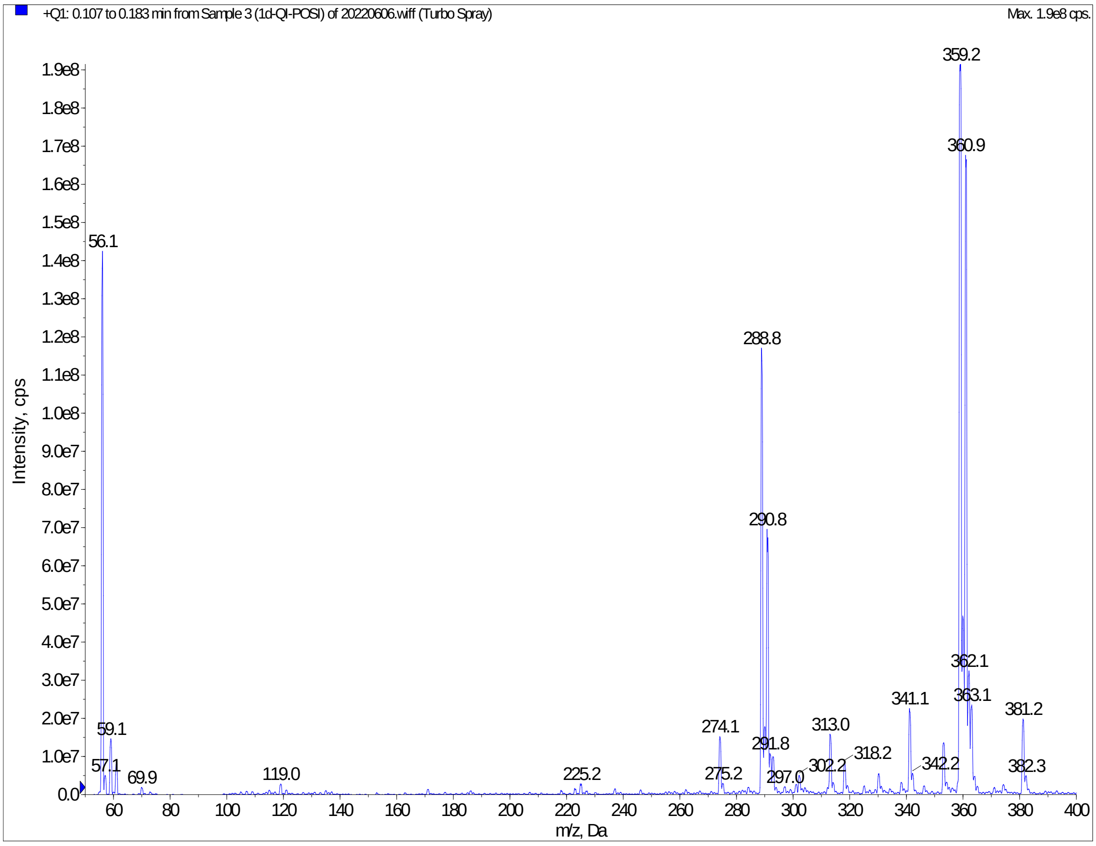

Figure S57. HRMS spectrum of **20b**

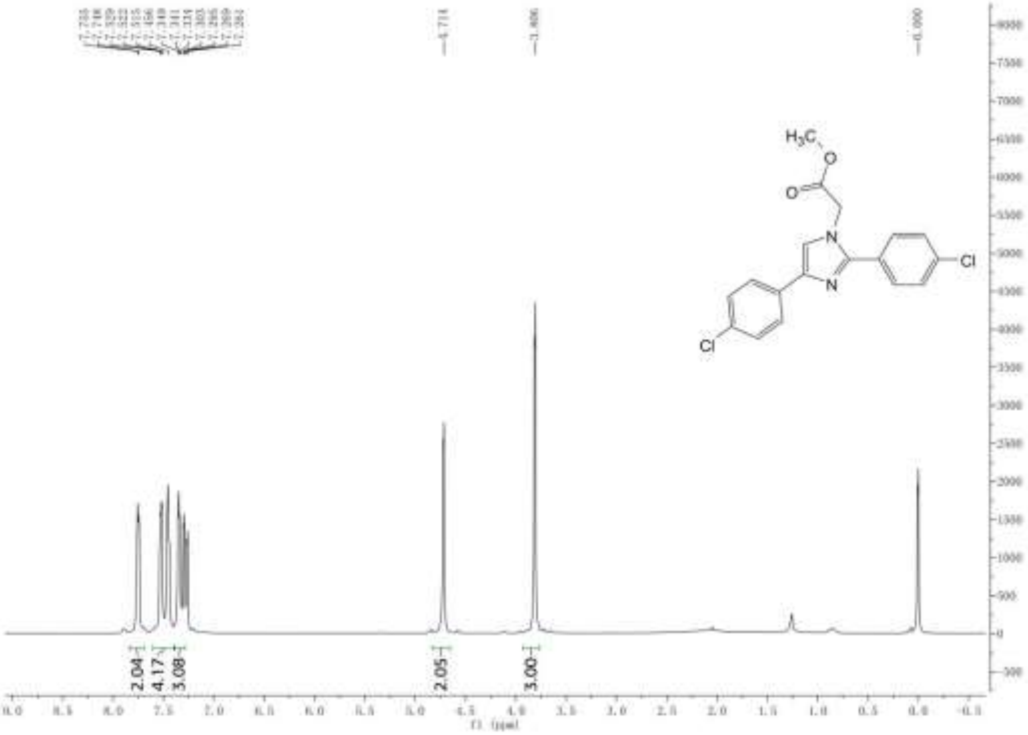

Figure S58. ^{1}H NMR spectrum of **20c**

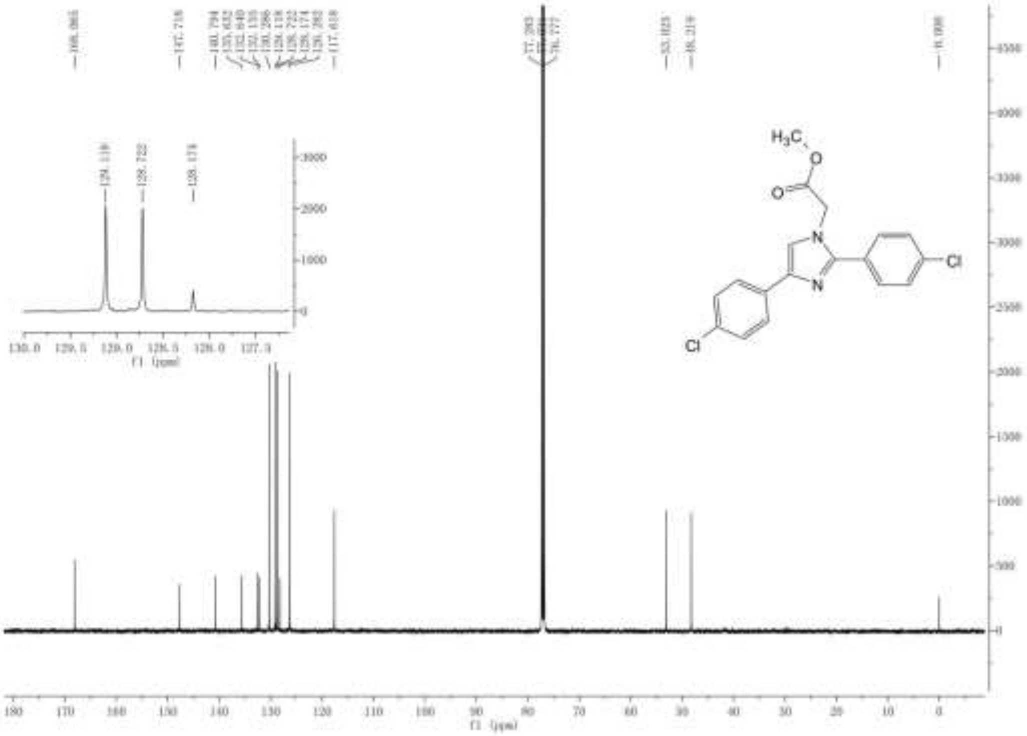

Figure S59. ^{13}C NMR spectrum of **20c**

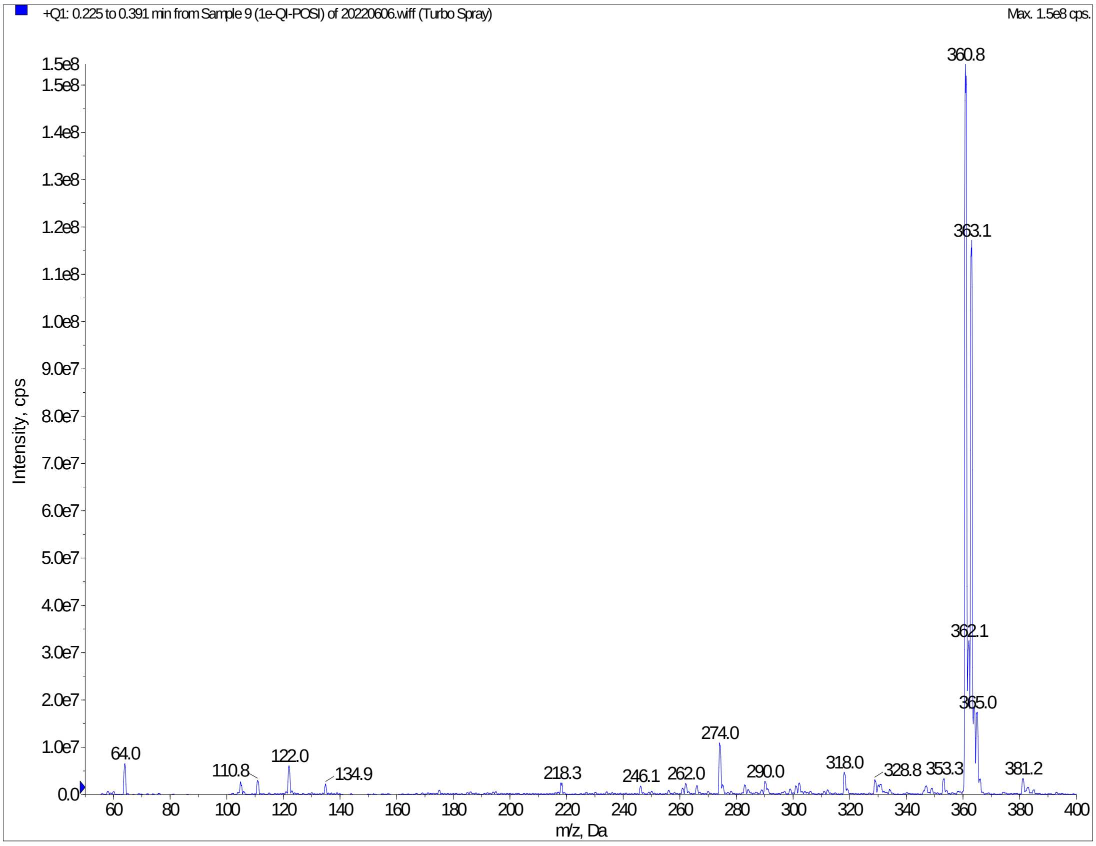

Figure S60. HRMS spectrum of **20c**

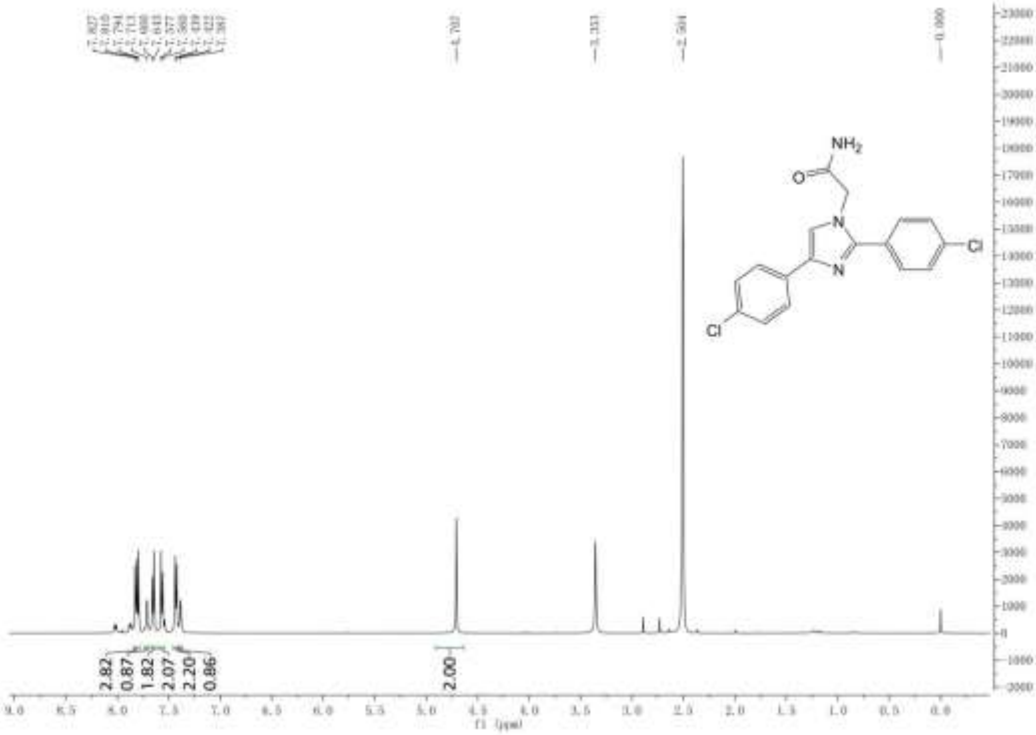

Figure S61. ^{1}H NMR spectrum of **20d**

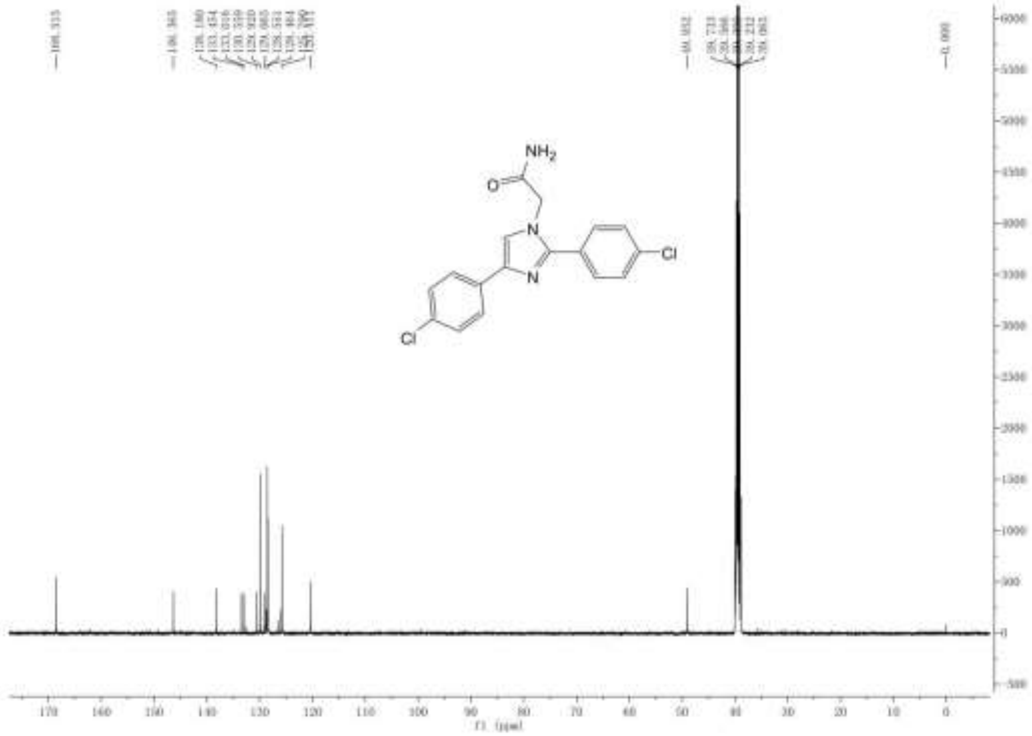

Figure S62. ^{13}C NMR spectrum of **20d**

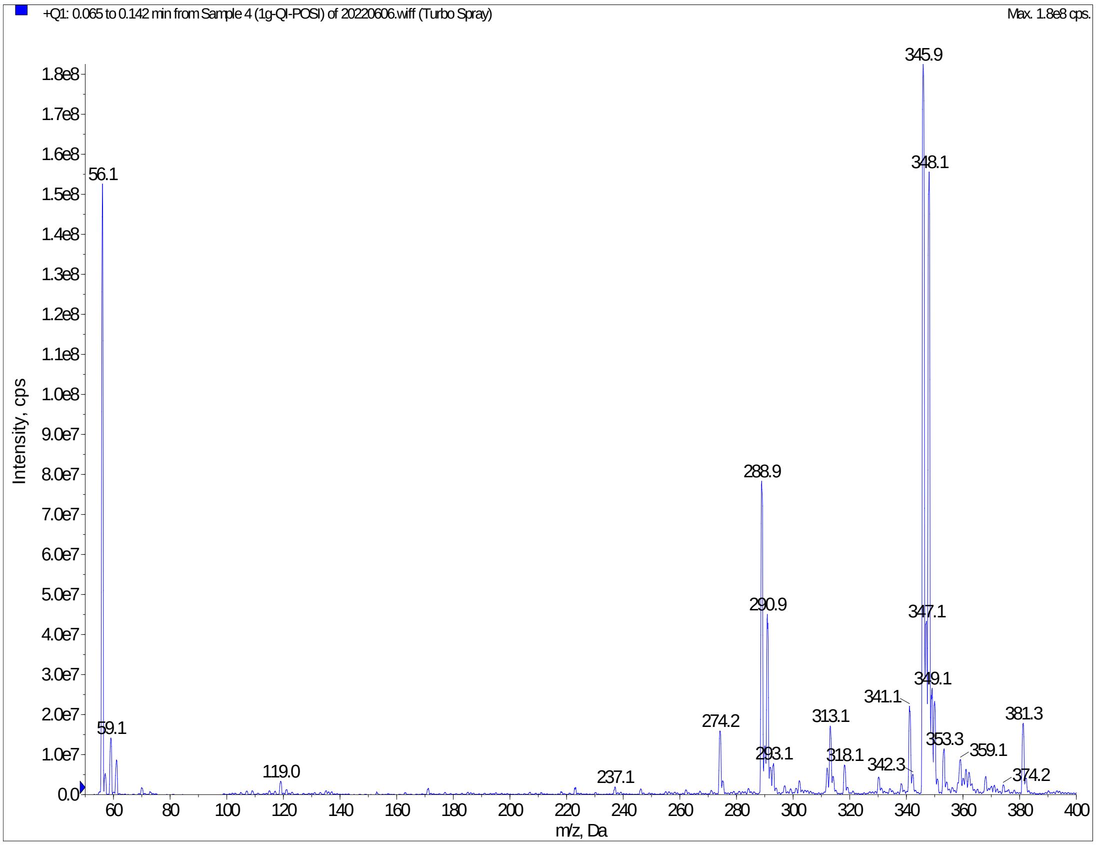

Figure S63. HRMS spectrum of **20d**

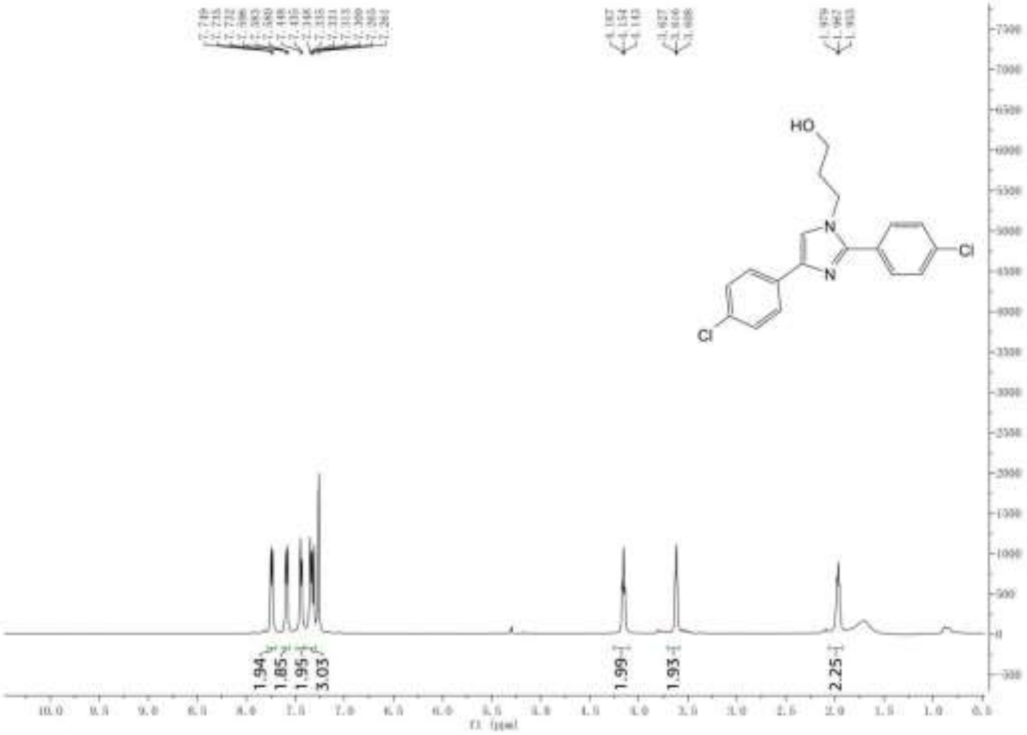

Figure S64. ^{1}H NMR spectrum of **20e**

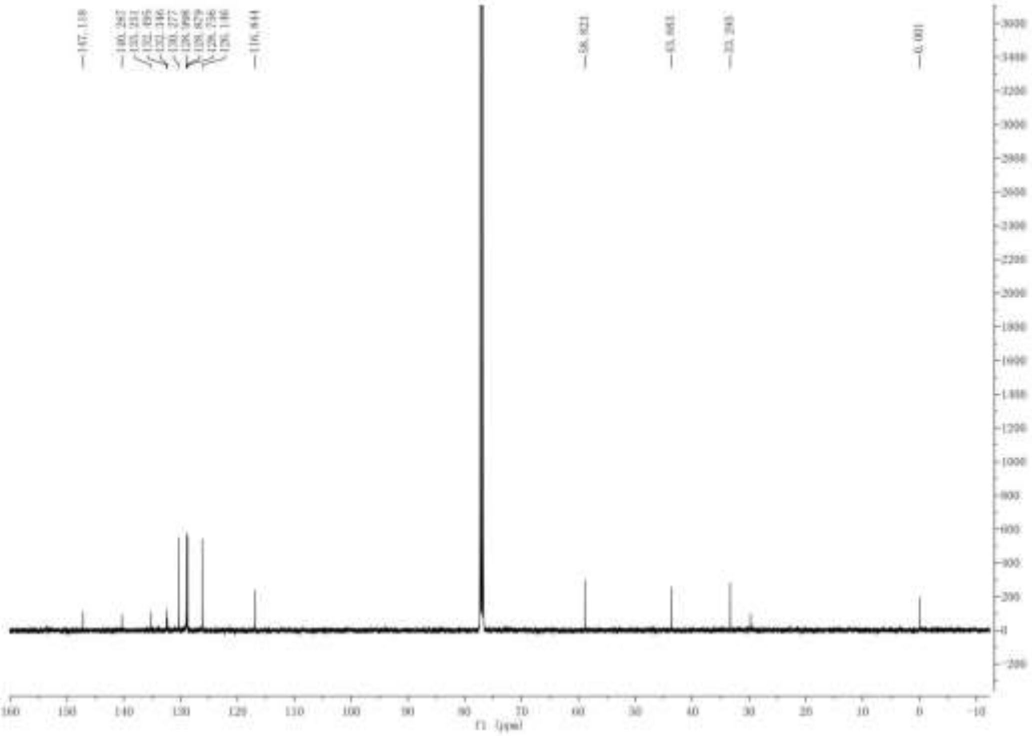

Figure S65. ^{13}C NMR spectrum of **20e**

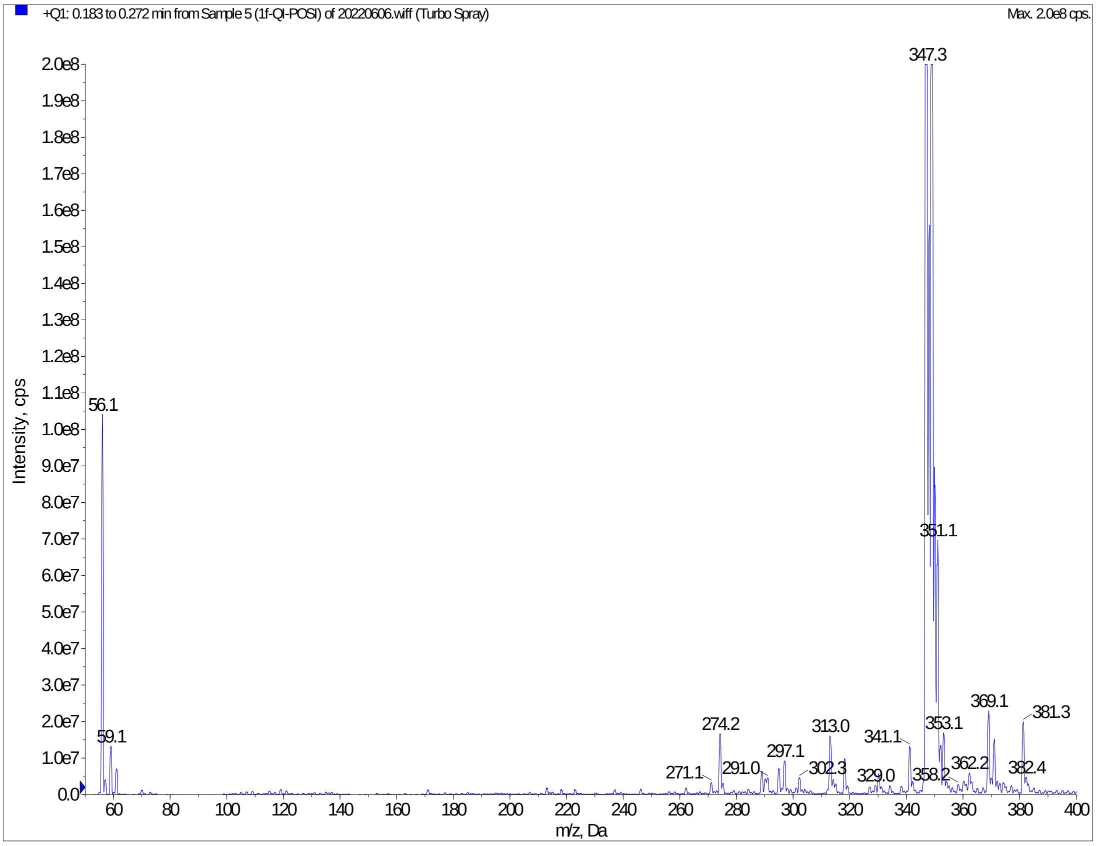

Figure S66. HRMS spectrum of **20e**

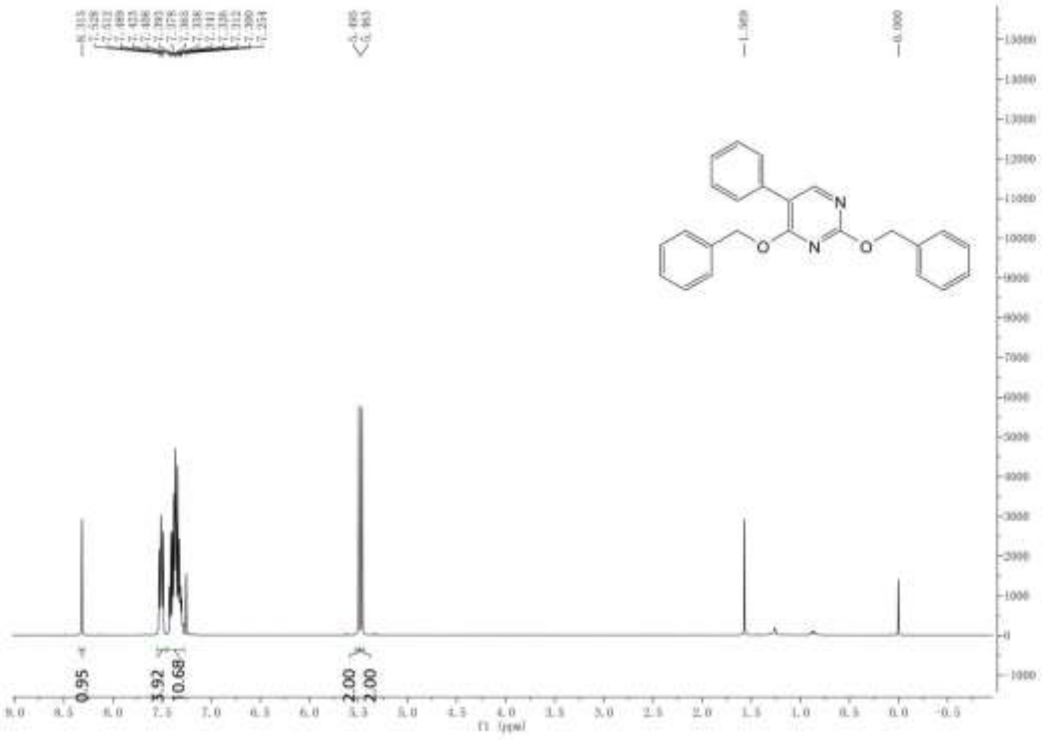

Figure S67. ^{1}H NMR spectrum of **22a**

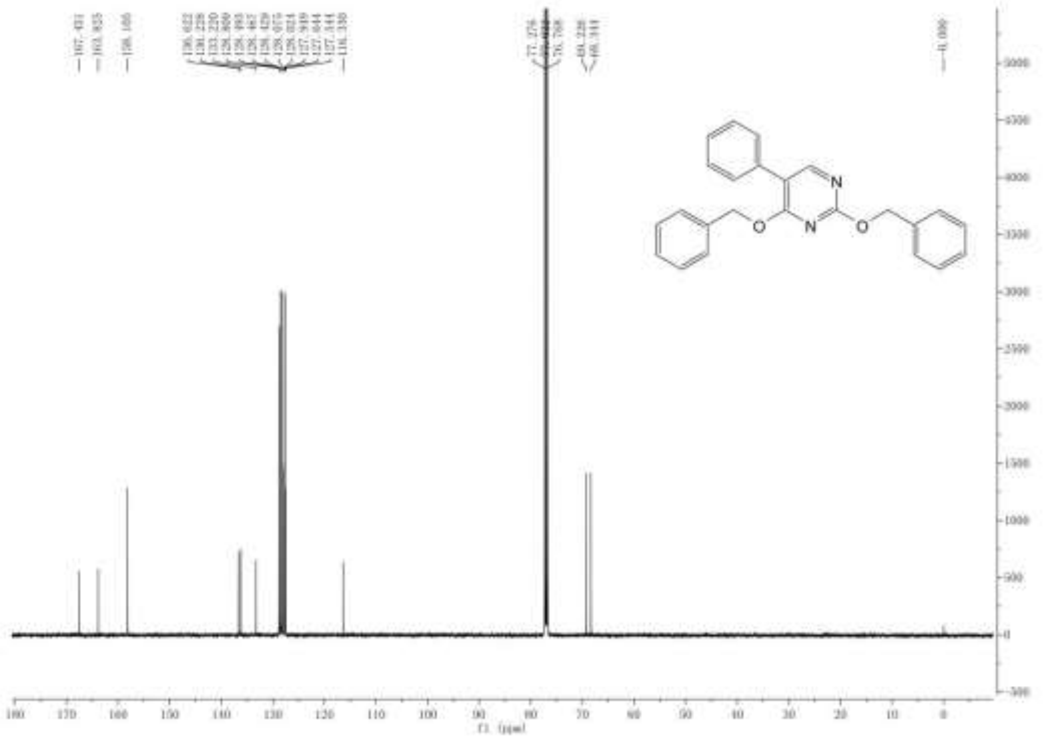

Figure S68. ^{13}C NMR spectrum of **22a**

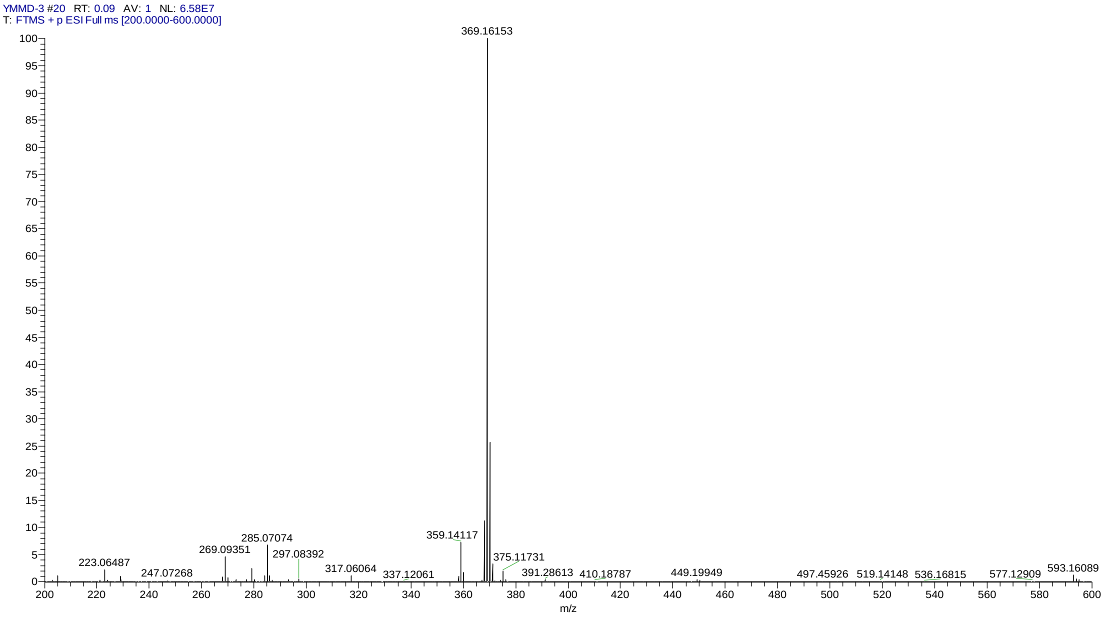

Figure S69. HRMS spectrum of **22a**

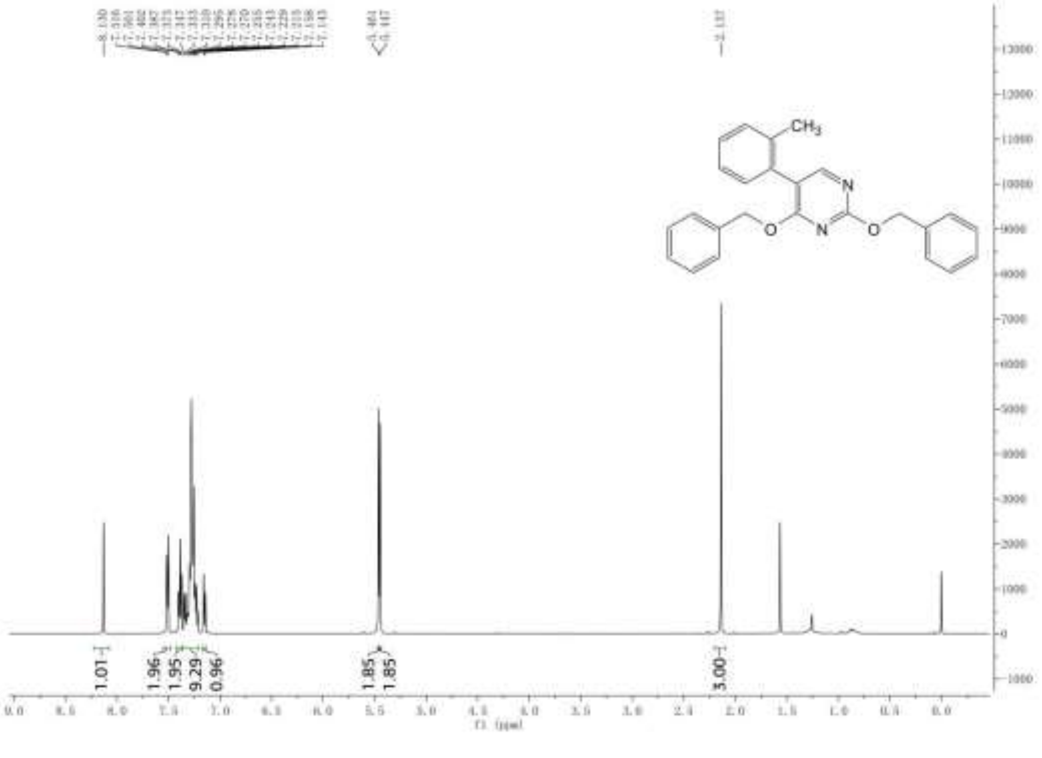

Figure S70. ^{1}H NMR spectrum of **22b**

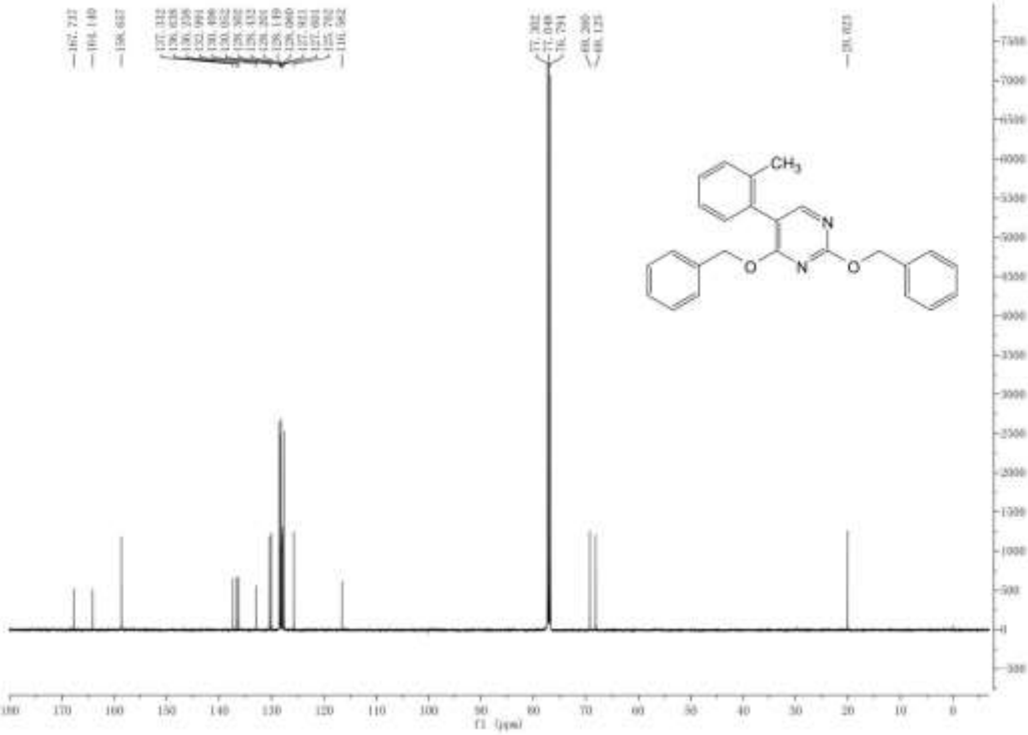

Figure S71. ^{13}C NMR spectrum of **22b**

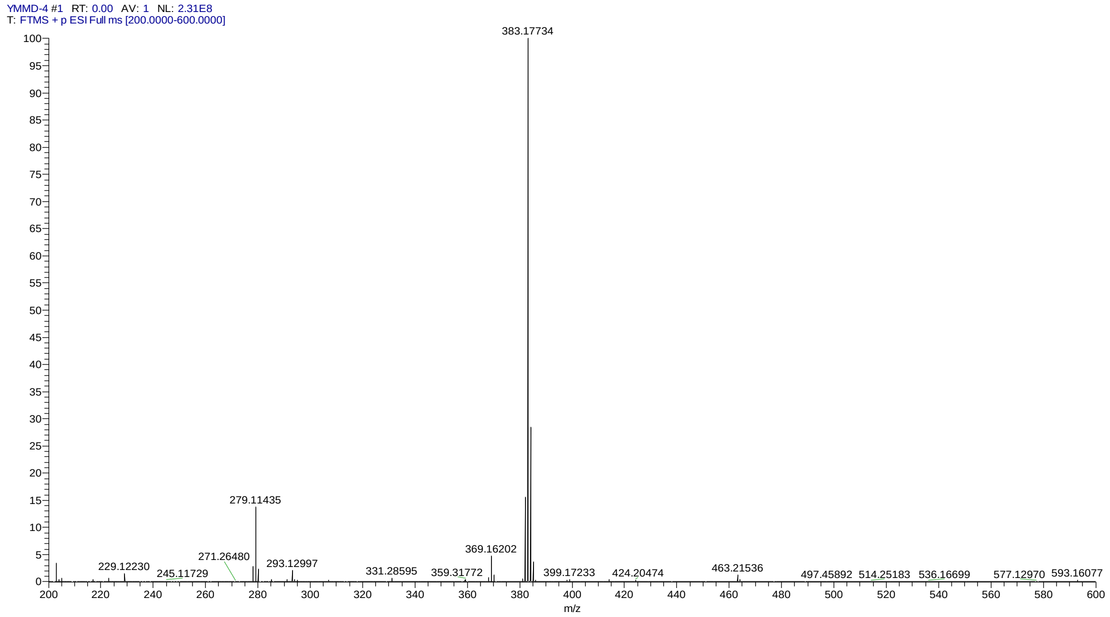

Figure S72. HRMS spectrum of **22b**

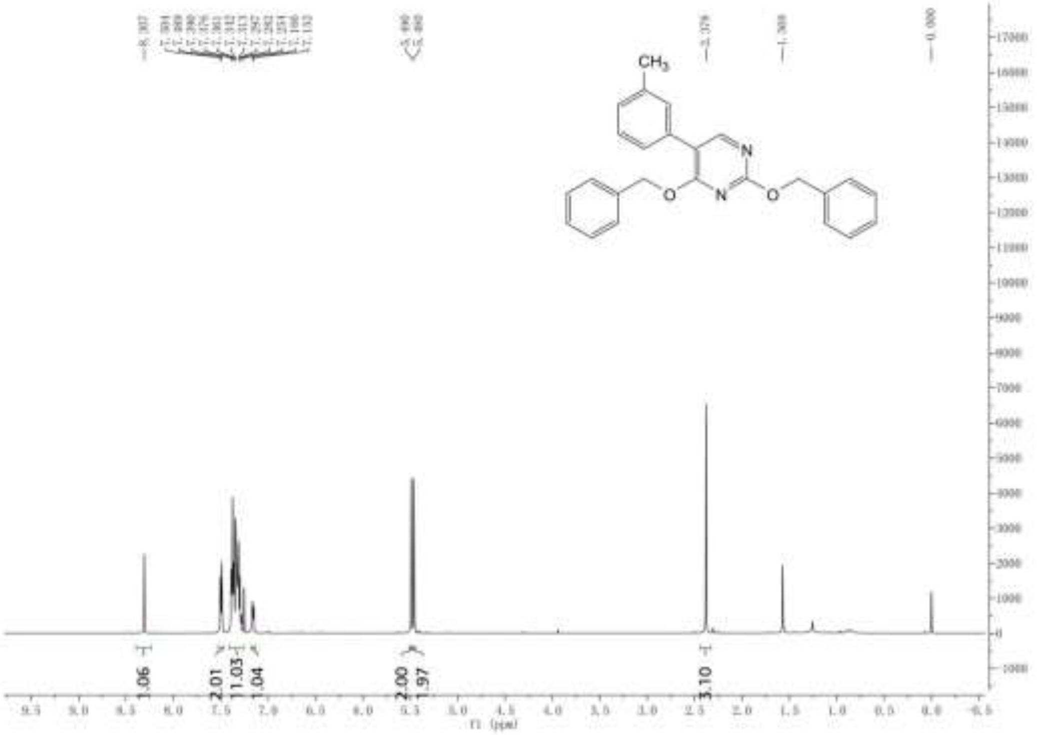

Figure S73. ^{1}H NMR spectrum of **22c**

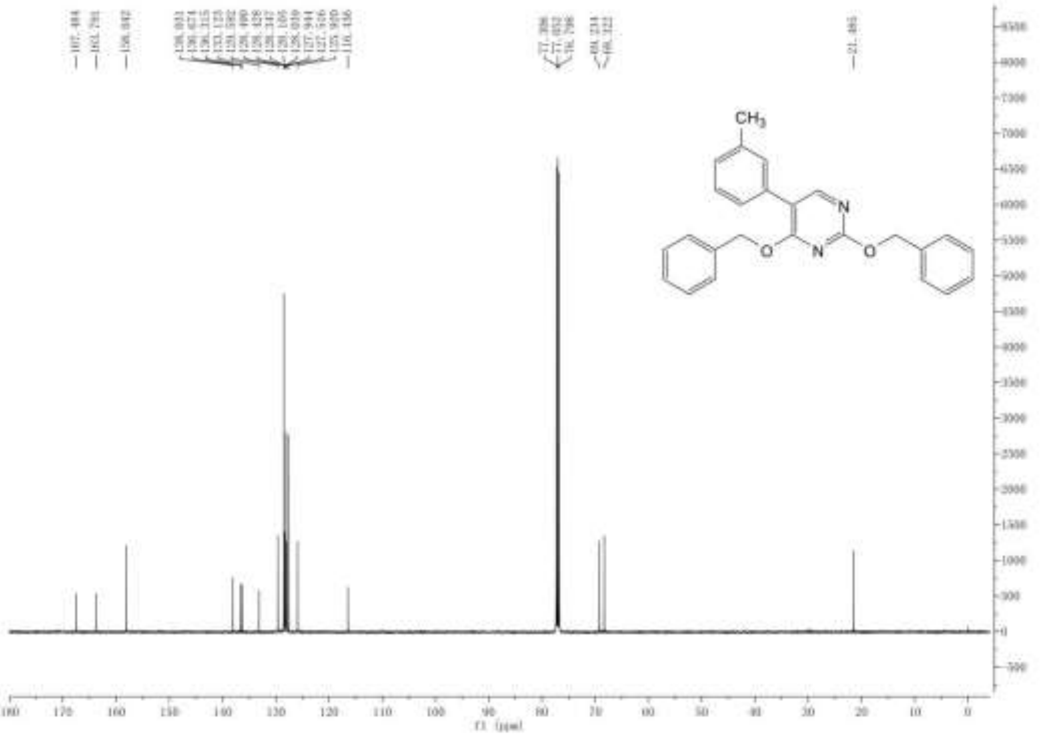

Figure S74. ^{13}C NMR spectrum of **22c**

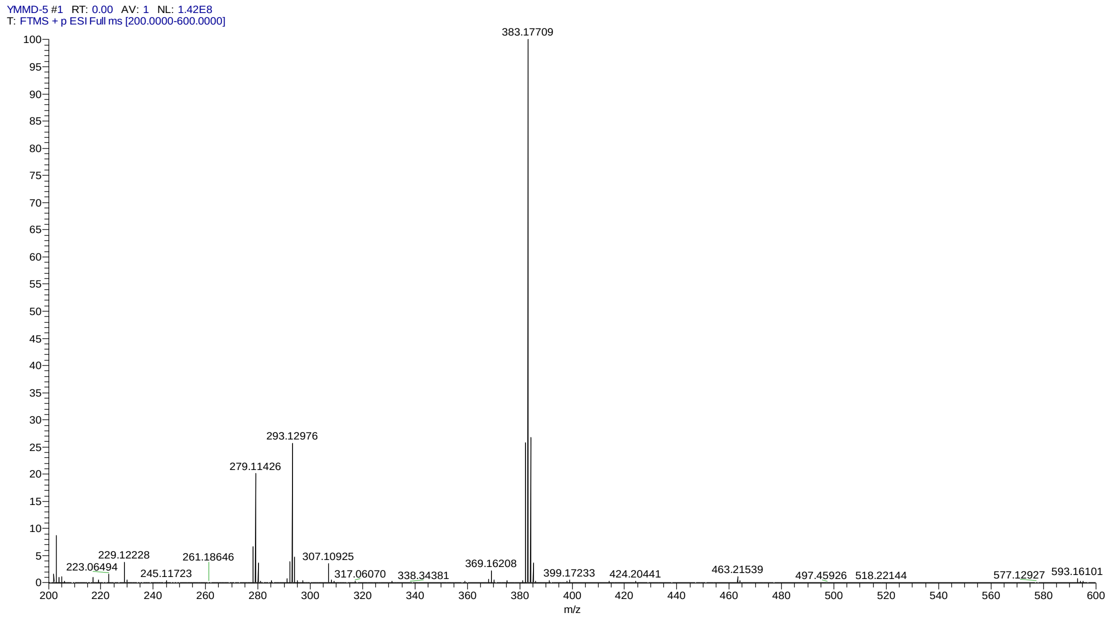

Figure S75. HRMS spectrum of **22c**

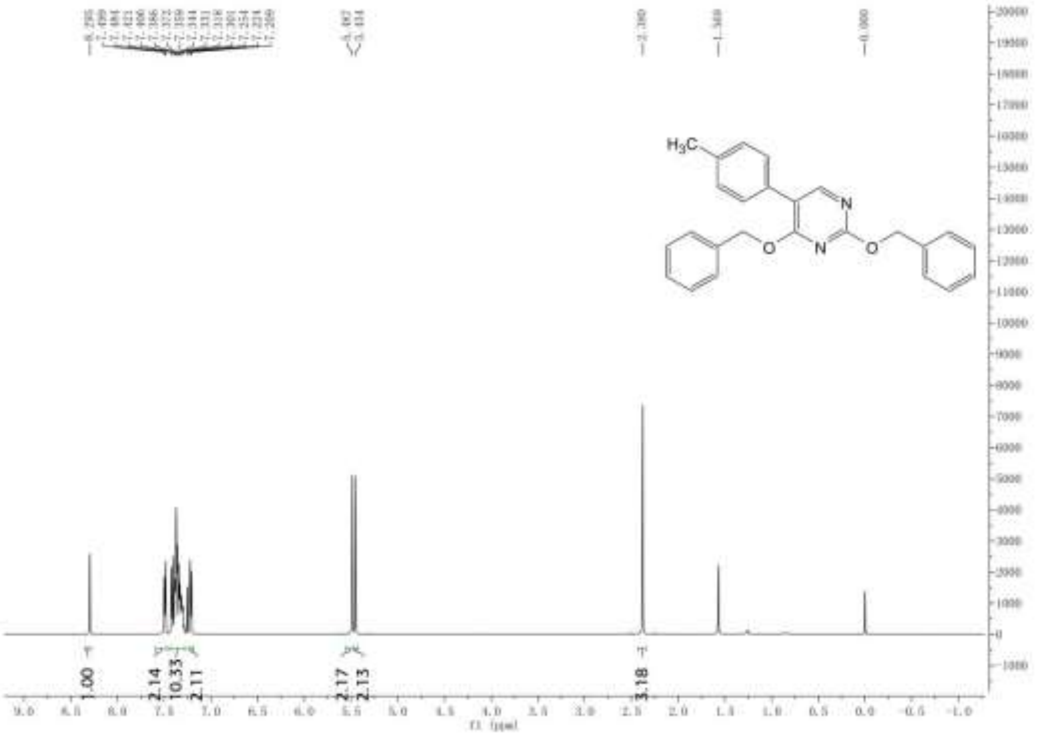

Figure S76. ^{1}H NMR spectrum of **22d**

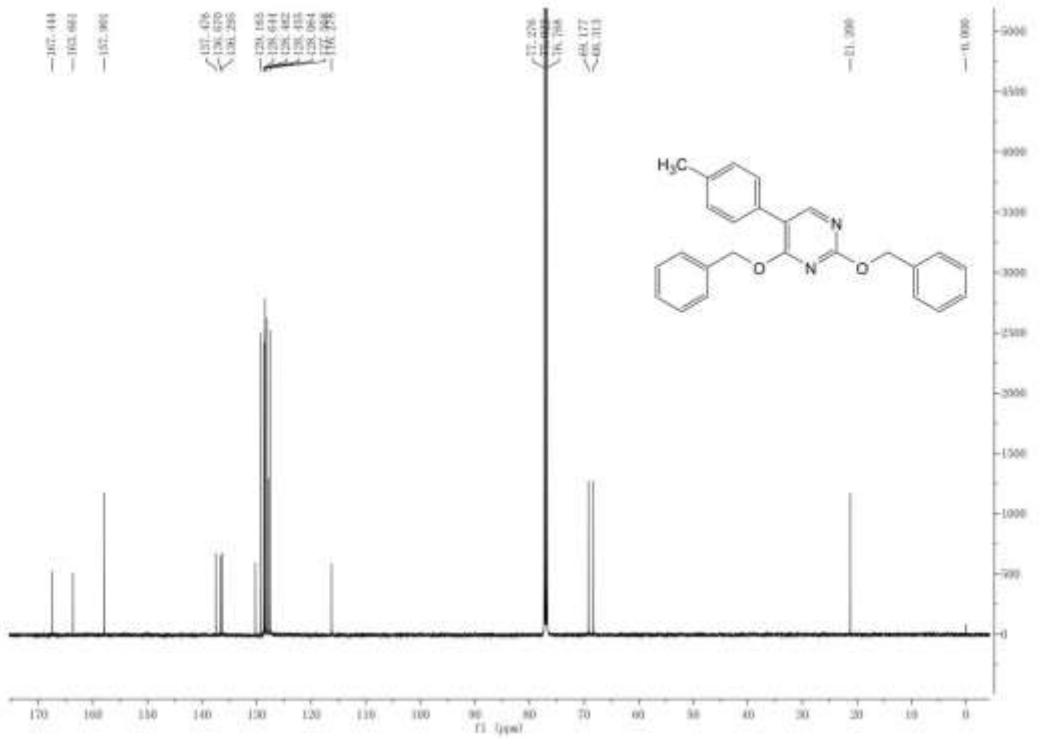

Figure S77. ^{13}C NMR spectrum of **22d**

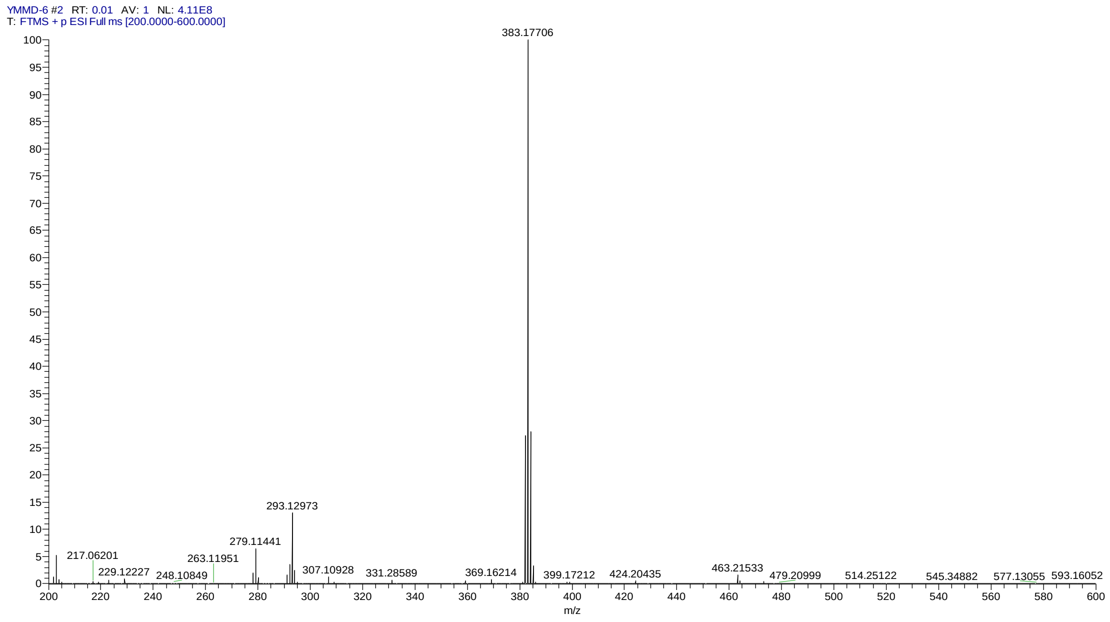

Figure S78. HRMS spectrum of **22d**

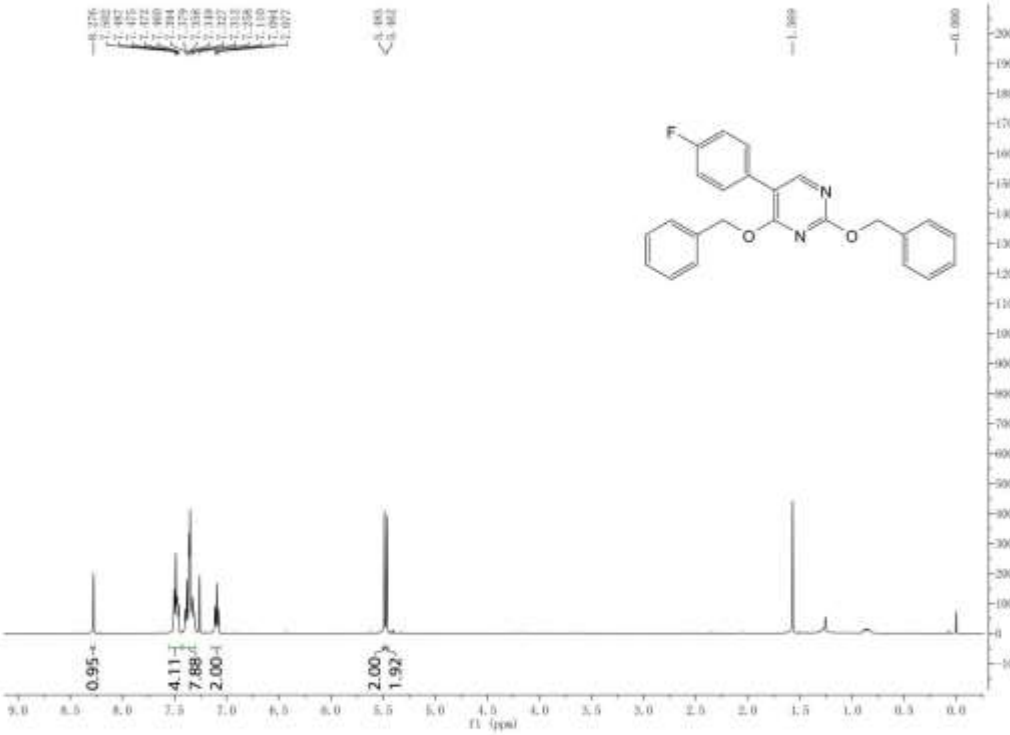

Figure S79. ^{1}H NMR spectrum of **22e**

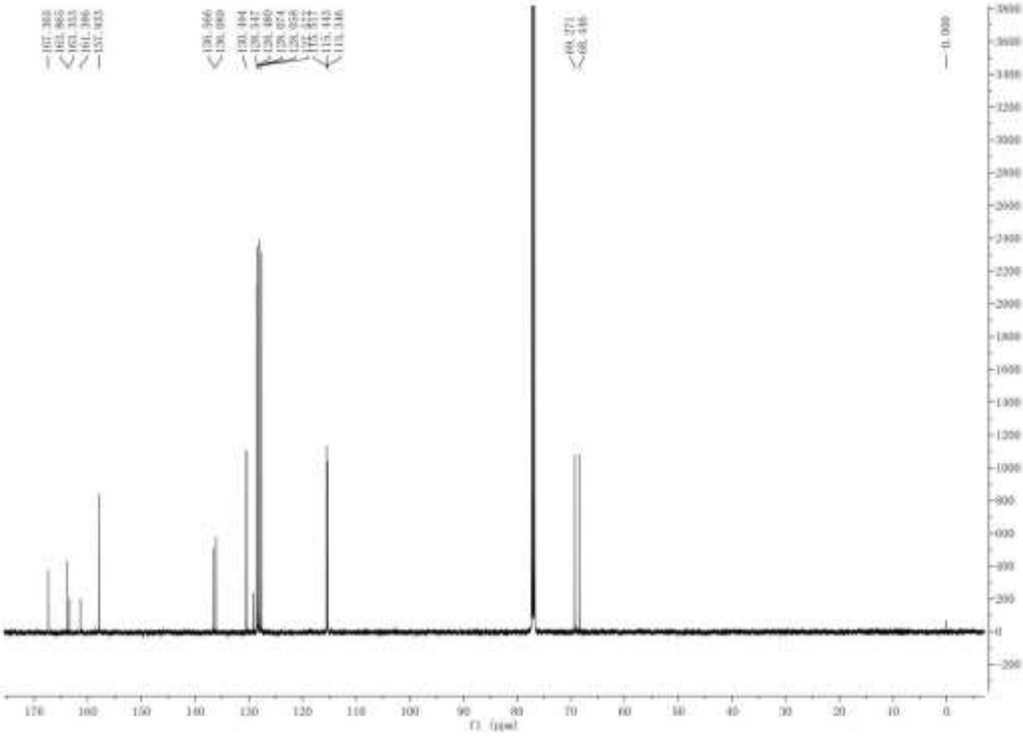

Figure S80. ^{13}C NMR spectrum of **22e**

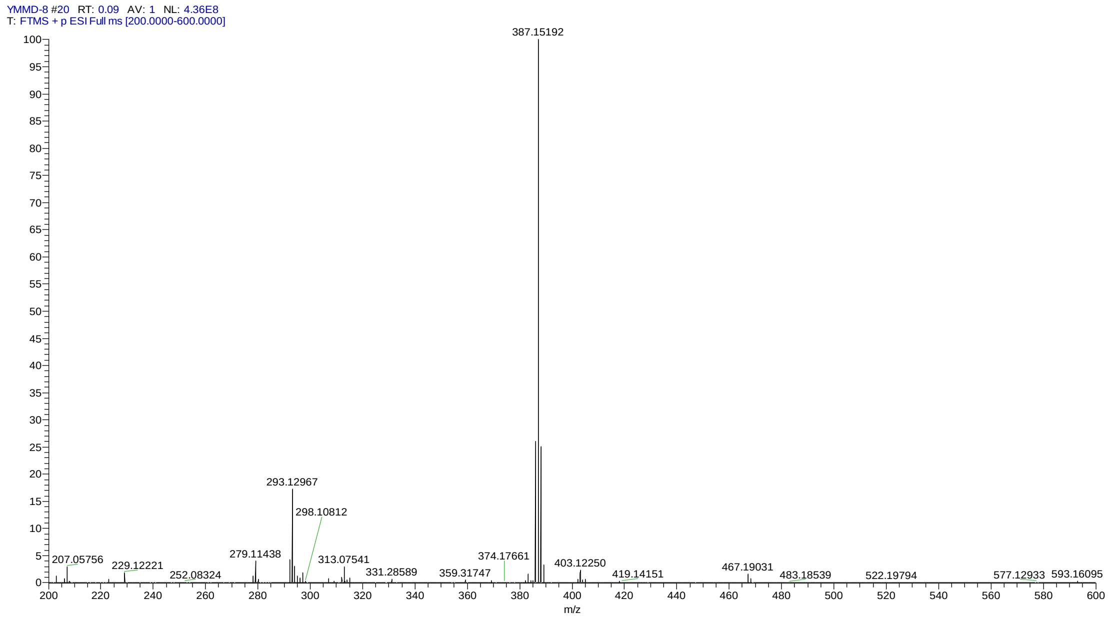

Figure S81. HRMS spectrum of **22e**

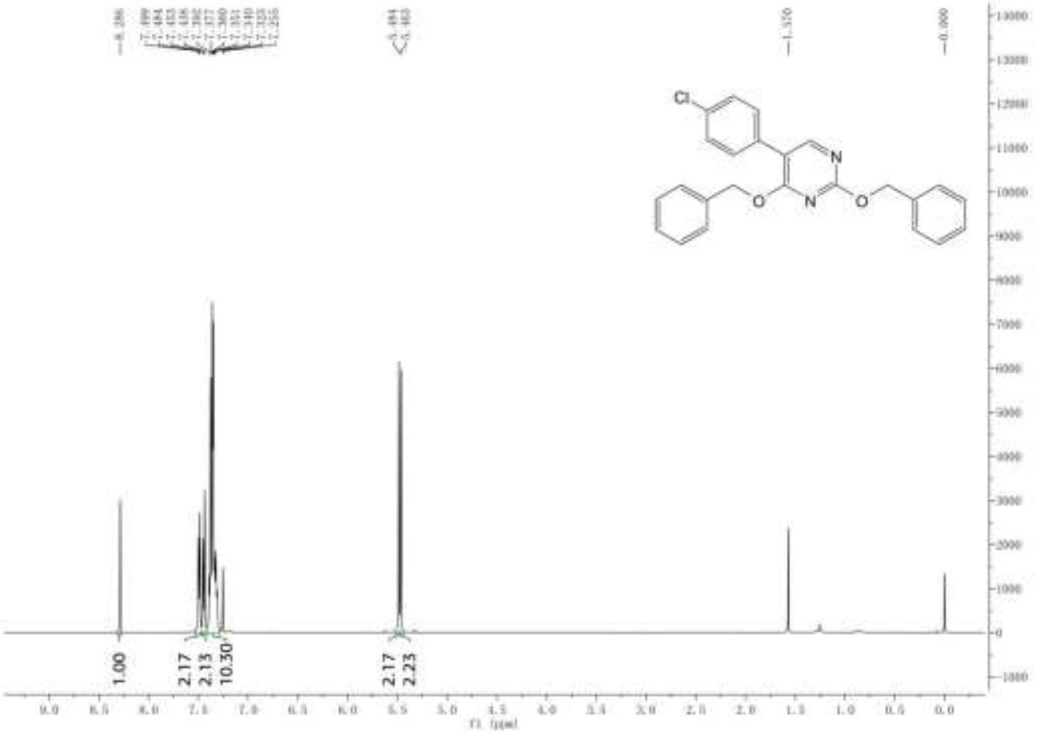

Figure S82. ^{1}H NMR spectrum of **22f**

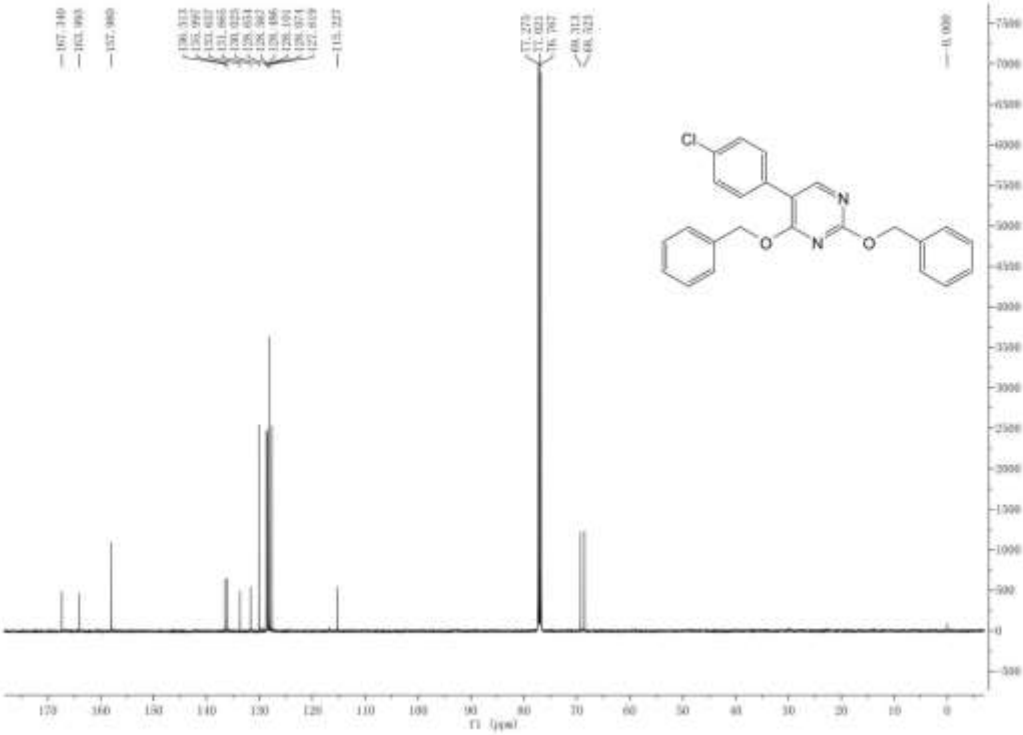

Figure S83. ^{13}C NMR spectrum of **22f**

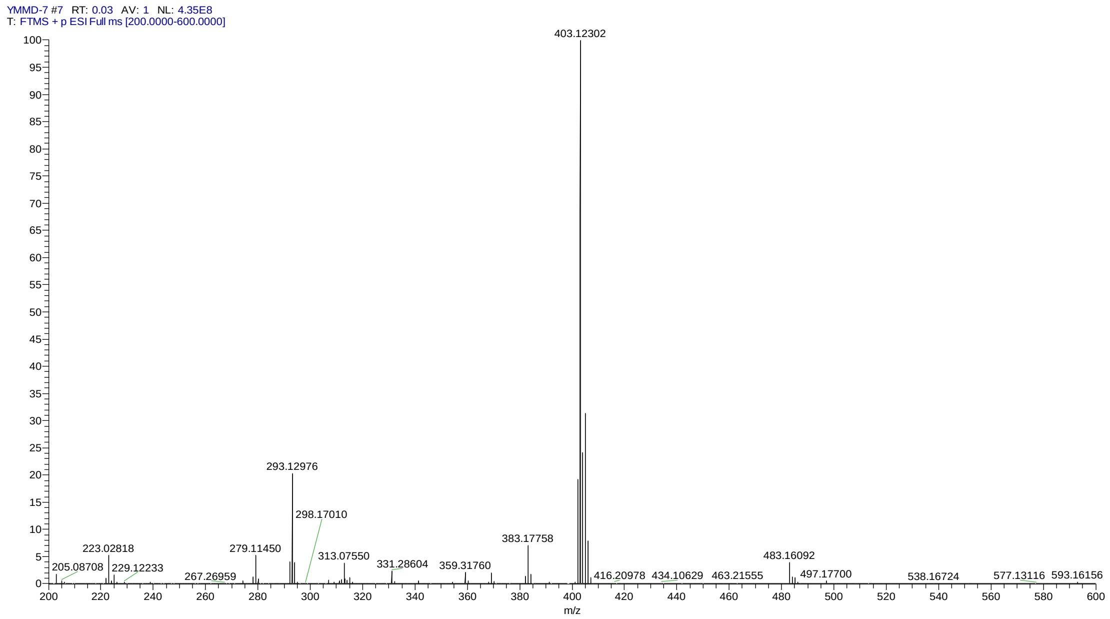

Figure S84. HRMS spectrum of **22f**

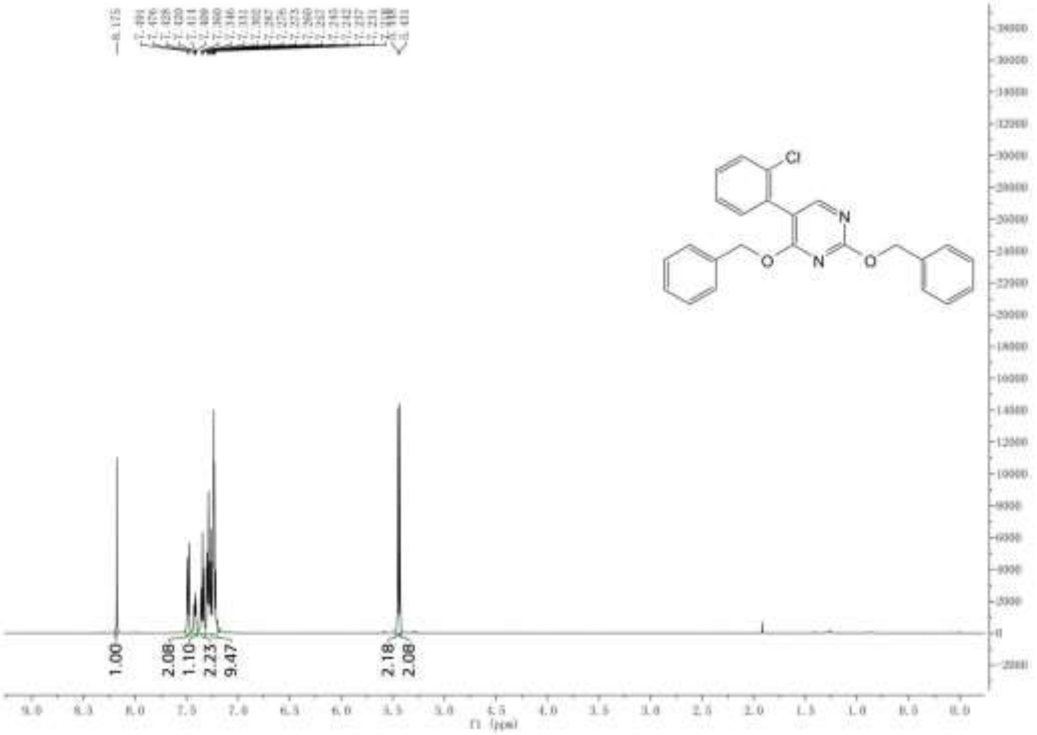

Figure S85. ^{1}H NMR spectrum of **22g**

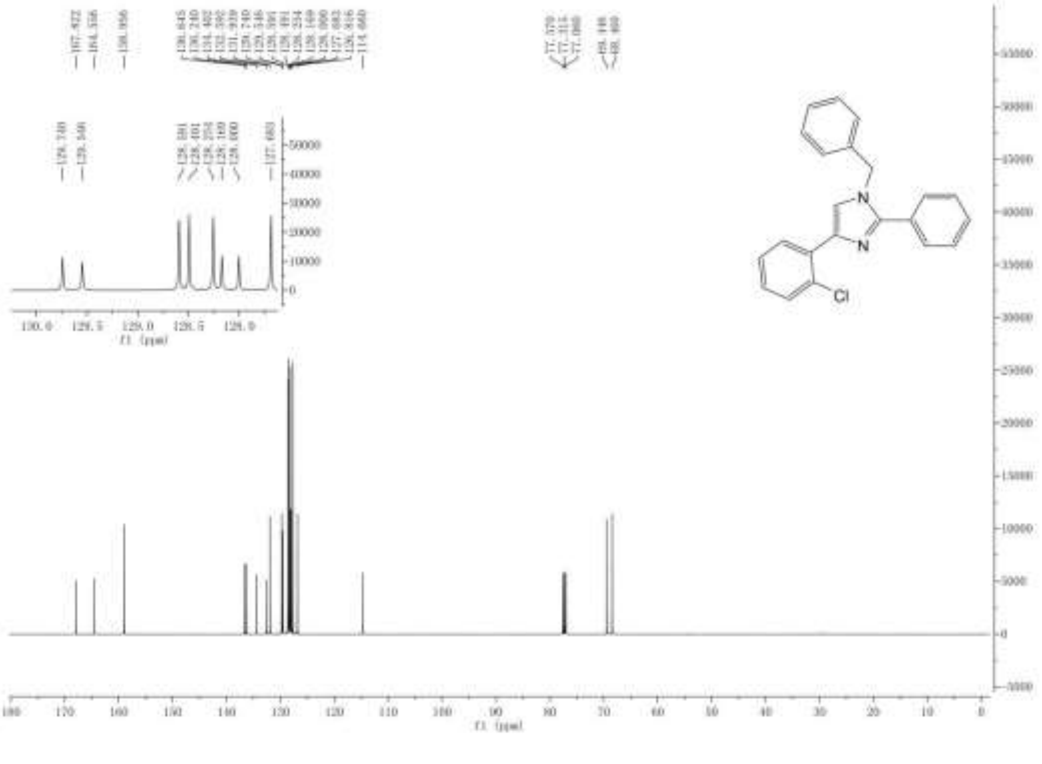

Figure S86. ^{13}C NMR spectrum of **22g**

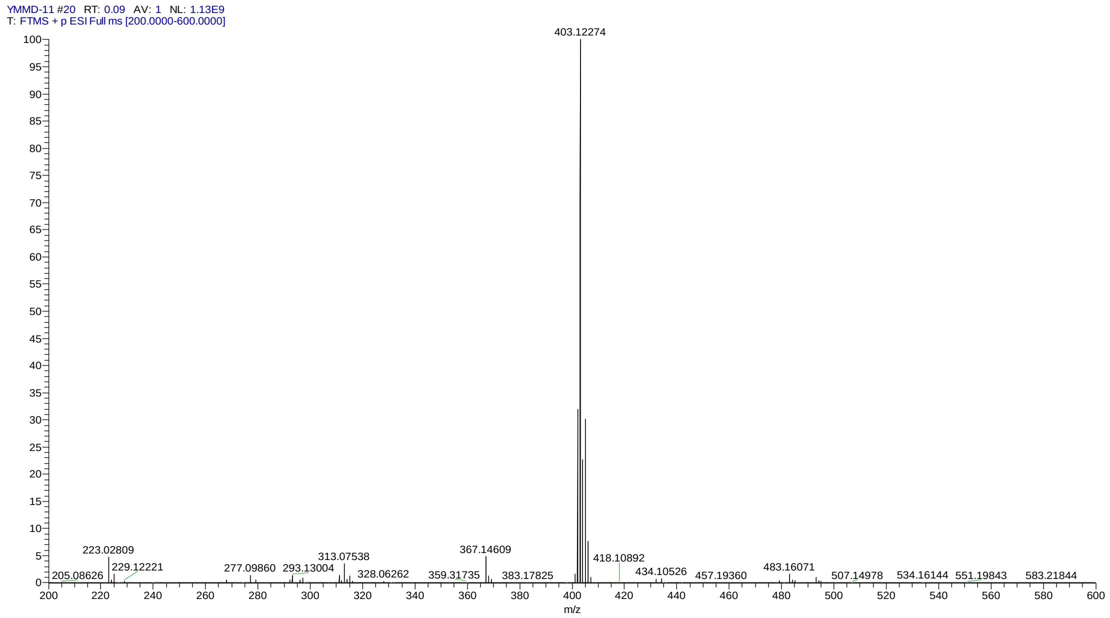

Figure S87. HRMS spectrum of **22g**

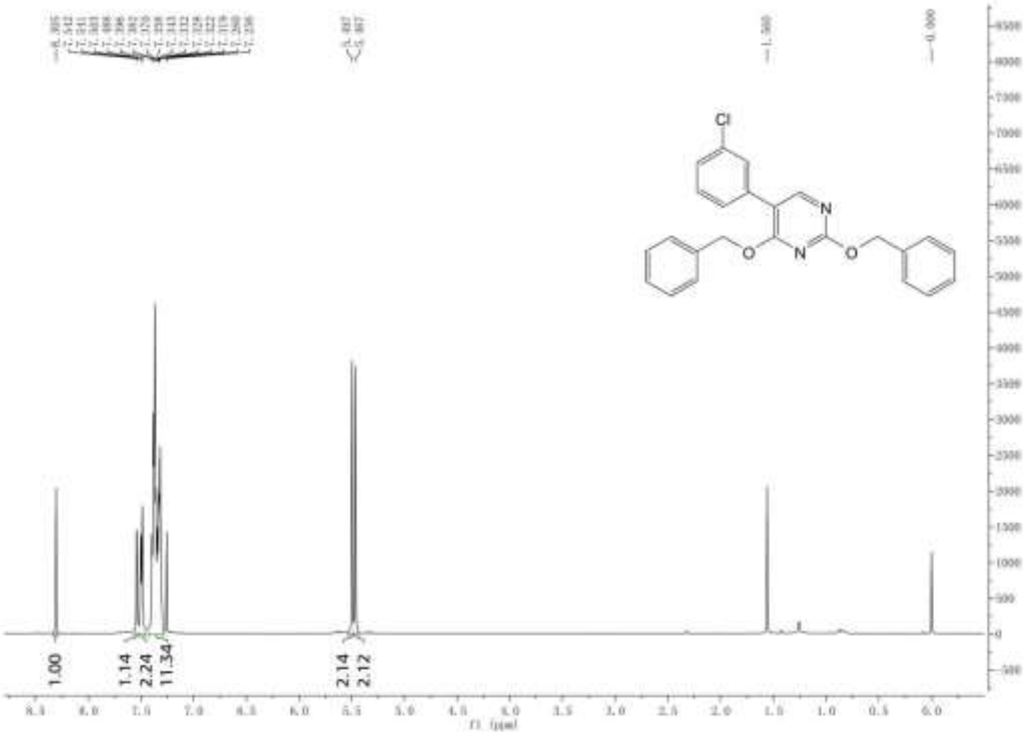

Figure S88. ^{1}H NMR spectrum of **22h**

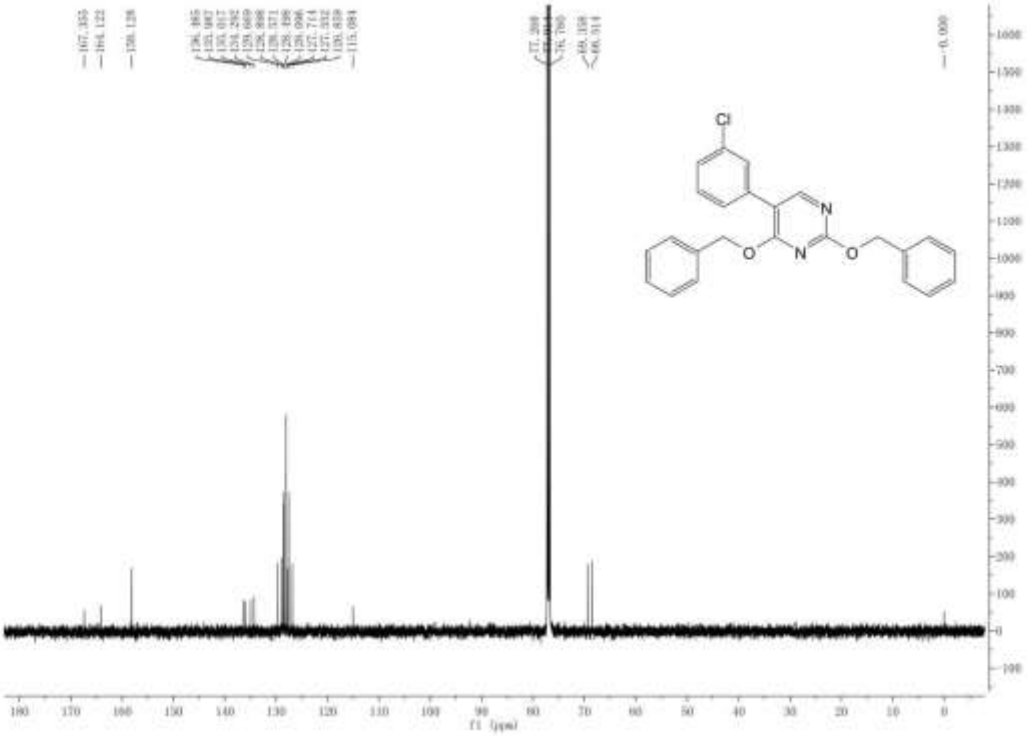

Figure S89. ^{13}C NMR spectrum of **22h**

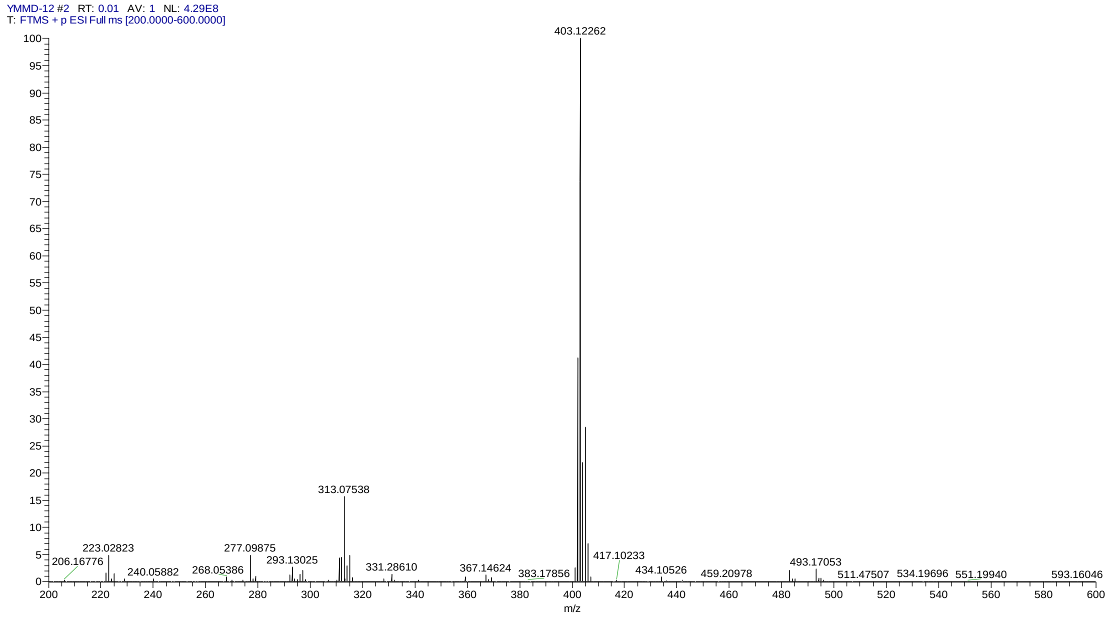

Figure S90. HRMS spectrum of **22h**

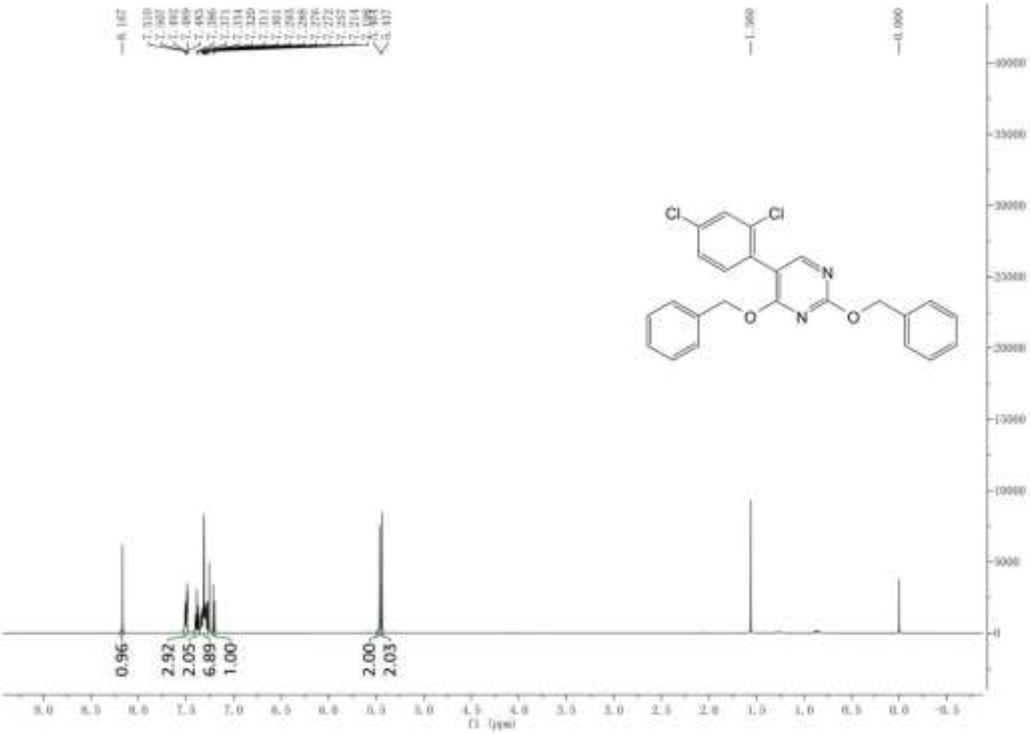

Figure S91. ^{1}H NMR spectrum of **22i**

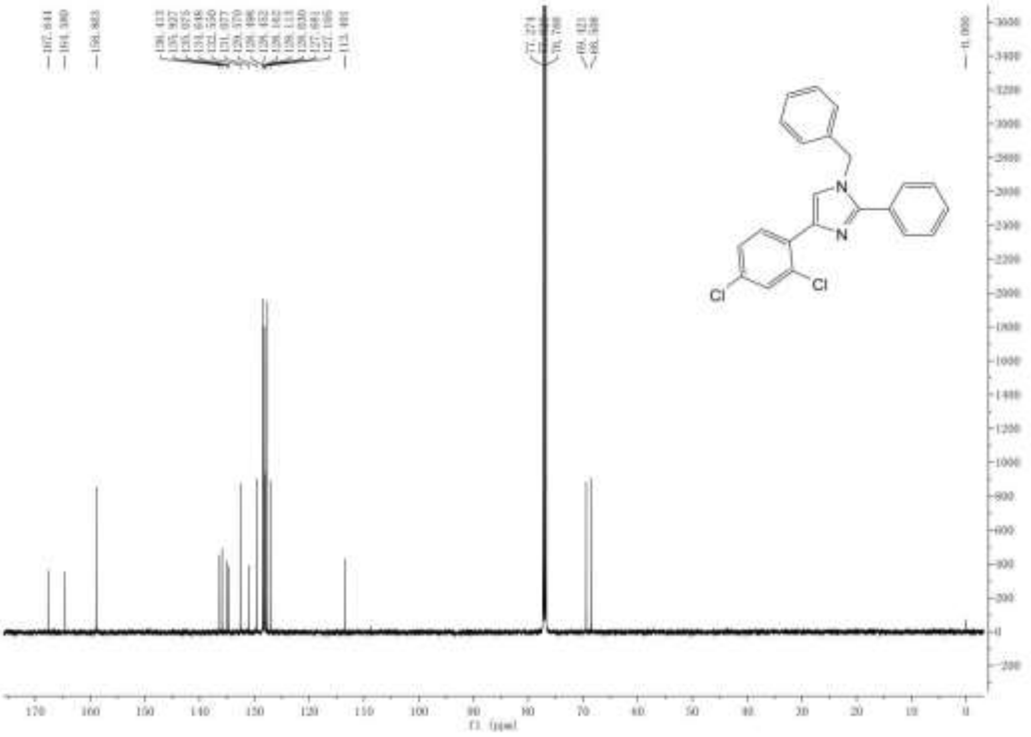

Figure S92. ^{13}C NMR spectrum of **22i**

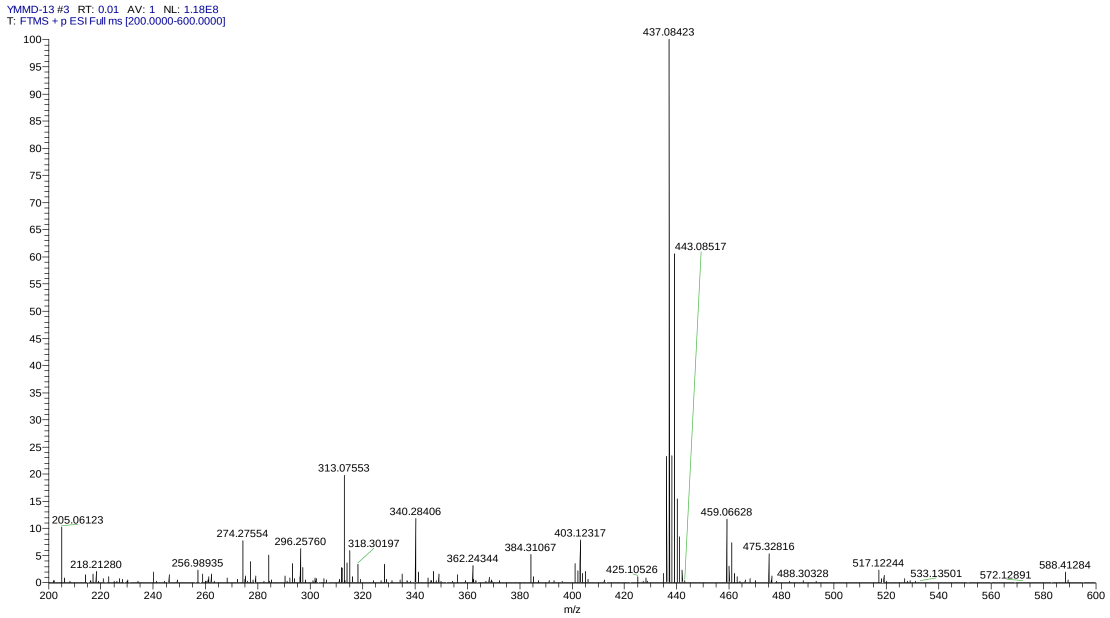

Figure S93. HRMS spectrum of **22i**

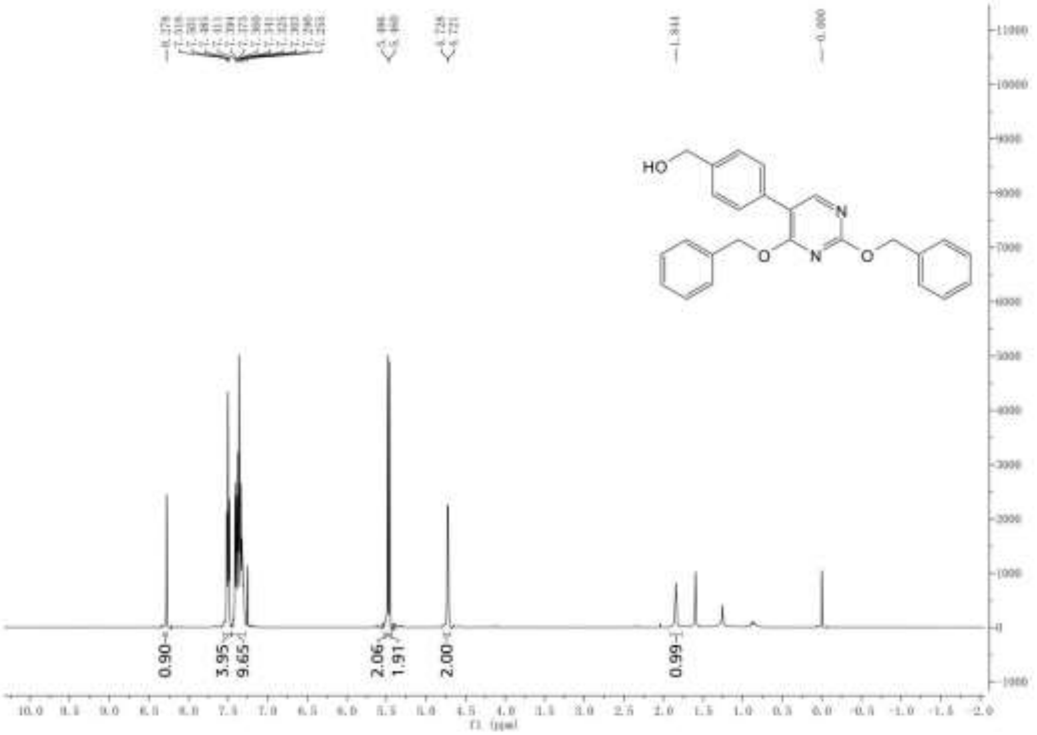

Figure S94. ^{1}H NMR spectrum of **22j**

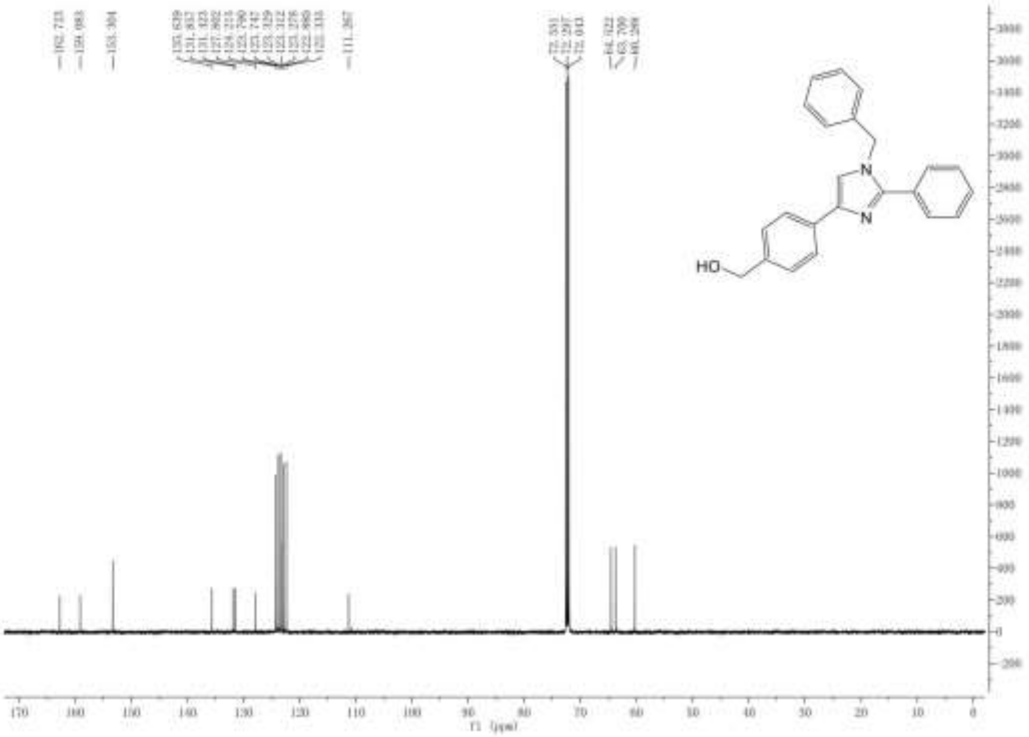

Figure S95. ^{13}C NMR spectrum of **22j**

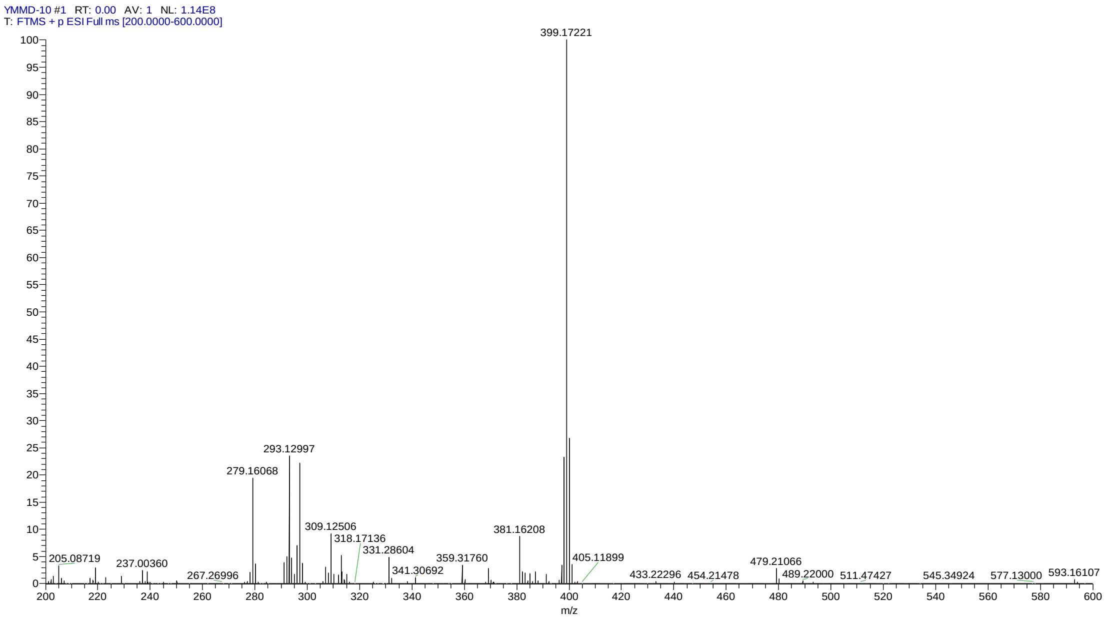

Figure S96. HRMS spectrum of **22j**

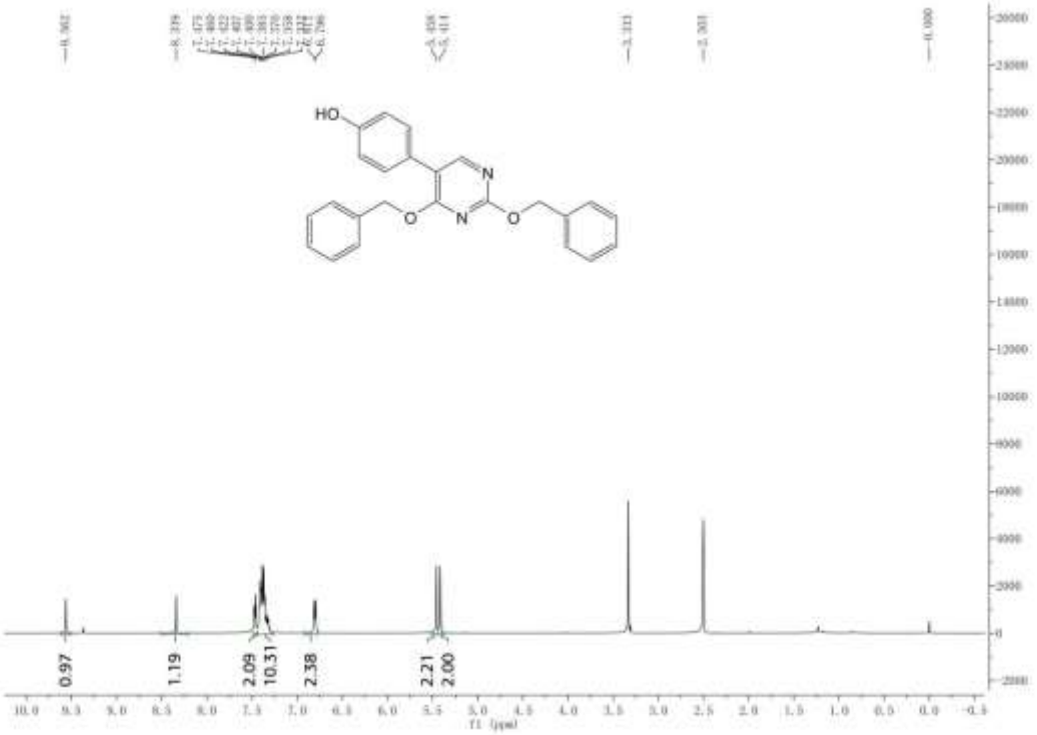

Figure S97. ^{1}H NMR spectrum of **22k**

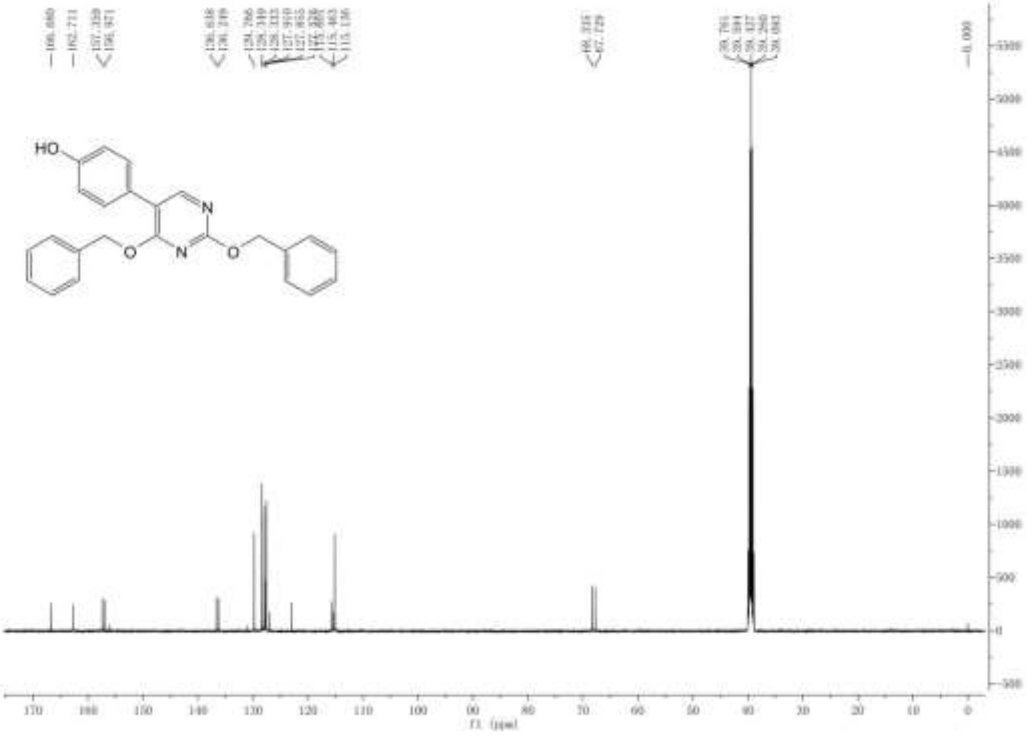

Figure S98. ^{13}C NMR spectrum of **22k**

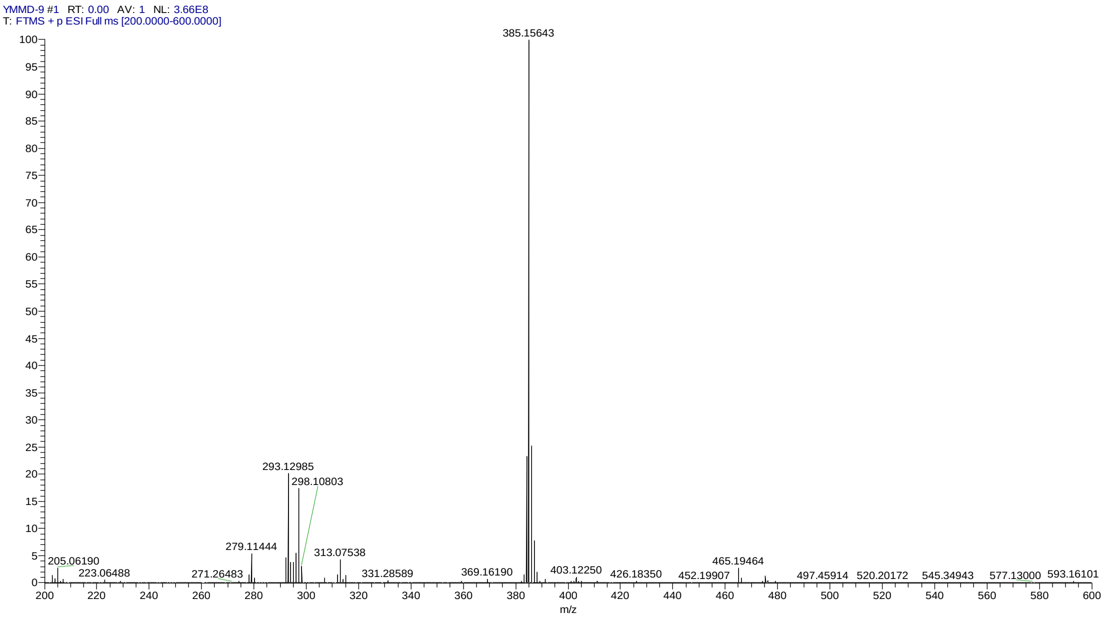

Figure S99. HRMS spectrum of **22k**

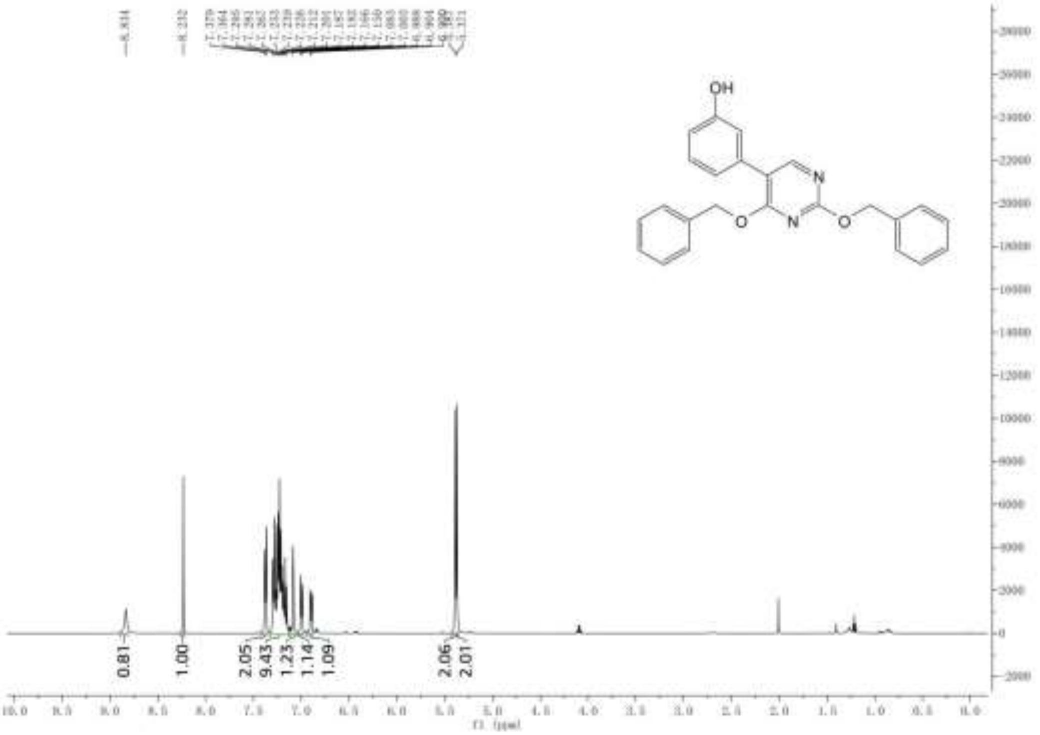

Figure S100. ^{1}H NMR spectrum of **22l**

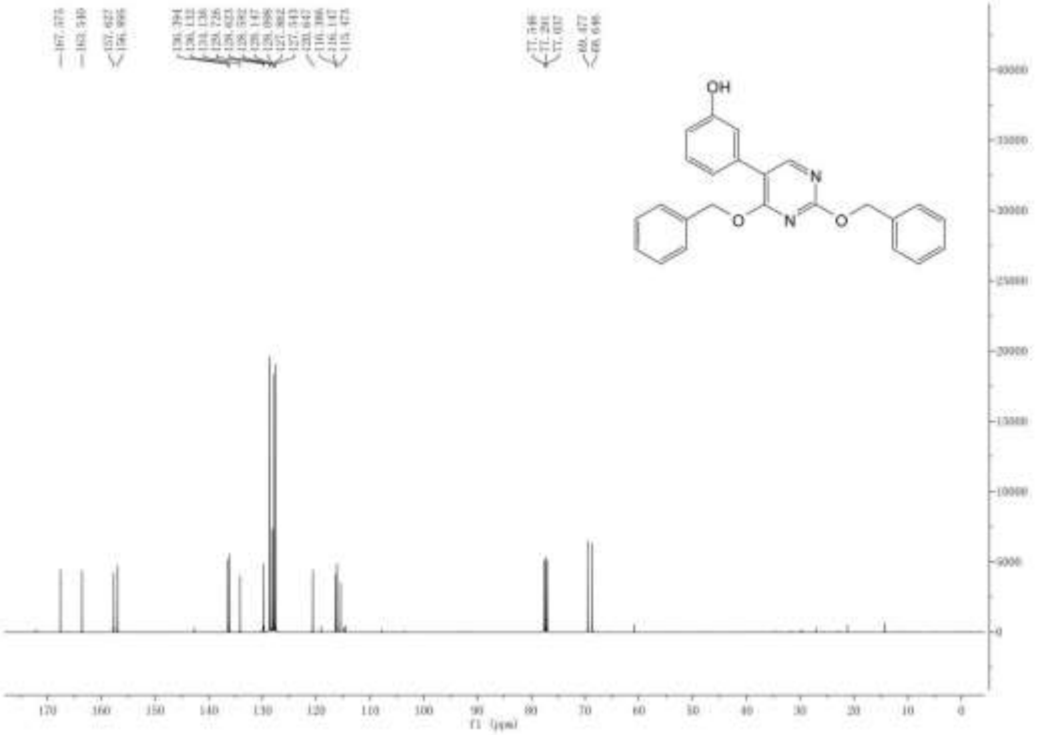

Figure S101. ^{13}C NMR spectrum of **22l**

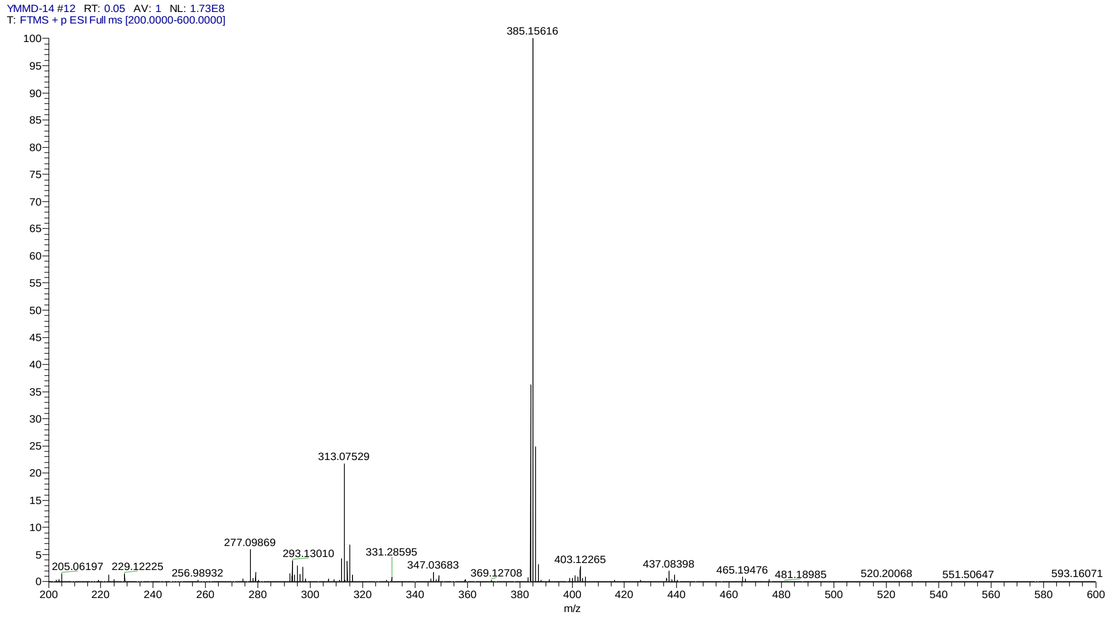

Figure S102. HRMS spectrum of **22l**

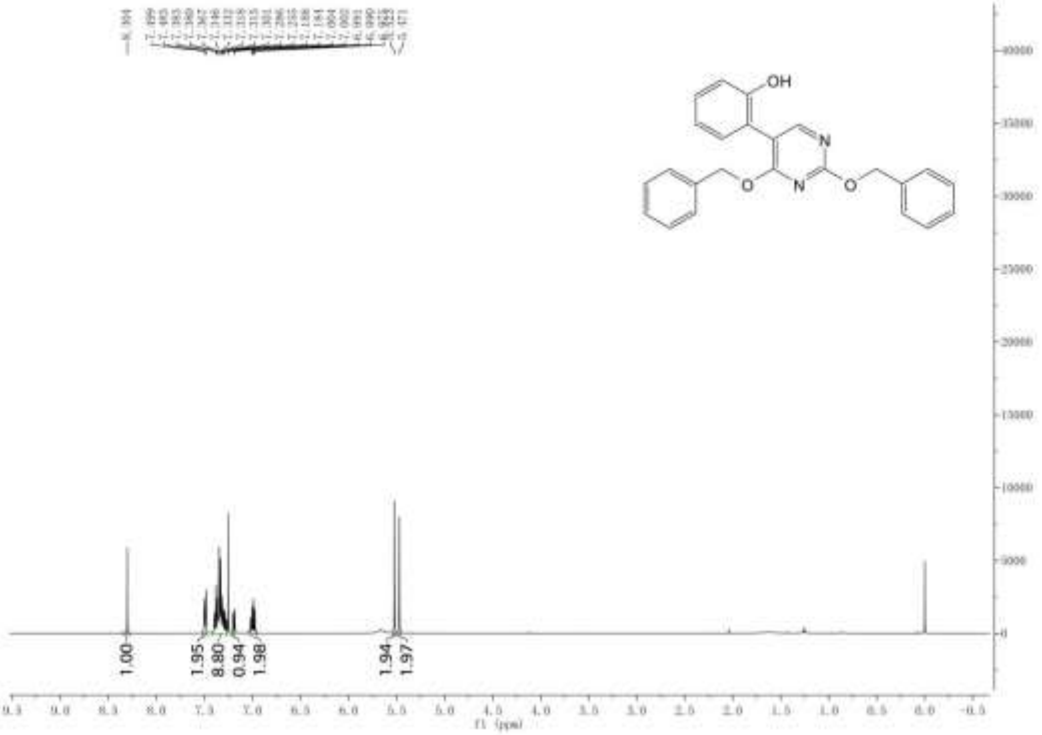

Figure S103. ^{1}H NMR spectrum of **22m**

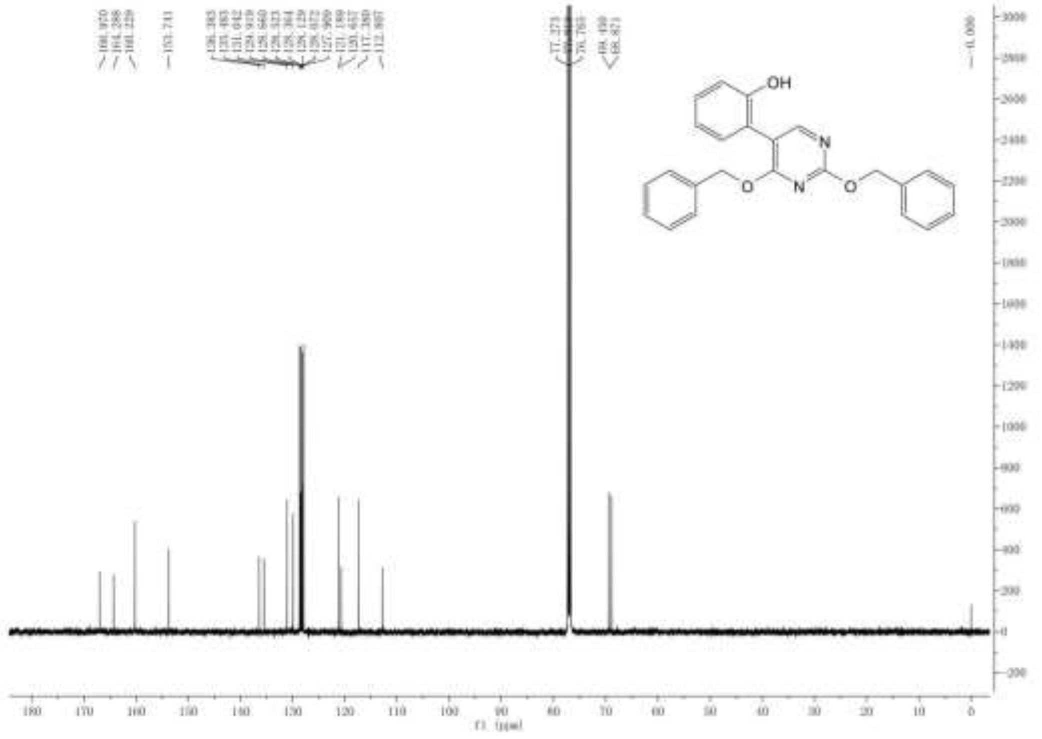

Figure S104. ^{13}C NMR spectrum of **22m**

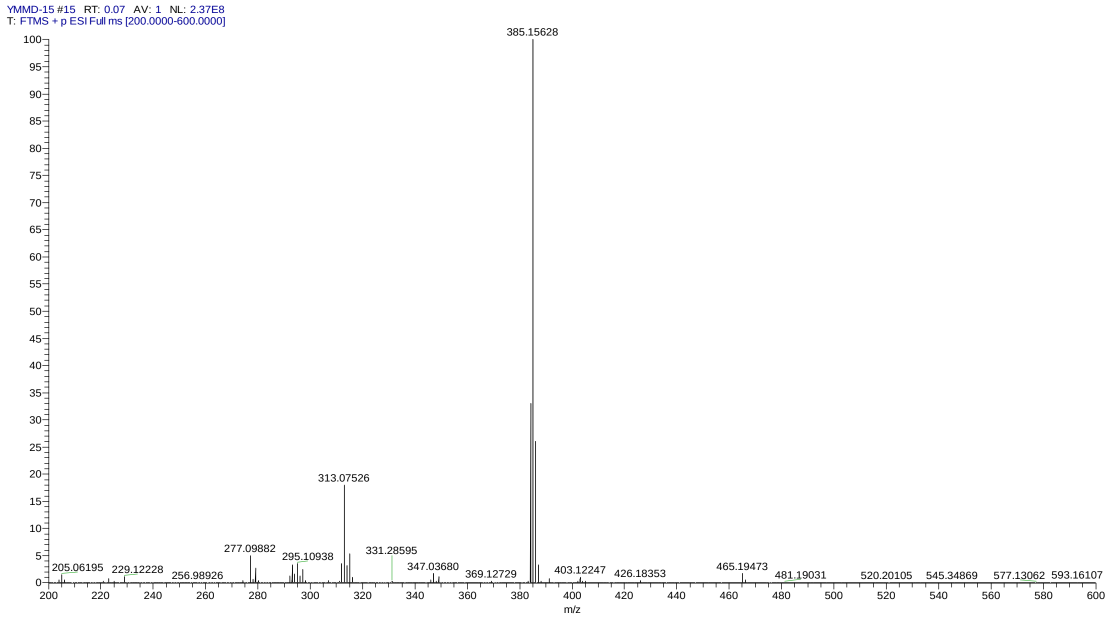

Figure S105. HRMS spectrum of **22m**

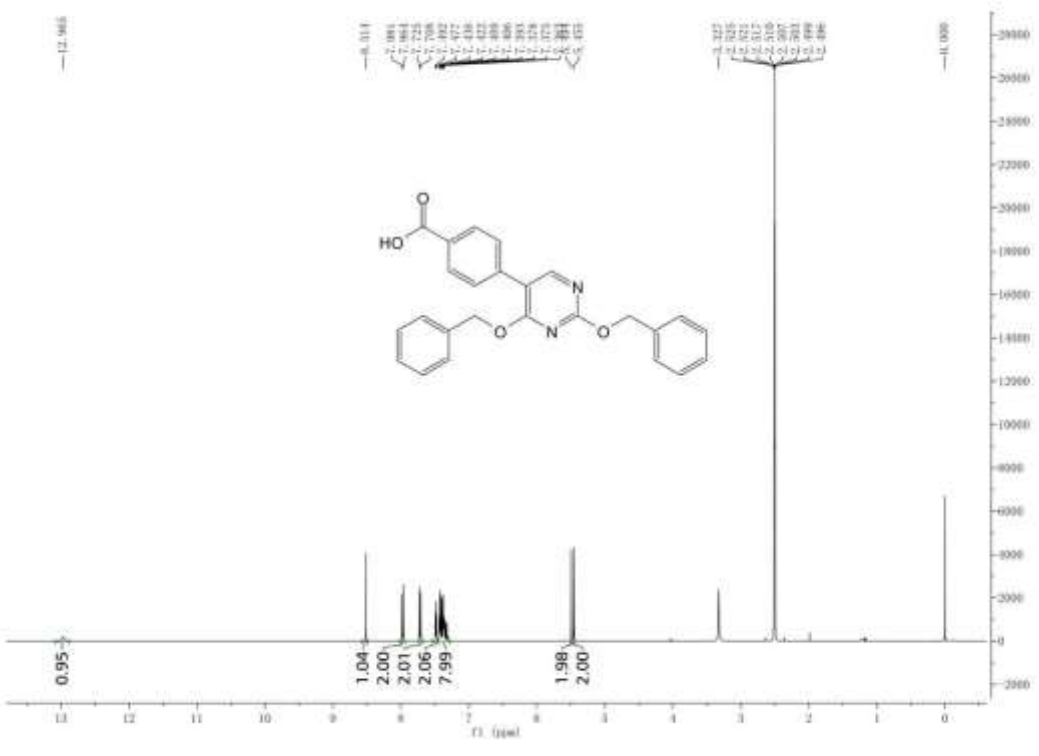

Figure S106. ^{1}H NMR spectrum of **22n**

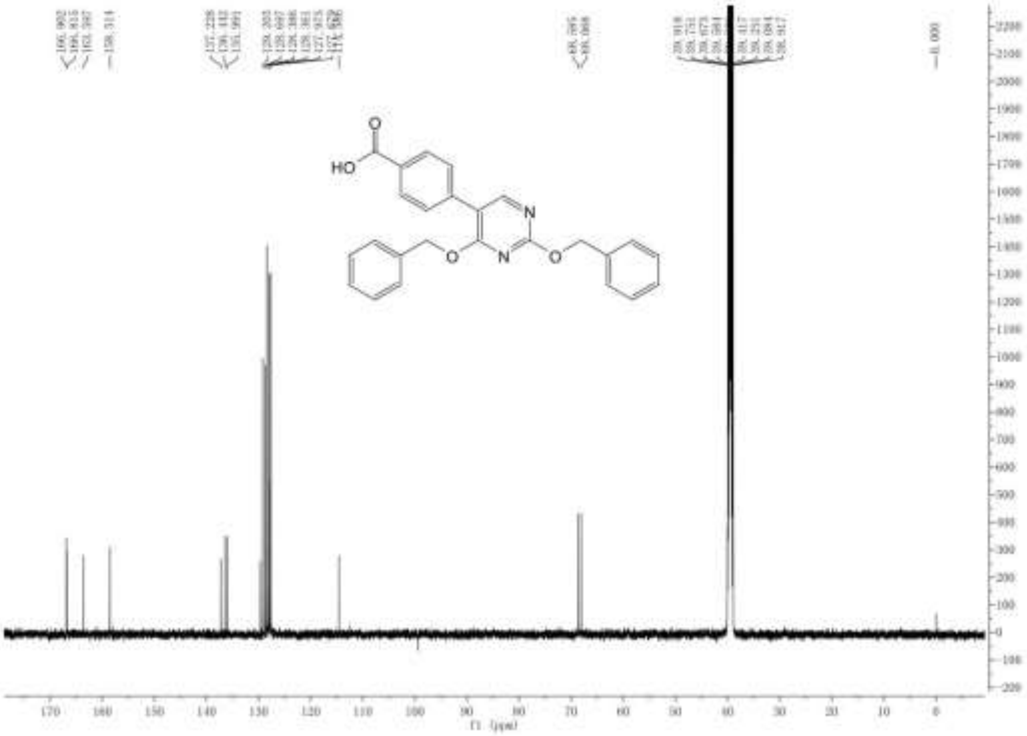

Figure S107. ^{13}C NMR spectrum of **22n**

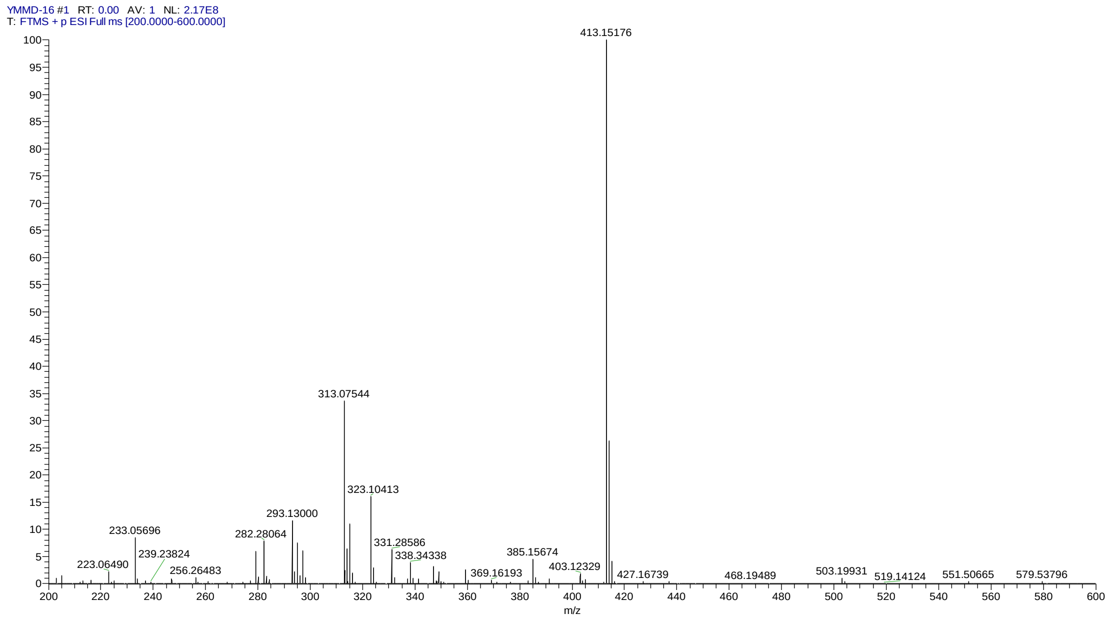

Figure S108. HRMS spectrum of **22n**

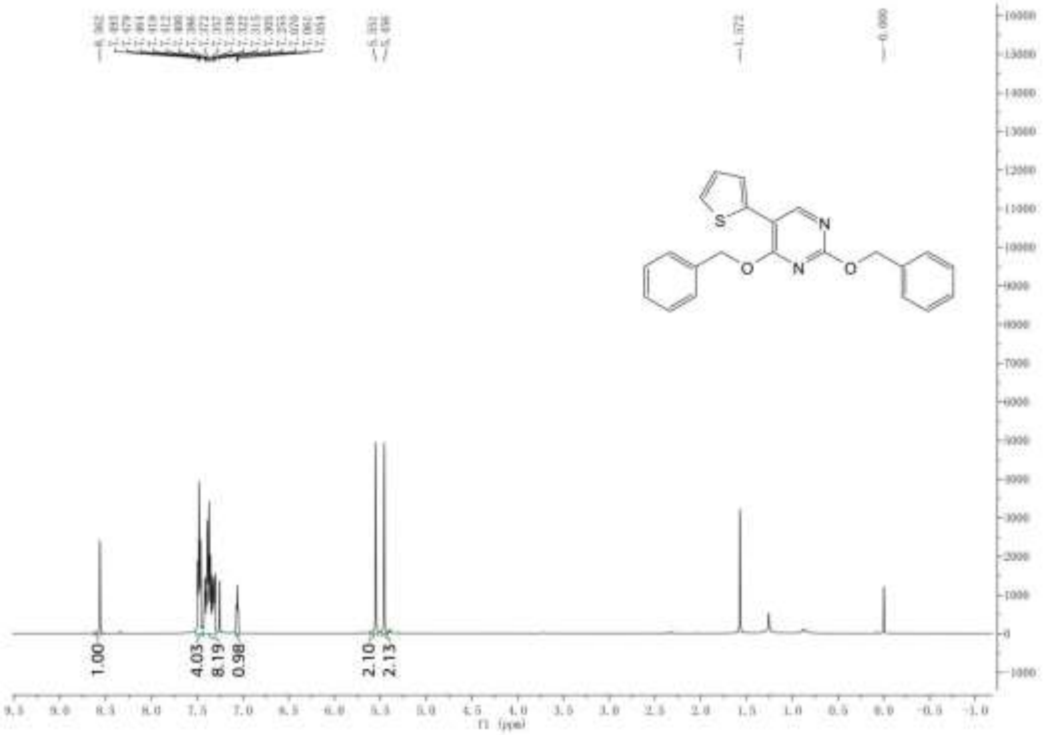

Figure S109. ^{1}H NMR spectrum of **22o**

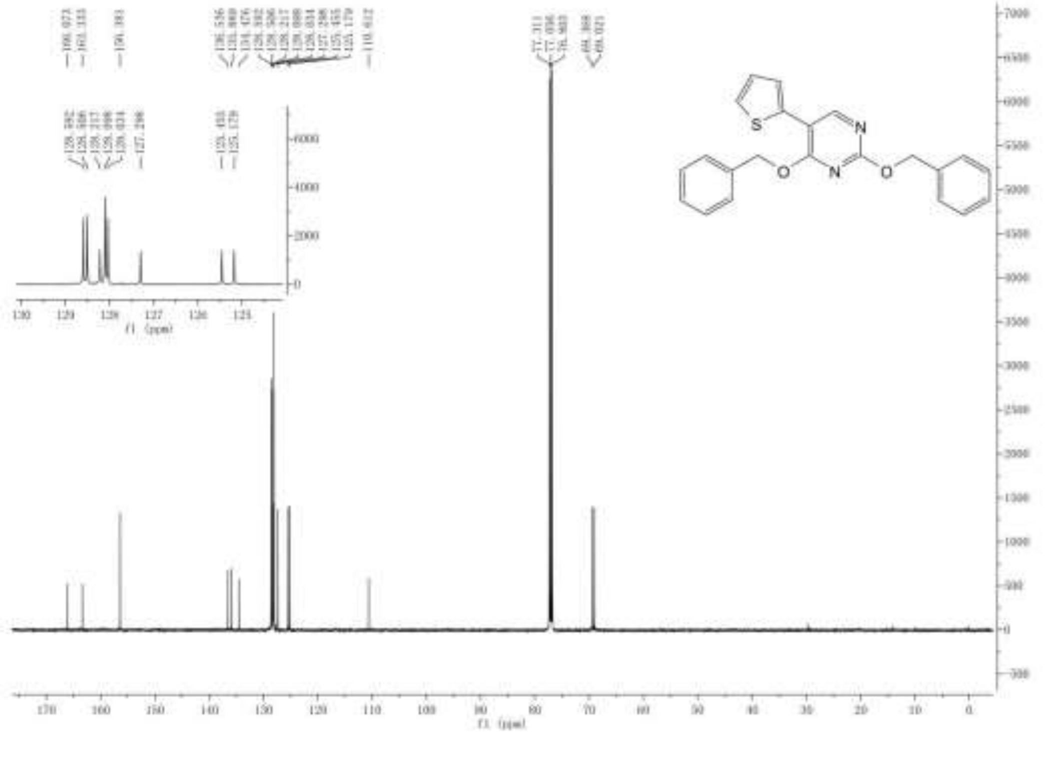

Figure S110. ^{13}C NMR spectrum of **22o**

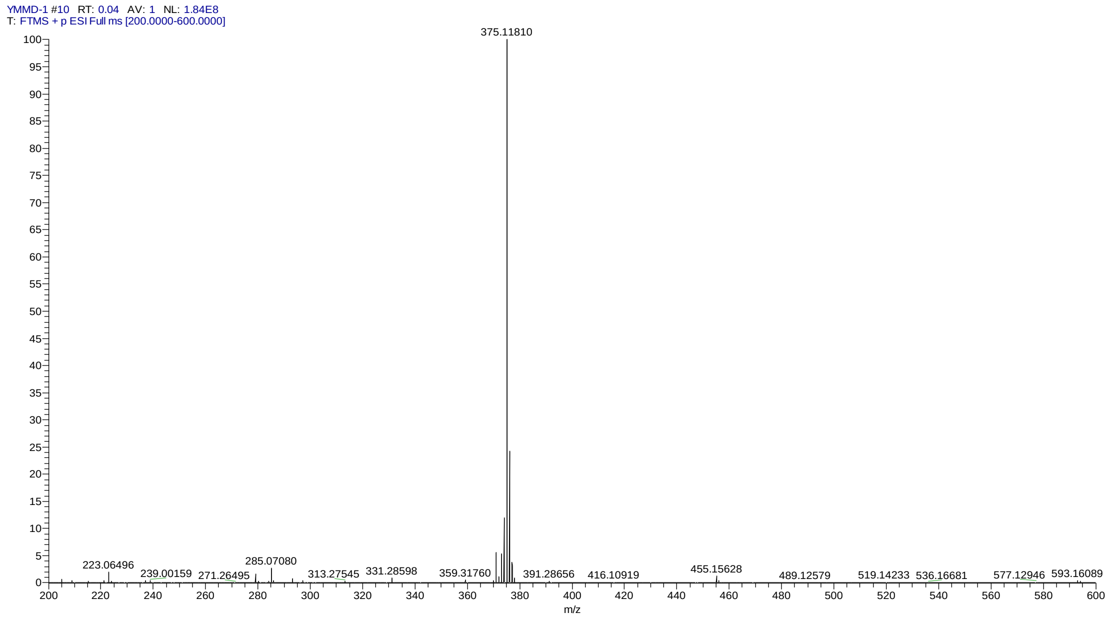

Figure S111. HRMS spectrum of **22o**

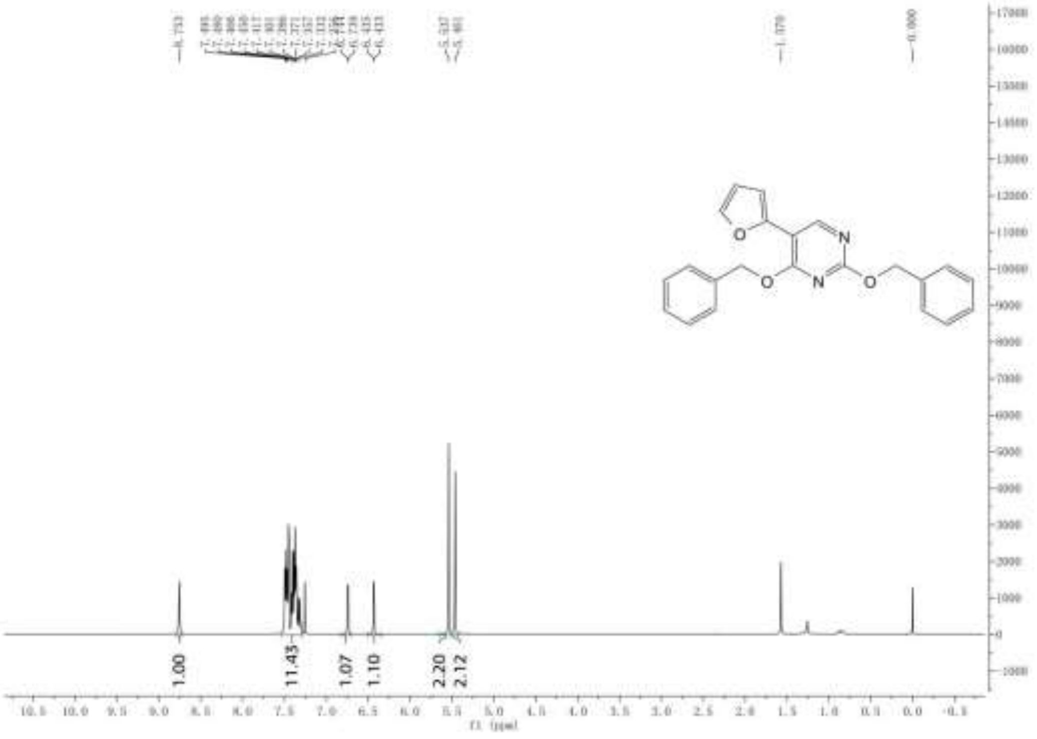

Figure S112. ^{1}H NMR spectrum of **22p**

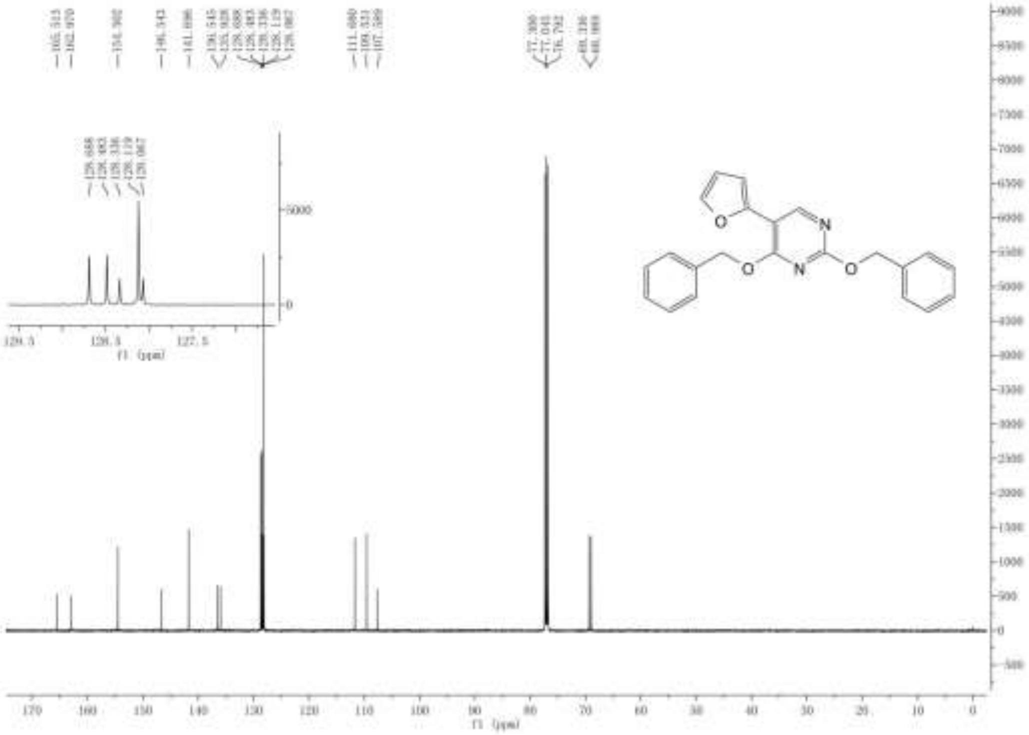

Figure S113. ^{13}C NMR spectrum of **22p**

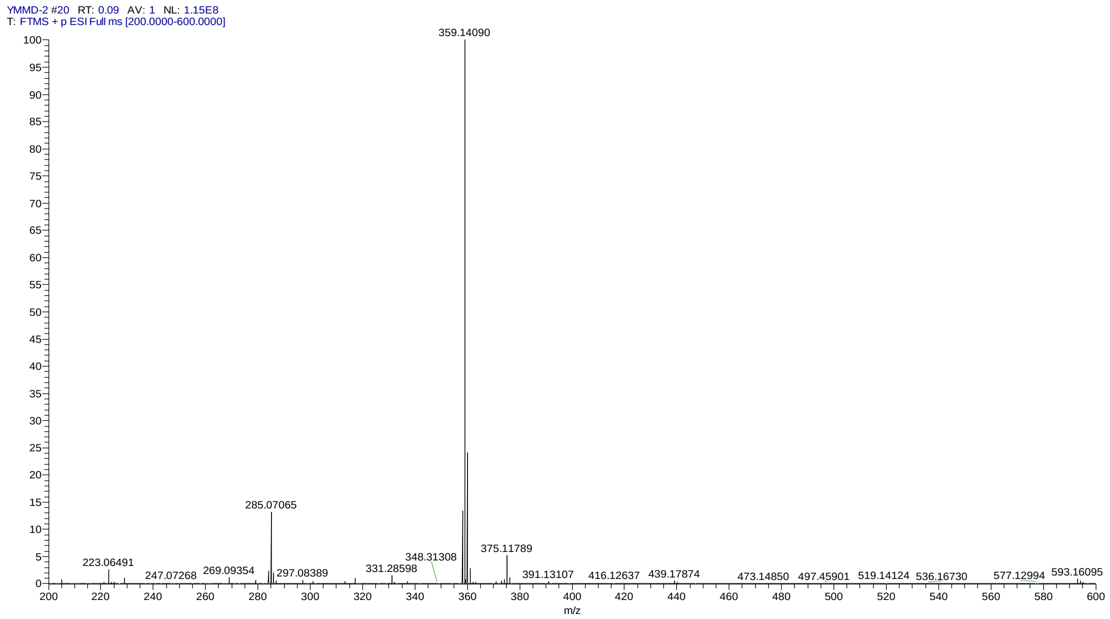

Figure S114. HRMS spectrum of **22p**

2. FP assay resluts

Table S1. FP asasy data

Comp.	FP IC_{50}（μM）
**17-AAG**	0.67±0.15
**16l**	0.57±0.06
**16j**	0.61±0.03
**16m**	0.62±0.11
**22k**	0.21±0.03
**22l**	0.58±0.11

3. Molecular docking of HSP90α (PDB code: 1YET) with geldanamycin

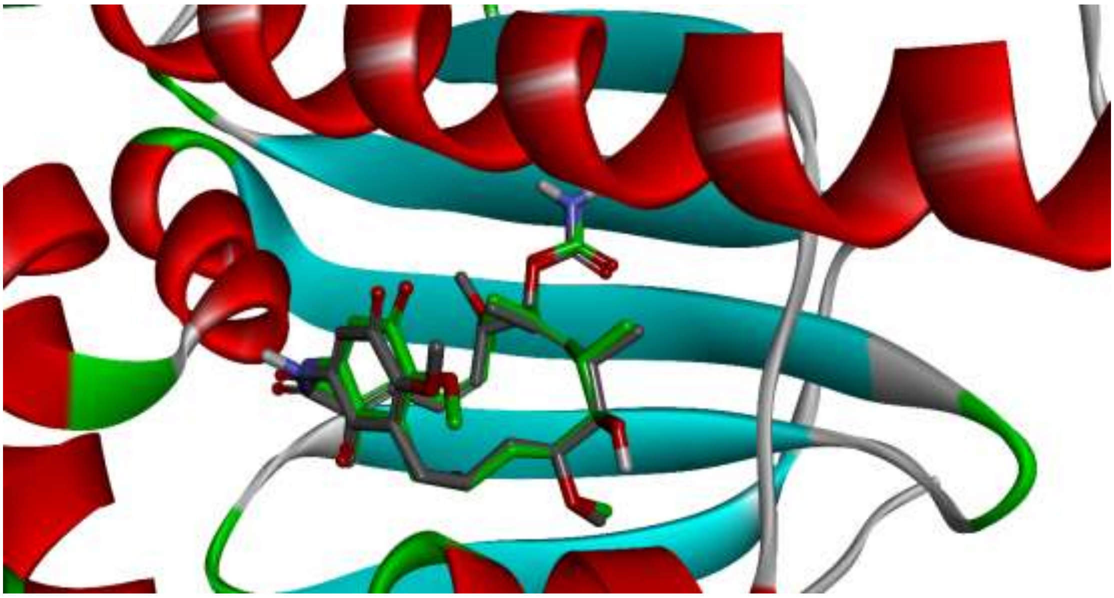

Figure S115. Superposition of the experimental pose of geldanamycin (in green) and the top-ranked pose from molecular docking (in grey) with RMSD = 0.70 Å.
